# Evaluation of the association between MREPT-derived conductivity and IVIM-derived ISF-related metrics in the brains of patients with cognitive impairments

**DOI:** 10.3389/fnagi.2026.1828803

**Published:** 2026-06-10

**Authors:** Jiwon Jung, Nitish Katoch, Hak Young Rhee, Yu Jin Jung, Yoon Kim, Soonchan Park, Chang-Woo Ryu, Kisoo Kim, Geon-Ho Jahng

**Affiliations:** 1 Department of Electronics and Information Convergence Engineering, College of Electronics and Information, Kyung Hee University, Yongin-Si, Gyeonggi-do, Republic of Korea; 2 MR Clinical Science, Philips Healthcare, Seoul, Republic of Korea; 3 Department of Neurology, Kyung Hee University Hospital at Gangdong, Kyung Hee University College of Medicine, Seoul, Republic of Korea; 4 Department of Radiology, Kyung Hee University Hospital at Gangdong, Kyung Hee University College of Medicine, Seoul, Republic of Korea

**Keywords:** association, cognitive impairment, high-frequency conductivity, interstitial fluid diffusion, interstitial fluid volume fraction, physics AI model

## Abstract

**Background:**

Alterations in the ionic microenvironment and interstitial fluid (ISF) dynamics are increasingly recognized as early pathological events in Alzheimer’s disease (AD). However, the *in vivo* relationship between bulk tissue electrical properties and ISF mobility remains unclear.

**Purpose:**

This study aimed to investigate the relationship between magnetic resonance electrical properties tomography (MREPT)-derived high-frequency conductivity (HFC) and intravoxel incoherent motion (IVIM)-derived ISF-related metrics in patients with cognitive impairments across the cognitively normal to AD spectrum.

**Methods:**

A total of 161 participants (26 cognitively normal (CN), 78 with amnestic mild cognitive impairment (MCI), and 57 with AD) underwent 3.0 T MRI. HFC maps were reconstructed using phase-based MREPT. ISF-related diffusion (D_ISF_) and volume fraction (F_ISF_) indices were extracted from multi-b-value IVIM data using an unsupervised physics-informed neural network (PINN) approach. Voxel-based and region-of-interest (ROI) statistical analyses, including ANCOVA, partial correlations, and robust multiple regressions, were performed with adjustments for age and sex.

**Results:**

Along the disease progression from CN to AD, D_ISF_ significantly decreased, while F_ISF_ and HFC increased. Partial correlation analyses revealed that these alterations were significantly correlated with age and cognitive decline. Furthermore, MREPT-derived HFC exhibited region-specific correlations with IVIM-derived ISF-related measures, most notably in the bilateral corpus callosum and thalamus. Robust multiple regression analyses revealed limited, region-specific independent associations between ISF indices and HFC, with the most robust finding observed for D_ISF_ in the left thalamus, whereas F_ISF_-related associations did not survive correction, and F_PAR_ showed the most consistent independent association in the volume-fraction model.

**Conclusion:**

The region-specific associations observed between MREPT-derived HFC and IVIM-derived ISF-related metrics in select AD-vulnerable regions, particularly the independent D_ISF_–HFC association in the left thalamus, suggest that neurodegeneration may be linked with local ionic and microenvironmental compartmental shifts. These findings require replication in larger, independent cohorts. Combining MREPT and IVIM-based ISF-related metrics may provide a non-invasive imaging framework to probe these complex microenvironmental alterations in cognitive impairment.

## 1 Introduction

Alzheimer’s disease (AD) is a progressive neurodegenerative disorder and the most common cause of dementia. Patients with AD are characterized by the accumulation of amyloid-beta (Aβ) plaques and tau tangles in the brain, leading to neuronal loss and brain atrophy (Querfurth and LaFerla, 2010). This biomarker-based view supports the notion that AD pathophysiology begins years before overt dementia, underscoring the importance of sensitive markers for early detection and longitudinal monitoring (Jack et al., 2010). Mild cognitive impairment (MCI) is a condition characterized by noticeable cognitive decline greater than expected for a person’s age but not severe enough to significantly interfere with daily life (Gauthier et al., 2006). MCI represents a clinically relevant stage within this continuum because it often captures individuals at elevated risk for progression to AD dementia. The amnestic MCI (aMCI) primarily affects memory and is usually considered a precursor to Alzheimer’s disease.

The glymphatic system is a network that facilitates the removal of waste products from the brain (Iliff et al., 2012). Brain fluid or neurofluid homeostasis is increasingly recognized as a key determinant of protein clearance and neuroinflammatory balance in aging and neurodegeneration (Tarasoff-Conway et al., 2015). These fluids play crucial roles in maintaining brain health and function. Cerebrospinal fluid (CSF) cushions the brain, removes waste, provides nutrients, and flows through the ventricles and around the brain and spinal cord. The brain’s extracellular space contains interstitial fluid (ISF) that is derived from blood crossing the blood–brain barrier and fluid from the CSF (Kaur et al., 2021). In AD, impaired ISF dynamics — including reduced clearance of waste products such as Aβ — are linked to the condition’s hallmark protein accumulations (amyloid plaques and tau tangles) (Guo et al., 2025).

Brain ISF mapping using MRI involves advanced imaging techniques to visualize and analyze the movement and distribution of ISF within the brain. Contrast agents are conventionally administered to monitor their passage through the brain over time (Iliff et al., 2013). In addition, the intravoxel incoherent motion (IVIM) MRI technique is used without the injection of a contrast agent (Le Bihan et al., 1988). In the IVIM technique, multi-b-value diffusion MRI can be partitioned into more than the conventional two IVIM components. In the IVIM technique, multi-b-value diffusion MRI can be divided into more than the conventional two IVIM components. Spectral analyses and tri-exponential modeling, in particular, suggest the presence of an additional intermediate diffusion component with diffusivity between parenchymal diffusion and microvascular pseudo-diffusion. This component is interpreted as reflecting ISF in extracellular and perivascular spaces (Wong et al., 2020).

Electrical conductivity in the brain refers to the ability of brain tissue to conduct electrical currents. This conductivity varies across different types of brain tissue, including gray matter, white matter, and cerebrospinal fluid, due to differences in water content and ion concentration. Tissue conductivity primarily reflects the concentration and mobility of ions in both intra- and extracellular water compartments, making it sensitive to microstructural and biochemical changes that affect ionic content, water fraction, and membrane integrity (Lee et al., 2020). Magnetic Resonance Electrical Property Tomography (MREPT) is an imaging technique that seeks to map the electrical properties of tissues using MRI (Hafalir et al., 2014; Gurler and Ider, 2017). For MREPT, a sinusoidal current is applied as the radiofrequency (RF) magnetic field (B1) of the MRI scanner to a human brain, non-invasively. MREPT reconstructs maps of tissue electric conductivity, which is called a high-frequency conductivity (HFC) or the Larmor frequency conductivity. HFC is a frequency-dependent tissue bulk conductivity (Katscher and Den Berg, 2017).

High-frequency current tends to pass through cell membranes, and HFC is not obstructed by them. Since ionic homeostasis and tissue microstructure can be altered in neurodegeneration, conductivity mapping has been proposed as a potential imaging marker to characterize disease-related tissue changes (Zhang et al., 2014). Conductivity values were increased in patients with AD and MCI compared with CN, especially in the insular and hippocampus areas (Park et al., 2022; Hong et al., 2024). Collectively, these findings support the clinical relevance of conductivity-derived metrics as complementary markers that may reflect disease-related alterations in tissue microenvironment and ionic dynamics, and motivate further evaluation of conductivity mapping for understanding and monitoring cognitive decline along the MCI–AD spectrum (Park et al., 2022).

Although previous studies have reported changes in ISF-related measures in patients with cognitive impairment and differences in brain conductivity measured with MREPT in AD and MCI, the association between IVIM-derived ISF-related metrics and MREPT-derived conductivity has not been thoroughly investigated. Since tissue conductivity is influenced by ionic concentration and mobility in extracellular water compartments, and IVIM-derived ISF-related indices reflect alterations in extracellular and perivascular fluid dynamics, their relationship may offer complementary insights into local microenvironmental changes across the AD spectrum. Therefore, we examined the association between MREPT-derived brain conductivity and IVIM-derived ISF-related metrics in patients with cognitive impairments across the MCI-AD spectrum. Additionally, we analyzed how these MRI measures vary across diagnostic groups and how they relate to age and cognitive status using voxel-based and ROI-based analyses.

## 2 Materials and methods

### 2.1 Participants

The Institutional Review Board (IRB) approved this cross-sectional prospective study (IRB khnmc2023-02-019), and informed consent was obtained from participants between August 2023 and December 2024. Participants provided detailed medical histories and underwent neurologic examinations, standard neuropsychological testing, and MRI scans. Cognitive function was assessed using the full version of the Korean standardized neuropsychological test battery, known as the Seoul Neuropsychological Screening Battery (SNSB) (Ahn et al., 2010). Global cognitive ability was evaluated using the Korean version of the Mini-Mental State Examination (K-MMSE) and the Clinical Dementia Rating (CDR).

Inclusion criteria were: (i) older adults meeting the study age criterion (age>60 years); (ii) patients with mild or moderate Alzheimer’s disease (AD) diagnosed according to the National Institute of Neurological and Communicative Disorders and the Stroke–Alzheimer’s Disease and Related Disorders Association criteria (Blacker et al., 1994; Dubois et al., 2007); (iii) participants with amnestic MCI according to the Petersen criteria (Petersen et al., 1999; Petersen et al., 2014), and (iv) cognitively normal (CN) elderly participants. Exclusion criteria were: (i) severe AD; (ii) non-amnestic MCI; (iii) incomplete study participation or missing key assessments/imaging; (iv) incomplete MR images with artefacts (e.g., metal artefact); (v) other neurologic/psychiatric diseases; and (vi) brain parenchymal lesions such as severe small vessel disease, and tumor.

Supplementary Figure S1 illustrates the flowchart summarizing the participant selection process. Of the 210 initially screened participants, those who did not meet the eligibility criteria were excluded. Specifically, 12 participants were excluded due to other diseases, 26 were excluded due to incomplete studies, 11 were excluded due to non-amnestic MCI, 7 due to vascular or mixed dementia, and 1 due to severe AD. Among the incomplete studies, 25 participants were excluded for lack of IVIM and/or MREPT scans, and 1 was excluded for lack of SNSB. This study included a total of 161 participants: 26 CN, 78 aMCI, and 57 AD.

### 2.2 MRI acquisition

#### 2.2.1 MREPT imaging

High-frequency conductivity mapping was performed using a three-dimensional turbo spin-echo (3D-TSE) sequence (Cao et al., 2023). Imaging parameters were as follows: repetition time (TR) = 3,000 ms, echo time (TE) = 280 ms, field of view (FOV) = 240 × 240 × 190 mm^3^, acquisition matrix = 240 × 240, NSA = 1 and resolution = 1.0 × 1.0× 1.0 mm^3^. Whole-brain coverage was achieved using a 3D slab with 190 contiguous slices. Compressed SENSE acceleration with a reduction factor of 2.5 was applied. The total acquisition time was 3 min 24 s. Complex image data (real and imaginary components) were exported for phase extraction and subsequent phase-based MREPT reconstruction of high-frequency conductivity.

#### 2.2.2 IVIM imaging

For IVIM acquisition, diffusion-weighted images were obtained using a diffusion-weighted spin-echo EPI–based sequence with the following parameters: TR = 3,090 ms and TE = 116.2 ms. A total of 15 b-values were used (0, 5, 10, 15, 20, 30, 40, 50, 60, 100, 200, 500, 700, 1,000, and 1,500 s/mm^2^). IVIM images were acquired as trace-weighted (isotropically averaged) diffusion-weighted images, in which three orthogonal diffusion directions were acquired and averaged at each b-value. This trace-weighted acquisition reduces directional dependence and is suitable for scalar three-compartment IVIM modeling. Meanwhile, the dense low-b-value sampling facilitates stable estimation of the intermediate ISF-related diffusion and volume fraction components. The acquisition matrix was 96 × 85 with 52 slices, and the field of view was 220 × 114 × 220 mm^3^. The EPI factor was 51. Fat suppression was applied using SPIR, and presaturation was turned off. The acquisition was performed in a head-first supine position using a multi-dynamic IVIM scheme (15 b-values × 52 slices), with Cartesian reconstruction.

#### 2.2.3 Structural imaging

For image registration and brain tissue segmentation, a sagittal structural three-dimensional (3D) T1-weighted (T1W) image was acquired with the fast field-echo (FFE) sequence. The imaging parameters were as follows: TR = 8.1 ms, TE = 3.7 ms, flip angle (FA) = 8°, and voxel size = 1 × 1 × 1 mm^3^. In addition, T2-weighted turbo-spin-echo, fluid-attenuated inversion recovery (FLAIR), and gradient-echo images were also acquired to evaluate any brain abnormalities. MRI was performed using a 3.0 Tesla MRI system equipped with a 32-channel sensitivity encoding head coil (Ingenia, Philips Medical System, Best, the Netherlands).

### 2.3 Reconstruction of MRI data

#### 2.3.1 ISF mapping using PINN

To estimate IVIM-derived diffusion components related to ISF, we implemented an unsupervised physics-informed neural network (PINN) based on a three-component IVIM (3C-IVIM) signal model proposed by Voorter et al. (2023). The normalized signal decay was modeled as shown in Equation 1:


$$\begin{array}{l} S\left(b\right)/s_{0}=f_{par}\exp\left(-bD_{PAR}\right)+f_{isf}\exp\left(-bD_{ISF}\right)+\\ f_{mv}\exp\left(-bD_{MV}\right) \end{array}$$ (1)

Where the three diffusion parameters are $D_{PAR}\;$ (parenchymal diffusion), $D_{ISF}$ (an intermediate diffusion component interpreted as ISF-related diffusion), and $D_{MV}$ (a microvascular pseudo-diffusion component). In addition, the three corresponding volume fractions are *F_PAR_, F_ISF_*, and F^MV^ with the following relationship: $f_{PAR}=1-f_{ISF}-f_{MV}$ . Finally, S0 is the non-diffusion-weighted signal at b = 0 s/mm^2^. For each subject, T1-weighted images were segmented using FreeSurfer to obtain tissue masks. The gray- and white-matter masks were combined and registered to the IVIM space, and only voxels within the registered tissue mask were used for model training to exclude non-parenchymal regions.

Multi-b-value IVIM images were averaged for each b-value, and voxel-wise signal curves were extracted from the masked region at b = 0, 5, 10, 15, 20, 30, 40, 50, 60, 100, 200, 500, 700, 1,000, and 1,500 s/mm^2^. Each curve was normalized by the unweighted signal $(S_{0},$ b = 0) to define the network input and target ( S(b) / $S_{0}$ ). To minimize the impact of extreme outliers, signal curves showing non-physical values or significant violations of monotonic decay were excluded using a quality filter. However, to avoid biased sampling, this filter was bypassed if the number of retained voxels was insufficient for robust network training. No separate automated eddy current correction or rigid-body motion correction was applied to the DWI-EPI data before PINN fitting; this point is addressed in the limitations section. In addition, no field map-based distortion correction or reverse phase-encoding distortion correction was applied to the DWI-EPI data. As previously demonstrated by Voorter et al. (2023), this PINN-based 3C-IVIM approach provides more robust and repeatable ISF-related parameter maps compared to conventional fitting approaches.

#### 2.3.2 HFC mapping from MREPT data

High-frequency conductivity ( $\sigma_{H}$ ) mapping using MREPT is based on the electromagnetic relationship between the transmit RF magnetic field and the electrical properties of biological tissue (Katscher et al., 2022). The spatial behavior of the RF magnetic field $B_{1}$ in conductive media follows the Helmholtz (Equation 2).


$$\nabla^{2}B_{1}=i\omega \mu_{0}\tau_{H}B_{1}-\frac{\nabla \tau_{H}}{\tau_{H}}\times \left(\nabla \times B_{1}\right)$$ (2)

where *ω* denotes the Larmor angular frequency, $\mu_{0}=4\pi \times 10^{-7}N/A^{2}\;$ is the magnetic permeability of the free space, and $\tau_{H}=\sigma_{H}+i\omega \in_{H}$ represents the complex admittivity composed of high-frequency conductivity *(* $\sigma_{H}$ *)* and permittivity $\varepsilon_{H}$ (Voigt et al., 2011). The transverse RF field of $B_{1}$ can be decomposed into the positively and negatively rotating components, $B_{1}^{+}=\frac{1}{2}\left(B_{x}+iB_{y}\right)$ and $B_{1}^{-}=\frac{1}{2}\left(B_{x}-iB_{y}\right).$

In this study, a multi-channel transmit and receive RF system was used, enabling spatially resolved characterization of the transmit field distribution. However, direct measurement of the absolute transmit field phase is not available in standard clinical acquisitions. A phase-based MREPT was employed, where conductivity is reconstructed from spatial variations in the RF phase rather than from magnitude information (Katscher et al., 2009). MRI provides access to the transceive phase, representing the combined phase contribution of transmit and receive fields, expressed as $\phi^{tr}=\phi^{+}+\phi^{-}$ . Under the transceive phase approximation and assuming local symmetry between transmit and receive sensitivities across channels, the transmit phase can be estimated as $\phi^{+}$ = $\phi^{tr}/2$ , providing a practical and stable estimate of the transmit phase required for conductivity reconstruction (Voigt et al., 2011). Substitution into the Helmholtz formulation yields the phase-based conductivity (Equation 3).


$$\left(\nabla \phi^{tr}\cdot \nabla \left(\frac{1}{\sigma_{H}}\right)\right)+\frac{\nabla^{2}\phi^{tr}}{\sigma_{H}}-2\omega \mu_{0}=0$$ (3)

Where $\phi^{tr}$ represents the transceive phase. Under the local homogeneity assumption, the spatial gradient term becomes negligible, and conductivity can be estimated directly from the Laplacian of the transmit phase (Equation 4).


$$\sigma_{H}=\frac{\nabla^{2}\phi^{tr}}{\omega \mu_{0}}\;$$ (4)

Where $\nabla^{2}$ represents the Laplacian operator and $\mu_{0}$ is the magnetic permeability of free space, and $\phi^{tr}$ is the transceive phase.

After data acquisition, all images were transferred to an independent workstation for analysis using manufacturer-supplied software (Extended Philips Research Software Solution, EXPRESS, Philips Healthcare). The Laplacian of the transceive phase was computed using a locally adaptive second-order polynomial fitting approach, in which second spatial derivatives at each voxel were estimated by fitting a local parabola within a 3D kernel centered at the target voxel. A maximum half-kernel size of $N_{\max}$ = 10 voxels per spatial direction was used and adaptively reduced when the magnitude signal of a candidate kernel voxel differed by more than ± 10 % from that of the target voxel. This magnitude-constrained adaptive kernel preserves tissue boundaries, maintains local homogeneity within the fitting region, and reduces boundary-related artifacts commonly associated with Laplacian-based MREPT reconstruction (Katscher et al., 2012). Numerical evaluation of the Laplacian amplifies high-frequency noise; therefore, spatial regularization of the reconstructed conductivity maps was performed using a magnitude-constrained bilateral median filtering strategy with the same adaptive kernel definition. Voxels contributing to the filtering were restricted to the local neighborhood and to those with magnitude similarity to the target voxel, ensuring edge preservation while suppressing noise-induced fluctuations. Similar locally adaptive Laplacian-based reconstruction strategies have been implemented in various MREPT and electrical properties imaging frameworks, providing stable conductivity estimation across different anatomical applications (Kim et al., 2016; Park et al., 2021).

#### 2.3.3 Post-processing of all reconstructed maps

To process the reconstructed maps of each participant, the Statistical Parametric Mapping version 12 (SPM12) software^1^ was used, and the following post-processing steps were performed. First, all reconstructed maps for each participant were co-registered to the 3D T1W image. Second, the 3D T1W image was spatially normalized into an AD-specific brain template (Guo et al., 2021) using the computational anatomy toolbox (CAT12) tool^2^. Third, all maps were spatially normalized into the brain template using the deformation field information from the 3D T1W image. Finally, Gaussian smoothing with a full width at half maximum (FWHM) of 10 × 10 × 10 mm^3^ was applied to all parameter maps for voxel-based statistical analyses to improve the signal-to-noise ratio, reduce residual inter-subject anatomical variability after spatial normalization, and maintain a consistent preprocessing framework across the reconstructed parameter maps.

The atlas-based region-of-interest (ROI) areas were defined at the corpus callosum, which is the main white matter region in the brain, the hippocampus and middle temporal gyrus (MTG) areas based on knowledge of the affected locations in patients with AD, the insula based on our previous findings, and the cuneus, parahippocampal gyrus, precuneus, putamen, and thalamus based on the results of the voxel-based analyses. The atlas-based ROIs were defined using the wfu_pickatlas software^3^. The mean values of each map and each participant from the selected ROIs were extracted with Marsbar software (Matthew Brett^4^).

### 2.4 Statistical analysis

Because age differed significantly between AD and the other groups, and sex differed significantly between MCI and AD, we always used age and sex as confounding variables in all subsequent statistical analyses.

#### 2.4.1 Demographic data

Age and MMSE scores were compared among the three participant groups using a one-way analysis of variance (ANOVA). When significant differences were observed, post-hoc comparisons were conducted using the Bonferroni test. Sex was compared using the chi-squared test.

#### 2.4.2 Voxel-based analyses

Each parameter map was compared among the three participant groups using voxel-wise full factorial design of one-way analysis of covariance (ANCOVA) with age and sex as covariates. Furthermore, the association between the voxel value of each map and either age or the K-MMSE score, with age and sex as covariates, was evaluated using voxel-based multiple regression analysis. For the voxel-based analyses, a significance level of *α* = 0.05 was applied, with correction for multiple comparisons using the false-discovery rate (FDR) method and clusters with at least 100 contiguous voxels.

#### 2.4.3 Region-of-interest (ROI)-based analyses

For ROI data, α < 0.05 was used to determine the significance level. The statistical analysis was performed using the MedCalc (MedCalc Software, Acacialaan, Ostend, Belgium) statistical program.

#### 2.4.4 Group comparison

A group comparison of the ROI value was performed using a heteroscedasticity-robust ANCOVA (HC3) with age and sex as covariates to evaluate the differences in ROI values between the three participant groups, followed by post-hoc tests with Benjamini-Hochberg (BH) correction for multiple comparisons.

#### 2.4.5 Correlation analyses

The partial correlation coefficient test for each MRI measure was performed to analyze the degree of correlation between the ROI values and the participant’s age and sex as covariates, using all data. In addition, another partial correlation analysis for each MRI measure was conducted between the ROI values and MMSE scores with adjustments for the participants’ age and sex using all data. Furthermore, to investigate the relationship between MREPT-derived HFC and IVIM-derived ISF-related indices (D_ISF_, F_ISF_) in the brain, a partial correlation analysis was performed between HFC and D_ISF_ or F_ISF_ at each ROI, adjusting simultaneously for diagnostic group, age, and sex. Benjamini–Hochberg FDR correction was applied separately for the HFC–D_ISF_ and HFC–F_ISF_ association families across bilateral ROI tests.

#### 2.4.6 Multiple-regression analyses

To evaluate any associations between HFC and IVIM diffusion indices, a robust multiple regression analysis was performed between HFC and IVIM diffusion indices with age and sex as covariates using the following model:


$$\begin{array}{l} \mathrm{HFC}\approx \beta_{1}*\mathrm{Age}+\beta_{2}*\mathrm{Sex}+\beta_{3}*D_{\mathrm{ISF}}+\\ \beta_{4}*D_{\mathrm{MV}}+\beta_{5}*D_{\mathrm{PAR}}+\mathrm{error}\; \end{array}$$ (5)

In addition, this multiple regression analysis was repeated using the following model to evaluate any associations between HFC and IVIM fraction indices:


$$\begin{array}{l} \mathrm{HFC}\approx \beta_{1}*\mathrm{Age}+\beta_{2}*\mathrm{Sex}+\beta_{3}*F_{\mathrm{ISF}}+\\ \beta_{4}*F_{\mathrm{MV}}+\beta_{5}*F_{\mathrm{PAR}}+error \end{array}$$ (6)

#### 2.4.7 Receiver operating characteristic (ROC) curve analysis

Finally, an ROC curve analysis was conducted for each MRI index across all ROIs to assess the differentiation between participant groups. The optimal cut-off point, which maximizes the combined measure of sensitivity and specificity, was identified using the Youden Index. We report sensitivity (SE), specificity (SP), and area under the curve (AUC), along with the corresponding *p* value. This analysis was performed on the same dataset used for group comparisons and should be interpreted as exploratory and hypothesis-generating. Reported AUC values may be optimistic and should be viewed as within-sample performance estimates in the absence of independent validation.

## 3 Results

Age differed significantly across groups (ANOVA, *F* = 17, *p* < 0.001), with the AD group being older than the CN and aMCI groups. MMSE scores also differed significantly across groups (ANOVA, *F* = 130, *p* < 0.001), with significant *post hoc* differences across all pairwise comparisons. Sex distribution differed significantly between the aMCI and AD groups (χ^2^ = 7.33, *p* = 0.007), whereas the other pairwise comparisons were not significant. Table 1 summarizes the demographic characteristics and neuropsychological test results of the participant groups.

**Table 1:** Summary of the demographic data.

Parameters	CN (0)	aMCI (1)	AD (2)	Statistics
*N*	26	78	57	N/A
Age	72.42 ± 6.81	73.94 ± 7.65	80.09 ± 5.72	***F = 17, p < 0.001*** ***(0, 2), (1, 2)***
Sex (Male/Female)	11 (42.3%)/15 (57.7%)	37 (47.4%)/41 (52.6%)	14 (24.6%)/43 (75.4%)	(0,1) χ^2^ = 0.21, *p* = 0.650 (0,2) χ^2^ = 2.67, *p* = 0.102 ***(1,2) χ***^***2***^ ***= 7.33, p = 0.007***
MMSE	28.115 ± 1.366	26.295 ± 2.245	17.439 ± 5.251	***F = 130, p < 0.001*** ***(0, 1), (0, 2), (1, 2)***
CDR	0.0: 18 (69.2%) 0.5: 8 (30.8%)	0.5: 78 (100.0%)	0.5: 7 (12.3%) 1.0: 40 (70.2%) 2.0: 10 (17.5%)	N/A
(Attention) Digit Span Backward	−0.07 ± 1.07	−0.27 ± 1.06	−0.80 ± 1.03	*F* = 5.105, *p* = 0.007 (0,2), (1,2)
(Language) K-BNT	0.23 ± 0.72	−0.70 ± 1.19	−1.53 ± 1.51	*F* = 17.158, *p* < 0.001 (0,1), (0,2), (1,2)
(Visuospatial) Rey-CFT Copy	0.16 ± 0.55	−0.54 ± 1.43	−2.09 ± 2.16	*F* = 20.320, *p* < 0.001 (0,2), (1,2)
(Memory) SVLT Delayed Recall	0.37 ± 0.77	−1.45 ± 0.98	−1.77 ± 0.69	*F* = 55.398, *p* < 0.001 (0,1), (0,2)
K-TMT-B	0.07 ± 1.24	−0.56 ± 1.62	−2.87 ± 2.47	*F* = 26.909, *p* < 0.001 (0,2), (1,2)
Stroop (Color Reading)	0.42 ± 0.76	−0.44 ± 1.19	−1.53 ± 1.57	*F* = 20.423, *p* < 0.001 (0,1), (0,2), (1,2)
COWAT (Phonemic)	0.21 ± 0.58	−0.36 ± 1.03	−1.01 ± 1.03	*F* = 13.454, *p* < 0.001 (0,1), (0,2), (1,2)

Continuous variables are presented as mean ± standard deviation.

For between-group comparisons, age and Mini-Mental State Examination (MMSE) scores were analyzed using one-way ANOVA (F and *p* values). When the overall ANOVA was significant, post hoc pairwise comparisons were performed using two-tailed independent-samples t-tests with Bonferroni correction (*α* = 0.05). Significant pairwise differences are indicated as (0,1) for CN vs. aMCI, (0,2) for CN vs. AD, and (1,2) for aMCI vs. AD.

Sex distribution was assessed using pairwise chi-squared tests (CN vs. aMCI, CN vs. AD, and aMCI vs. AD), and *p* values were Bonferroni-corrected for the three comparisons (*α* = 0.05).

CN, cognitively normal; aMCI, amnestic mild cognitive impairment; AD, Alzheimer’s disease; CDR, clinical dementia rating.

**Figure 1:**
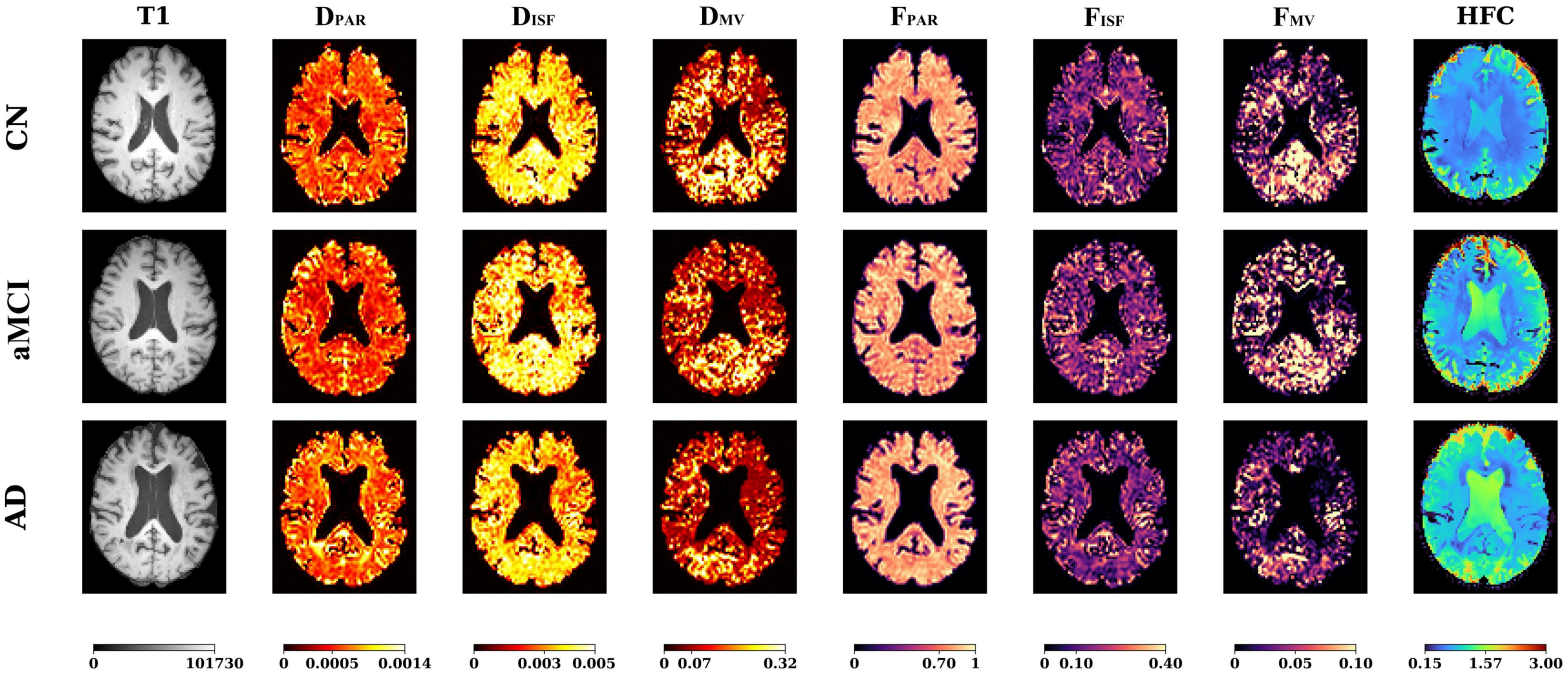
Representative maps of IVIM parameters (D_PAR_, D_ISF_, D_MV_, F_PAR_, F_ISF_, and F_MV_) and high-frequency conductivity (HFC) obtained from one cognitively normal (CN), aMCI, and Alzheimer’s disease (AD) participants. IVIM, intravoxel incoherent motion; D_PAR_, parenchymal diffusion; D_ISF_, an intermediate diffusion component interpreted as ISF-related diffusion; D_MV_, a microvascular pseudo-diffusion component; F_PAR_, parenchymal volume fractions; F_ISF_, ISF volume fractions; F_MV_, microvascular volume fractions.

Figure 1 shows the representative T1-weighted images, the IVIM maps of three diffusion parameters (D_ISF_, D_PAR_, and D_MV_) and three volume fractions (F_ISF_, F_PAR_, and F_MV_), and HFC obtained from the brain of one CN participant (a 69-year-old male), one MCI patient (a 74-year-old female), and one AD patient (a 75-year-old female). Visually, these representative maps demonstrate distinct alterations consistent with disease progression. Compared to the CN participant, the AD patient exhibits a general trend of reduced signal intensities in D_ISF_, D_MV_, and F_PAR_ (appearing relatively darker), accompanied by increased intensities in F_ISF_ and HFC (EPT) (appearing brighter or shifting to green/yellow hues), which qualitatively support the statistical group differences.

### 3.1 Voxel-based analyses

#### 3.1.1 Group comparison

Supplementary Figure S3 shows the results of the voxel-based group comparison among the three participant groups of MRI maps. For diffusion parameters from IVIM, D_ISF_ was higher in CN than in MCI and AD. D_MV_ was higher in CN than in MCI and AD, and higher in MCI than in AD. D_PAR_ was lower in CN than in AD. For volume fraction parameters from IVIM, F_ISF_ was lower in CN and MCI than in AD. F_PAR_ was higher in CN than in MCI and AD, and higher in MCI than in AD. For the MREPT parameter, HFC was lower in CN and MCI than in AD. The detailed locations of the significant difference for the voxel-based ANOVA test for each map are listed in Supplementary Table S1. A supplementary sensitivity analysis with explicit CSF masking (Supplementary Figure S3D) revealed that the voxel-wise group differences in HFC did not survive multiple comparisons correction after CSF exclusion, indicating that the bulk HFC alterations observed in the unmasked group comparison are closely coupled with the expansion of CSF and extracellular fluid compartments in atrophied AD brains.

#### 3.1.2 Association between MRI map and age or MMSE scores

Age was negatively associated with D_ISF_ and D_MV_ but positively associated with D_PAR_ (Supplementary Figure S4). Additionally, age was positively associated with F_ISF_ and F_MV_, while being negatively associated with F_PAR_. Furthermore, age showed a positive association with HFC; notably, this HFC–age association remained significant in specific regions even after explicit CSF masking (Supplementary Figure S4D). This suggests that age-related increases in HFC are at least partly independent of CSF partial-volume effects. The detailed locations of the significantly associated areas are listed in Supplementary Table S2. The MMSE score was not significantly associated with any MRI parameter maps in the voxel-based analysis. This finding does not necessarily conflict with the ROI-based results, as voxel-based and ROI-based analyses differ in spatial sensitivity and statistical thresholding; this point is discussed further in Section 4.3.

### 3.2 ROI-based analyses

#### 3.2.1 Group comparison of MRI measures

The mean values with standard deviation for each MRI parameter at each ROI are listed in Supplementary Table S3, which includes the primary ANCOVA F-statistics, HC3-based heteroscedasticity-robust *p* values, and Benjamini–Hochberg FDR-adjusted *p* values. D_ISF_ was significantly lower in AD than in CN or MCI in the bilateral hippocampi, right insula, right middle temporal gyrus, and bilateral parahippocampal gyri. D_MV_ was significantly lower in AD than in CN or MCI in the corpus callosum, bilateral hippocampi, right insula, right middle temporal gyrus, and bilateral parahippocampal gyri. D_PAR_ was significantly lower in AD than in CN in the right hippocampus. F_ISF_ was significantly higher in AD than in CN in the right middle temporal gyrus and the bilateral putamen. F_MV_ was significantly lower in AD than in CN in the left thalamus. F_PAR_ was significantly lower in AD than in CN or MCI in the right corpus callosum, bilateral hippocampi, right insula, right middle temporal gyrus, and bilateral parahippocampal gyri. Finally, HFC was significantly higher in AD than in CN in the right thalamus.

#### 3.2.2 Correlation between MRI measure and age

**Figure 2:**
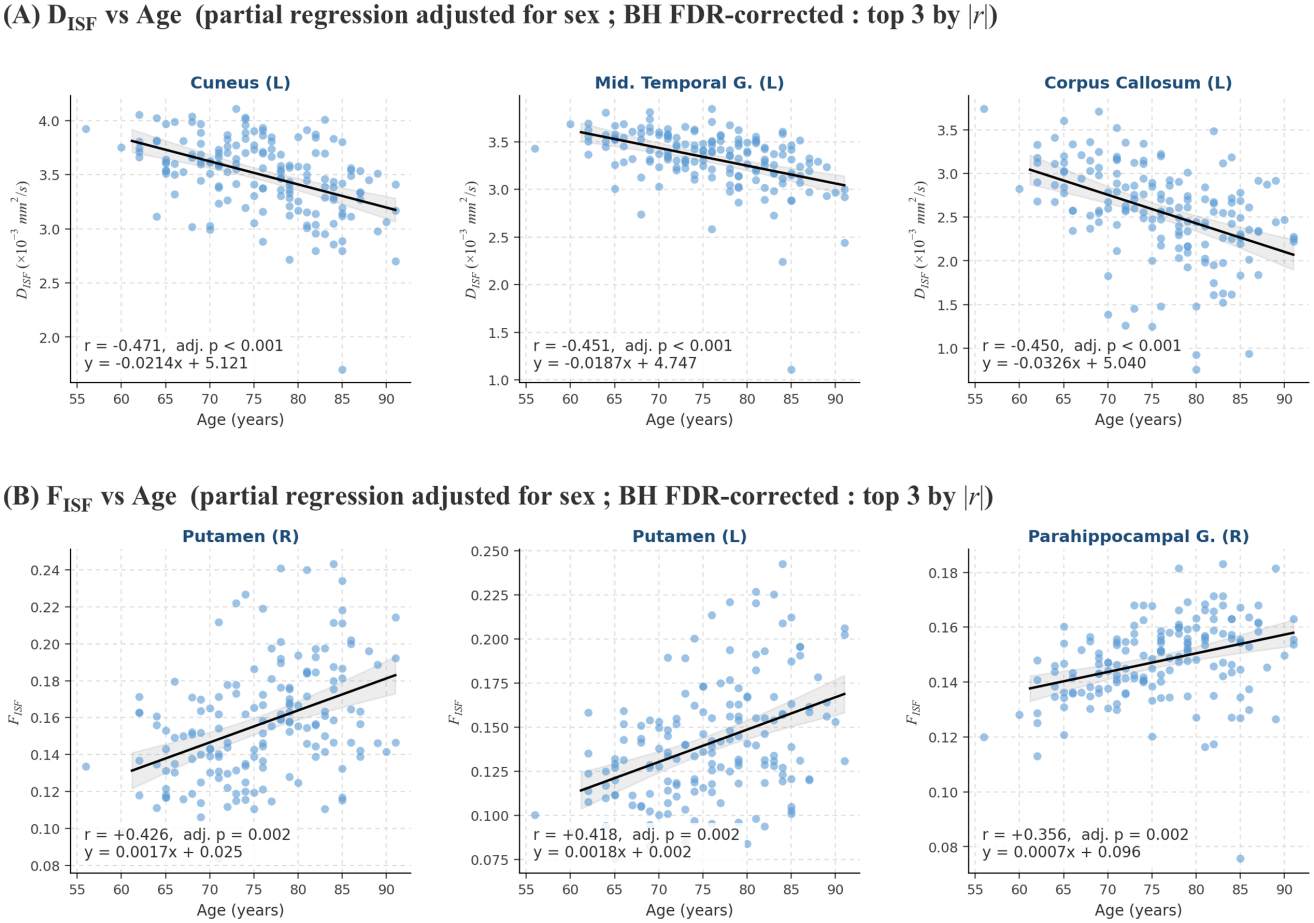
Correlation between interstitial fluid (ISF) measures and age in selected regions of interest (ROIs). Scatter plots illustrating the linear relationship of D_ISF_ **(A)** and F_ISF_ **(B)** with age. The specific ROIs presented in these panels were selected to highlight the regions with the most significant correlation coefficients (*γ* values) among all defined ROIs. Solid lines represent the fitted linear regression lines for each specific ROI, demonstrating the overall trend of the parameters across ages.

Figure 2 shows the results of the correlation analysis between ISF parameters and age. In addition, correlation coefficients and the corresponding *p* values are listed in Supplementary Table S4. D_ISF_ and D_MV_ were significantly negatively correlated with age at most of the defined ROIs (FDR-adjusted *p* < 0.05). D_PAR_ showed significant positive correlations with age in select ROIs, including the bilateral hippocampus, bilateral precuneus, and bilateral putamen. F_ISF_ was significantly positively correlated with age in select ROIs, including the bilateral parahippocampal gyrus, bilateral precuneus, bilateral putamen, and bilateral thalamus. F_MV_ was significantly negatively correlated with age in select ROIs including the bilateral corpus callosum, left hippocampus, right putamen, and bilateral thalamus. F_PAR_ was significantly negatively correlated with age at most of the defined ROIs. Finally, HFC was significantly positively correlated with age in most of the defined ROIs, including the bilateral corpus callosum, bilateral insula, bilateral parahippocampal gyrus, bilateral putamen, and bilateral thalamus.

#### 3.2.3 Correlation between MRI measure and MMSE scores

**Figure 3:**
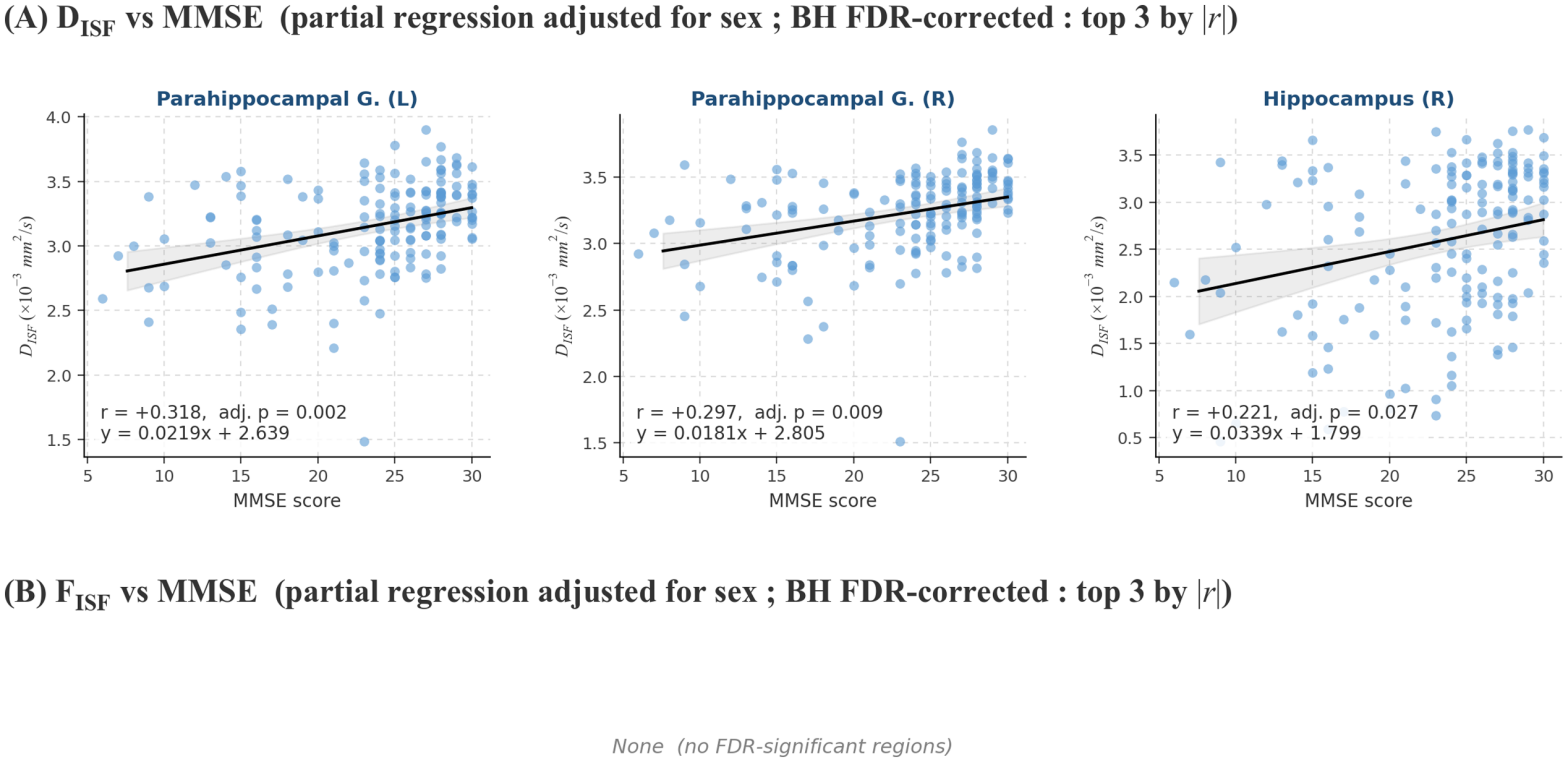
Correlation between interstitial fluid (ISF) measures and Mini-Mental State Examination (MMSE) scores in selected regions of interest (ROIs). Scatter plots illustrating the linear relationship of D_ISF_ **(A)** and F_ISF_ **(B)** with MMSE scores. The specific ROIs presented in these panels were selected to highlight the regions with the most significant correlation coefficients (γ values) among all defined ROIs. Solid lines represent the fitted linear regression lines for each ROI, illustrating the overall trend in ISF parameters associated with cognitive function.

Figure 3 shows the results of the correlation analysis between ISF parameters and MMSE scores. In addition, correlation coefficients and the corresponding *p* values are listed in Supplementary Table S4. After Benjamini–Hochberg FDR correction, D_ISF_ was significantly positively correlated with MMSE scores in the bilateral hippocampus, right insula, and bilateral parahippocampal gyrus (FDR-adjusted *p* < 0.05). D_MV_ was significantly positively correlated with MMSE scores in the bilateral hippocampus, right insula, bilateral parahippocampal gyrus, and left precuneus. F_PAR_ was significantly positively correlated with MMSE scores in the bilateral hippocampus and bilateral parahippocampal gyrus. D_PAR_, F_ISF_, F_MV_, and HFC did not show significant associations with MMSE scores after FDR correction in any of the defined ROIs.

#### 3.2.4 Correlation between HFC and diffusion or fractional ISF-related measures (D_ISF_, F_ISF_)

**Figure 4:**
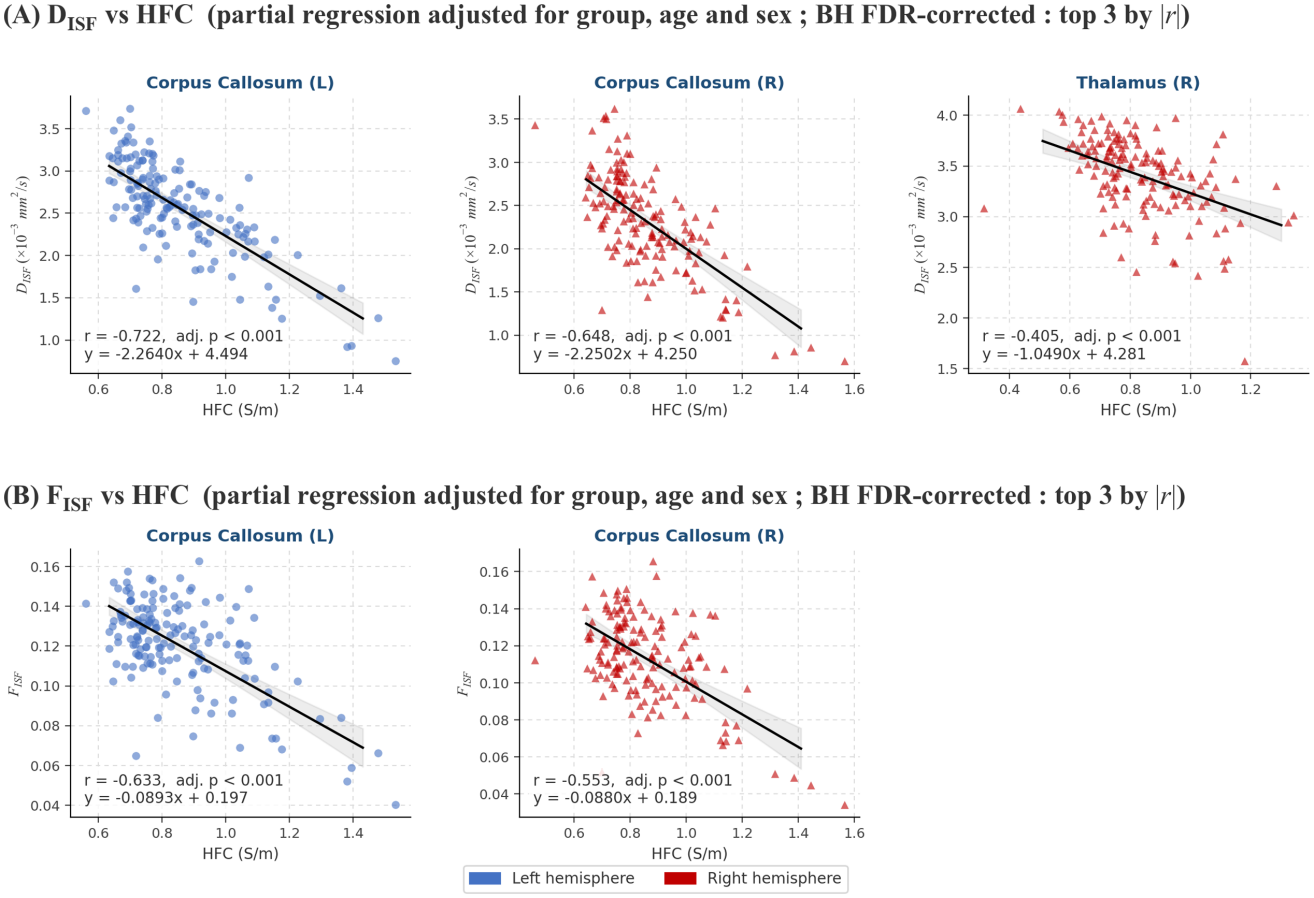
Correlation between high-frequency conductivity (HFC) and interstitial fluid (ISF) measures of D_ISF_ **(A)** or F_ISF_ **(B)** in the representative ROIs. The solid lines represent the fitted linear regression lines for each specific ROI.

Figure 4 shows the results of the partial correlation analysis between MREPT-derived HFC and IVIM-derived diffusion or fractional ISF-related measures (D_ISF_, F_ISF_), adjusting for diagnostic group, age, and sex. The partial correlation coefficients and BH FDR-adjusted *p* values are summarized in Supplementary Table S5. After adjustment, HFC was significantly negatively correlated with D_ISF_ in the bilateral corpus callosum (*r* = −0.722 and −0.648, FDR-adjusted *p* < 0.001), left parahippocampal gyrus (*r* = −0.199, FDR-adjusted *p* = 0.046), and bilateral thalamus (*r* = −0.350 and −0.405, FDR-adjusted *p* < 0.001). HFC was also significantly negatively correlated with F_ISF_ in the bilateral corpus callosum (*r* = −0.633 and −0.553, FDR-adjusted *p* < 0.001). No other ROIs showed significant HFC–D_ISF_ or HFC–F_ISF_ associations after FDR correction.

Group-stratified analyses and formal interaction testing are summarized in Supplementary Table S6. Overall, the HFC–ISF associations did not show consistent heterogeneity across diagnostic groups after FDR correction. The only nominal interaction was observed for HFC versus D_ISF_ in the right putamen (interaction *p* = 0.024), but this did not survive BH correction (adjusted interaction *p* = 0.397).

#### 3.2.5 Multiple regression analysis

Table 2 lists the results of the robust multiple regression analyses between HFC and diffusion parameters from IVIM using Equation 5. HFC was significantly negatively associated with D_ISF_ in the left thalamus (*β* = −0.660, FDR-adjusted *p* < 0.001), with nominally significant associations in the right parahippocampal gyrus and right thalamus (FDR-adjusted *p* > 0.05). HFC was not significantly associated with D_MV_ in any defined ROI after FDR correction. Overall, the independent associations between HFC and D_ISF_ were limited to select thalamic and parahippocampal regions.

**Table 2:** Result of the robust multiple regression analysis between high-frequency conductivity (HFC) value and diffusion-related MRI indices.

ROIs	Side	Independent variables					
		Age	Sex	D_ISF_	D_MV_	DP_AR_	Model
Corpus callosum	Lt	***β* = 0.141, *p* = 0.027**	*β* = −0.072, *p* = 0.139	*β* = −0.317, *p* = 0.076 Adj *p* = 0.319	*β* = −0.266, *p* = 0.094 Adj *p* = 0.484	*β* = −0.179, *p* = 0.059 Adj *p* = 0.210	*F* = 55.540, *p* = <0.001*
	Rt	***β* = 0.184, *p* = 0.010**	*β* = −0.001, *p* = 0.982	*β* = −0.171, *p* = 0.417 Adj *p* = 0.683	*β* = −0.328, *p* = 0.071 Adj *p* = 0.484	*β* = −0.201, *p* = 0.070 Adj *p* = 0.210	*F* = 40.438, *p* = <0.001*
Cuneus	Lt	*β* = −0.116, *p* = 0.214	*β* = −0.019, *p* = 0.822	*β* = −0.041, *p* = 0.830 Adj *p* = 0.933	*β* = −0.021, *p* = 0.887 Adj *p* = 0.940	*β* = 0.215, *p* = 0.051 Adj *p* = 0.210	*F* = 1.565, *p* = 0.173
	Rt	*β* = −0.135, *p* = 0.136	*β* = −0.087, *p* = 0.308	*β* = −0.260, *p* = 0.222 Adj *p* = 0.572	*β* = 0.060, *p* = 0.709 Adj *p* = 0.885	*β* = 0.236, *p* = 0.053 Adj *p* = 0.210	*F* = 1.495, *p* = 0.195
Hippocampus	Lt	***β* = 0.253, *p* = 0.005**	*β* = 0.071, *p* = 0.384	*β* = 0.234, *p* = 0.417 Adj *p* = 0.683	*β* = −0.118, *p* = 0.555 Adj *p* = 0.769	*β* = −0.040, *p* = 0.837 Adj *p* = 0.886	*F* = 2.175, *p* = 0.060
	Rt	*β* = 0.037, *p* = 0.702	*β* = 0.026, *p* = 0.749	*β* = −0.107, *p* = 0.755 Adj *p* = 0.933	*β* = −0.071, *p* = 0.752 Adj *p* = 0.885	*β* = 0.116, *p* = 0.636 Adj *p* = 0.818	*F* = 0.385, *p* = 0.859
Insula	Lt	*β* = 0.478, *p* = <0.001*	*β* = −0.041, *p* = 0.565	*β* = −0.208, *p* = 0.300 Adj *p* = 0.675	*β* = 0.042, *p* = 0.787 Adj *p* = 0.885	*β* = 0.105, *p* = 0.363 Adj *p* = 0.654	*F* = 11.941, *p* = <0.001*
	Rt	*β* = 0.467, *p* = <0.001*	*β* = 0.027, *p* = 0.719	*β* = 0.070, *p* = 0.750 Adj *p* = 0.933	*β* = −0.222, *p* = 0.178 Adj *p* = 0.641	*β* = −0.073, *p* = 0.562 Adj *p* = 0.778	*F* = 13.981, *p* = <0.001*
Middle Temporal Gyrus	Lt	*β* = 0.188, *p* = 0.052	*β* = −0.065, *p* = 0.429	*β* = 0.230, *p* = 0.199 Adj *p* = 0.572	*β* = −0.125, *p* = 0.386 Adj *p* = 0.695	*β* = −0.040, *p* = 0.728 Adj *p* = 0.820	*F* = 1.069, *p* = 0.380
	Rt	*β* = 0.166, *p* = 0.084	*β* = −0.142, *p* = 0.078	*β* = 0.012, *p* = 0.958 Adj *p* = 0.995	*β* = 0.003, *p* = 0.984 Adj *p* = 0.984	*β* = 0.117, *p* = 0.459 Adj *p* = 0.688	*F* = 1.821, *p* = 0.112
Parahippocampal Gyrus	Lt	***β* = 0.254, *p* = 0.004**	*β* = 0.119, *p* = 0.116	*β* = −0.084, *p* = 0.733 Adj *p* = 0.933	*β* = −0.240, *p* = 0.239 Adj *p* = 0.695	*β* = 0.101, *p* = 0.441 Adj *p* = 0.688	*F* = 7.585, *p* = <0.001*
	Rt	***β* = 0.186, *p* = 0.043**	*β* = 0.027, *p* = 0.727	***β* = −0.596, *p* = 0.008** Adj *p* = 0.072	*β* = 0.351, *p* = 0.062 Adj *p* = 0.484	*β* = 0.149, *p* = 0.215 Adj *p* = 0.485	***F* = 4.052, *p* = 0.002**
Precuneus	Lt	*β* = 0.140, *p* = 0.122	*β* = −0.042, *p* = 0.621	*β* = 0.029, *p* = 0.822 Adj *p* = 0.933	*β* = −0.064, *p* = 0.545 Adj *p* = 0.769	*β* = −0.002, *p* = 0.989 Adj *p* = 0.989	*F* = 0.830, *p* = 0.530
	Rt	*β* = 0.046, *p* = 0.597	*β* = −0.021, *p* = 0.802	*β* = −0.107, *p* = 0.398 Adj *p* = 0.683	*β* = −0.093, *p* = 0.382 Adj *p* = 0.695	*β* = 0.037, *p* = 0.707 Adj *p* = 0.820	*F* = 1.205, *p* = 0.310
Putamen	Lt	*β* = 0.360, *p* = <0.001*	*β* = −0.079, *p* = 0.299	*β* = 0.208, *p* = 0.089 Adj *p* = 0.319	*β* = −0.116, *p* = 0.350 Adj *p* = 0.695	***β* = −0.198, *p* = 0.023** Adj *p* = 0.210	***F* = 4.565, *p* = <0.001**
	Rt	*β* = 0.409, *p* = <0.001*	*β* = −0.105, *p* = 0.181	*β* = −0.001, *p* = 0.995 Adj *p* = 0.995	*β* = −0.083, *p* = 0.508 Adj *p* = 0.769	*β* = −0.140, *p* = 0.138 Adj *p* = 0.354	*F* = 6.028, *p* = <0.001*
Thalamus	Lt	***β* = 0.251, *p* = 0.004**	***β* = −0.138, *p* = 0.040**	*β* = −0.660, *p* = <0.001* **Adj *p* = <0.001**	*β* = 0.208, *p* = 0.107 Adj *p* = 0.484	***β* = 0.223, *p* = 0.031** Adj *p* = 0.210	*F* = 15.927, *p* = <0.001*
	Rt	***β* = 0.245, *p* = 0.003**	***β* = −0.141, *p* = 0.035**	***β* = −0.365, *p* = 0.012** Adj *p* = 0.074	*β* = −0.119, *p* = 0.368 Adj *p* = 0.695	*β* = 0.107, *p* = 0.255 Adj *p* = 0.511	*F* = 20.528, *p* = <0.001*

$HFC\approx \beta_{1}*Age+\beta_{2}*SEX+\beta_{3}*D_{ISF}+\beta_{4}*D_{MV}+\beta_{5}*D_{PAR}+error.$

Adjusted *p* values were calculated using the Benjamini–Hochberg procedure across bilateral ROI tests for each primary MRI predictor separately. Multiplicity correction was applied to the *p* values for D_ISF_, D_MV_, and D_PAR_ only.

HFC, high-frequency conductivity; ROI, region of interest; Lt, left; Rt, right; IVIM, intravoxel incoherent motion; D_PAR_, parenchymal diffusion; D_ISF_, an intermediate diffusion component interpreted as ISF-related diffusion; D_MV_, a microvascular pseudo-diffusion component; *β*, regression coefficient; F, F-statistic; *p*, probability value.

Bold values indicate statistical significance at *p* < 0.05. For the primary MRI predictors, bold adjusted *p* values indicate significance after Benjamini–Hochberg correction.

Table 3 lists the results of the robust multiple regression analyses between HFC and volume fraction parameters from IVIM using Equation 6. HFC showed nominal associations with F_ISF_ in the left hippocampus (negative) and left insula (positive), but neither association survived BH correction, indicating directionally inconsistent region-specific effects. HFC showed significant associations with F_MV_ in select ROIs and was significantly negatively associated with F_PAR_ in most of the defined ROIs, representing the most consistent independent association in Table 3.

**Table 3:** Result of the robust multiple regression analysis between high-frequency conductivity (HFC) value and volume fraction-related MRI indices.

ROIs	Side	Independent variables					
		Age	Sex	F_ISF_	F_MV_	F_PAR_	Model
Corpus callosum	Lt	*β* = 0.017, *p* = 0.783	*β* = −0.044, *p* = 0.348	*β* = −0.011, *p* = 0.910 Adj *p* = 0.928	*β* = 0.002, *p* = 0.977 Adj *p* = 0.977	*β* = −0.807, *p* = <0.001* Adj *p* = <0.001*	*F* = 66.596, *p* = <0.001*
	Rt	*β* = 0.058, *p* = 0.402	*β* = −0.006, *p* = 0.903	*β* = 0.095, *p* = 0.379 Adj *p* = 0.807	*β* = −0.093, *p* = 0.322 Adj *p* = 0.725	*β* = −0.737, *p* = <0.001* Adj *p* = <0.001*	*F* = 44.314, *p* = <0.001*
Cuneus	Lt	*β* = −0.078, *p* = 0.418	*β* = −0.029, *p* = 0.719	*β* = 0.109, *p* = 0.248 Adj *p* = 0.745	*β* = −0.046, *p* = 0.601 Adj *p* = 0.844	*β* = 0.060, *p* = 0.560 Adj *p* = 0.672	*F* = 0.887, *p* = 0.492
	Rt	*β* = −0.059, *p* = 0.545	*β* = −0.120, *p* = 0.143	*β* = 0.012, *p* = 0.904 Adj *p* = 0.928	*β* = −0.119, *p* = 0.183 Adj *p* = 0.548	*β* = 0.074, *p* = 0.495 Adj *p* = 0.636	*F* = 1.154, *p* = 0.335
Hippocampus	Lt	***β* = 0.318, *p* = <0.001**	*β* = 0.081, *p* = 0.294	***β* = −0.298, *p* = 0.024** Adj *p* = 0.213	***β* = 0.320, *p* = 0.011** Adj *p* = 0.097	*β* = 0.130, *p* = 0.204 Adj *p* = 0.367	***F* = 3.672, *p* = 0.004**
	Rt	*β* = 0.069, *p* = 0.476	*β* = 0.021, *p* = 0.795	*β* = 0.037, *p* = 0.822 Adj *p* = 0.928	*β* = −0.067, *p* = 0.610 Adj *p* = 0.844	*β* = −0.022, *p* = 0.865 Adj *p* = 0.916	*F* = 0.310, *p* = 0.906
Insula	Lt	***β* = 0.307, *p* = <0.001**	*β* = −0.022, *p* = 0.744	***β* = 0.237, *p* = 0.022** Adj *p* = 0.213	*β* = −0.077, *p* = 0.410 Adj *p* = 0.737	*β* = −0.335, *p* = <0.001* **Adj *p* = <0.001**	*F* = 16.221, *p* = <0.001*
	Rt	*β* = 0.379, *p* = <0.001*	*β* = 0.025, *p* = 0.743	*β* = 0.102, *p* = 0.363 Adj *p* = 0.807	*β* = −0.070, *p* = 0.492 Adj *p* = 0.806	***β* = −0.277, *p* = 0.005** **Adj *p* = 0.012**	*F* = 14.591, *p* = <0.001*
Middle Temporal Gyrus	Lt	*β* = 0.164, *p* = 0.100	*β* = −0.050, *p* = 0.535	*β* = 0.011, *p* = 0.913 Adj *p* = 0.928	*β* = −0.031, *p* = 0.726 Adj *p* = 0.872	*β* = 0.052, *p* = 0.616 Adj *p* = 0.693	*F* = 0.746, *p* = 0.590
	Rt	*β* = 0.148, *p* = 0.144	*β* = −0.135, *p* = 0.095	*β* = 0.095, *p* = 0.404 Adj *p* = 0.807	*β* = 0.038, *p* = 0.666 Adj *p* = 0.856	*β* = 0.004, *p* = 0.977 Adj *p* = 0.977	*F* = 1.735, *p* = 0.130
Parahippocampal Gyrus	Lt	*β* = 0.191, *p* = 0.063	*β* = 0.114, *p* = 0.120	*β* = 0.181, *p* = 0.161 Adj *p* = 0.724	*β* = −0.216, *p* = 0.082 Adj *p* = 0.296	***β* = −0.239, *p* = 0.017** **Adj *p* = 0.039**	*F* = 8.004, *p* = <0.001*
	Rt	*β* = 0.150, *p* = 0.180	*β* = 0.029, *p* = 0.708	*β* = −0.017, *p* = 0.882 Adj *p* = 0.928	*β* = −0.029, *p* = 0.781 Adj *p* = 0.879	*β* = −0.191, *p* = 0.070 Adj *p* = 0.140	***F* = 3.253, *p* = 0.008**
Precuneus	Lt	*β* = 0.118, *p* = 0.203	*β* = −0.034, *p* = 0.676	*β* = −0.009, *p* = 0.928 Adj *p* = 0.928	*β* = 0.100, *p* = 0.261 Adj *p* = 0.670	*β* = −0.078, *p* = 0.402 Adj *p* = 0.604	*F* = 1.361, *p* = 0.242
	Rt	*β* = −0.045, *p* = 0.626	*β* = −0.006, *p* = 0.943	*β* = 0.153, *p* = 0.099 Adj *p* = 0.594	***β* = −0.206, *p* = 0.016** Adj *p* = 0.097	***β* = −0.319, *p* = 0.001** **Adj *p* = 0.004**	***F* = 2.890, *p* = 0.016**
Putamen	Lt	***β* = 0.265, *p* = 0.002**	*β* = −0.084, *p* = 0.275	*β* = 0.043, *p* = 0.636 Adj *p* = 0.928	***β* = 0.154, *p* = 0.047** Adj *p* = 0.209	*β* = −0.094, *p* = 0.270 Adj *p* = 0.442	***F* = 4.410, *p* = <0.001**
	Rt	*β* = 0.377, *p* = <0.001*	*β* = −0.113, *p* = 0.156	*β* = −0.047, *p* = 0.631 Adj *p* = 0.928	*β* = 0.009, *p* = 0.911 Adj *p* = 0.965	*β* = −0.072, *p* = 0.459 Adj *p* = 0.635	*F* = 5.614, *p* = <0.001*
Thalamus	Lt	*β* = 0.148, *p* = 0.068	*β* = −0.093, *p* = 0.155	*β* = 0.086, *p* = 0.242 Adj *p* = 0.745	*β* = −0.061, *p* = 0.392 Adj *p* = 0.737	*β* = −0.495, *p* = <0.001* Adj *p* = <0.001*	*F* = 20.688, *p* = <0.001*
	Rt	***β* = 0.196, *p* = 0.013**	*β* = −0.125, *p* = 0.055	*β* = 0.018, *p* = 0.793 Adj *p* = 0.928	***β* = −0.217, *p* = 0.001** **Adj *p* = 0.026**	*β* = −0.406, *p* = <0.001* Adj *p* = <0.001*	*F* = 24.945, *p* = <0.001*

$\mathrm{HFC}\approx \beta_{1}*\mathrm{Age}+\beta_{2}*\mathrm{SEX}+\beta_{3}*F_{\mathrm{ISF}}+\beta_{4}*F_{\mathrm{MV}}+\beta_{5}*F_{\mathrm{PAR}}+error.$

Adjusted *p* values were calculated using the Benjamini–Hochberg procedure across bilateral ROI tests for each primary MRI predictor separately. Multiplicity correction was applied to the *p* values for F_ISF_, F_MV_, and F_PAR_ only.

HFC, high-frequency conductivity; ROI, region of interest; Lt, left; Rt, right; IVIM, intravoxel incoherent motion; F_PAR_, parenchymal volume fractions; F_ISF_, ISF volume fractions; F_MV_, microvascular volume fractions; *β*, regression coefficient; F, F-statistic; *p*, probability value.

Bold values indicate statistical significance at *p* < 0.05. For the primary MRI predictors, bold adjusted *p* values indicate significance after Benjamini–Hochberg correction.

#### 3.2.6 ROC curve analysis of MRI indices

The results of the ROC curve analysis of each MRI measure obtained in the specific brain areas are summarized in Supplementary Table S7. In addition, representative ROC curves are also shown in Supplementary Figure S5. In differentiation of AD from CN, F_PAR_ in the right (0.907 with SE = 84 and SP = 81) and left (0.893 with SE = 79 and SP = 88) parahippocampal gyrus had large AUC values. In differentiation of AD from MCI, F_PAR_ in the right (0.797 with SE = 95 and SP = 54) and left (0.787 with SE = 82 and SP = 64) parahippocampal gyrus had large AUC values. In differentiation of MCI from CN, D_ISF_ in the right middle temporal gyrus (SE = 49, SP = 92, AUC = 0.698) and F_PAR_ in the left putamen (SE = 56, SP = 85, AUC = 0.714) were significant, but in general, AUC values were lower than those of the other group comparisons. These results are presented as exploratory findings, as the analyses were performed on the same dataset used for group comparisons; accordingly, the reported AUC values should be interpreted as within-sample performance estimates and may be optimistic with respect to independent datasets.

## 4 Discussion

The primary purpose of this study was to investigate the relationship between MREPT-derived brain HFC and IVIM-derived ISF-related metrics in patients with cognitive impairments across the MCI-AD spectrum. Our study yielded three main findings. First, multiple regression analyses showed limited and region-specific independent associations between selected ISF-related indices and HFC, with the most robust finding observed for D_ISF_ in the left thalamus. Second, HFC exhibited region-specific correlations with ISF-related measures, notably in the bilateral corpus callosum and thalamus. Third, D_ISF_ was significantly associated with both age and MMSE scores, whereas F_ISF_ was significantly associated with age but not with MMSE after FDR correction.

### 4.1 Association between HFC and IVIM indices in specific brain areas

Our first major finding from the multiple regression analyses, adjusting for age and sex, was that selected ISF-related IVIM indices exhibited limited and region-specific independent associations with HFC. Multiple regression analysis was employed to evaluate whether the observed associations between ISF-related measures and HFC persisted after covariate adjustment, moving beyond simple mutual correlation.

As shown in Table 2, HFC was independently and negatively associated with D_ISF_ in the right parahippocampal gyrus and bilateral thalamus, but only the left thalamus remained significant after BH correction, whereas the associations in the right parahippocampal gyrus and right thalamus were nominal. ISF mobility (D_ISF_) represents extracellular ISF-related diffusion in the perivascular and interstitial spaces, and HFC reflects tissue ion mobility and concentration. The negative association between them suggests that restricted ISF mobility may lead to the accumulation of metabolic waste and extracellular ions, such as sodium and potassium. This disruption of ionic homeostasis may consequently elevate the bulk electrical conductivity of the tissue (Vitvitsky et al., 2012; Lee et al., 2021).

Furthermore, as presented in Table 3, although F_ISF_ reached nominal significance in only 2 of 18 ROI-hemisphere combinations, with opposing effect directions across regions and no association surviving BH correction, the observed associations suggest that the relationship between HFC and the volume fraction of stagnant fluid (F_ISF_) may vary by region rather than show a consistent pattern. Specifically, F_ISF_ exhibited a negative association with HFC in the left hippocampus, but a positive association in the left insula. These regional differences may originate from the distinct pathological microenvironments of these areas; however, these mechanistic interpretations remain speculative and require further validation using modalities such as amyloid PET, tau-PET, CSF biomarker data, or inflammation-sensitive imaging. In the insular region, the positive association may reflect early neuroinflammation or edema-like changes (Park et al., 2022). In this state, an increased interstitial fluid fraction may be accompanied by an influx of inflammatory cells and free ions, which could collectively elevate tissue conductivity. Conversely, the hippocampus is a region that experiences chronic neuronal loss and dense protein accumulation (Braak et al., 2006). The increase in stagnant fluid may fill the space resulting from neuronal death. However, due to the presence of low-conductivity amyloid and tau aggregates, the overall active ionic concentration per volume may decrease, leading to a negative association with HFC. These findings suggest that alterations in conductivity are associated with local microenvironmental changes in selected regions, but do not support a broad, robust, and directionally consistent effect across AD-vulnerable limbic regions.

### 4.2 Correlation between HFC and ISF-related measures in specific brain areas

The partial correlation analysis, adjusting for diagnostic group, age and sex, revealed significant negative correlations between HFC and D_ISF_, most notably in the bilateral corpus callosum and bilateral thalamus, and between HFC and F_ISF_ in the bilateral corpus callosum (Supplementary Table S5). Biologically, this negative relationship may be explained by the physiological representations of the two metrics. When extracellular ISF-related diffusion is impaired, resulting in reduced fluid mobility (lower D_ISF_), the brain’s ability to clear metabolic waste and extracellular ions may be compromised. The subsequent pooling of trapped ions may disrupt local ionic homeostasis, thereby potentially increasing the bulk electrical conductivity (higher HFC) of the tissue (Vitvitsky et al., 2012). These covariate-adjusted correlations suggest limited regional associations between HFC and ISF-related metrics beyond confounding by diagnostic group, age, and sex. Group-stratified analyses and formal interaction testing (Supplementary Table S6) further revealed that the HFC–D_ISF_ association did not show consistent heterogeneity across diagnostic groups after FDR correction (all adjusted interaction *p* > 0.05), indicating that our data do not provide strong evidence for systematic differences in this association across diagnostic groups. This finding should also be interpreted with caution given the limited statistical power of the within-group analyses, particularly in the CN group.

The prominent correlation in these specific regions can be attributed to their unique anatomical characteristics and vulnerability to pathology. The corpus callosum is the major white matter tract in the brain. As AD progresses, extensive demyelination and axonal loss occur in the white matter (Gold et al., 2012). Because the myelin sheath acts as an electrical insulator, its disruption, combined with an increase in extracellular water, alters bulk electrical properties, leading to increased conductivity (Katscher and Den Berg, 2017). Concurrently, alterations in perivascular spaces impede normal ISF transport, contributing to the reduced D_ISF_ (Taoka and Naganawa, 2020).

In addition, the thalamus is a critical relay hub for neural signaling and is known to undergo regional neurodegeneration in AD (Zarei et al., 2010). The accumulation of toxic proteins and the loss of ionic homeostasis in the thalamus profoundly affect membrane-related and ionic microenvironmental properties. These disruptions simultaneously impede fluid-related transport and increase extracellular ionic concentration., driving the strong negative correlation observed between HFC and D_ISF_ (Vitvitsky et al., 2012).

### 4.3 Correlation of ISF-related measures with age and MMSE scores

D_ISF_ was significantly negatively correlated with age (e.g., in the cuneus, middle temporal gyrus, and corpus callosum) and positively correlated with MMSE scores in regions such as the hippocampus and parahippocampal gyrus. Conversely, F_ISF_ was significantly positively correlated with age, but did not show significant associations with MMSE scores after FDR correction. These results are broadly consistent with prior IVIM-based studies. Wong et al. (2020) identified an intermediate diffusion component in 3C-IVIM that reflects aberrant ISF accumulation in lesion-prone regions, demonstrating its relation to enlarged perivascular spaces and white matter hyperintensities in cerebrovascular disease. Extending this approach, Voorter et al. (2023) showed that multi-b-value IVIM-derived ISF-related metrics were sensitive to tissue microenvironmental alterations in cerebrovascular disease, which supports the utility of ISF-related IVIM parameters as markers of extracellular microenvironmental alterations and ISF dysregulation.

It is noteworthy that the voxel-based analyses (Section 3.1.2) did not detect significant associations between any MRI parameter and MMSE scores at the whole-brain level, whereas the ROI-based analyses (Section 3.2.3) revealed significant MMSE correlations for D_ISF_, D_MV_, and F_PAR_ in select regions. These results are not contradictory, but reflect well-recognized differences in sensitivity between the two analytical frameworks. The voxel-based regression applies a stringent brain-wide FDR threshold with a minimum cluster extent of 100 contiguous voxels, requiring spatially distributed effects across a large brain volume. In contrast, ROI-based analyses extract mean values within predefined anatomical structures, reducing intra-regional noise and markedly increasing sensitivity to focal regional effects. The MMSE associations observed in the hippocampus and parahippocampal gyrus reflect region-specific effects in known early AD-vulnerable areas rather than spatially distributed effects detectable at the whole-brain level.

Regarding the association with cognitive status, our findings are supported by pioneering neuroimaging studies of perivascular spaces. Taoka and Naganawa (2020) demonstrated that the DTI-ALPS index, which reflects diffusivity along perivascular spaces and is likely a marker of fractional anisotropy as well as broader perivascular microstructural integrity, rather than a specific measure of glymphatic activity, correlates significantly and positively with MMSE scores in patients with AD.

This relationship has been further substantiated by recent studies, such as Liang et al. (2023), which reported that diffusivity along perivascular spaces is significantly reduced across the MCI–AD spectrum and is associated with cognitive decline. These reports parallel our observation that D_ISF_ decreases with age and correlates positively with cognitive performance. Collectively, our findings extend these prior observations by simultaneously characterizing both D_ISF_ and F_ISF_ components within a unified IVIM framework. As age increases, impairments in aquaporin-4 (AQP4) polarization and perivascular pathways reduce the normal circulation of interstitial fluid. This reduction is reflected in our IVIM model as a decrease in fluid mobility (lower D_ISF_) and an accumulation of stagnant fluid volume (higher FI_SF_). Particularly in the hippocampus, parahippocampal gyrus, and middle temporal gyrus, which are highly vulnerable to early AD pathology, the positive correlation between D_ISF_ and MMSE scores suggests that the disruption of the interstitial space due to Aβ and tau accumulation restricts fluid diffusion. This restriction exacerbates toxic buildup and contributes to cognitive decline (Braak et al., 2006; Harrison et al., 2020).

### 4.4 Limitations of this study

There are several limitations to this study. First, the number of participants in each group was relatively small, and the CN group (*n* = 26) was notably smaller than the MCI (*n* = 78) and AD (*n* = 57) groups. This imbalance reduces statistical power for contrasts involving CN, and findings based on CN-specific comparisons should be interpreted with caution. Although HC3 heteroscedasticity-robust ANCOVA was applied to address potential variance heterogeneity, a larger and more balanced CN cohort remains necessary for full validation. Second, we used a single-echo 3D turbo spin-echo (TSE) sequence for MREPT data acquisition.

Alternative acquisition approaches, such as 3D balanced sequences or two-dimensional multi-echo TSE sequences, are increasingly used for conductivity mapping. Future studies utilizing these sequences are encouraged to validate and extend our findings. Third, we measured bulk HFC using phase-based MREPT without separating it into intra-neurite and extra-neurite components, as we did not acquire the necessary diffusion tensor imaging data for this compartmental model. Therefore, future research combining MREPT with multi-shell diffusion MRI is essential to clarify compartment-specific electrical alterations, particularly extra-neurite conductivity. Fourth, we used IVIM metrics to calculate ISF-related parameters; however, alternative quantification approaches, such as contrast agent-based methods, could be explored for independent validation of ISF dynamics. Fifth, automated eddy current correction and rigid-body motion correction were not applied as separate preprocessing steps for the DWI-EPI data, which may have introduced residual artifacts. Additionally, no field map-based distortion correction or reverse phase-encoding distortion correction was applied to the DWI-EPI data.

Therefore, geometric distortion related to B0 field inhomogeneity may have affected spatial correspondence between IVIM-derived maps and anatomical ROIs, particularly in susceptibility-prone regions. Sixth, although the phase-based MREPT reconstruction employs a magnitude-constrained locally adaptive kernel that inherently limits CSF contribution at tissue boundaries, potential residual CSF partial volume effects at gray matter–CSF interfaces cannot be entirely excluded, particularly in severely atrophied AD brains. A CSF partial volume sensitivity analysis was performed as a supplementary analysis (detailed results are provided in the revised Supplementary material), and the findings should be interpreted in the context of this potential confound. In addition, the 10 mm FWHM Gaussian smoothing kernel used for voxel-based analyses may have reduced spatial specificity, particularly for the higher-resolution HFC maps, in small structures, and in atrophied AD brains. Seventh, the ROC curve analyses reported in Supplementary Table S7 were performed on the same dataset used for group comparisons and should therefore be interpreted as exploratory. The reported AUC values may be optimistic and should be regarded as within-sample performance estimates rather than definitive evidence of generalizable diagnostic utility. Finally, in this study, the conductivity differences among various forms of amyloid (e.g., monomers, oligomers, and dense plaques) were not investigated. Because smaller ions generally exhibit higher conductivity due to lower hydrodynamic resistance in solution, the bulk conductivity values may differ depending on the specific amyloid state and local inflammatory response. Investigating these molecular-level conductivity differences using animal models could provide deeper insights.

## 5 Conclusion

This study characterized the *in vivo* relationship between MREPT-derived HFC and IVIM-derived ISF-related metrics across the spectrum of cognitive impairment. Our results showed that as patients transition from cognitively normal to Alzheimer’s disease, there is a significant reduction in fluid mobility (D_ISF_) and a concurrent increase in both the fluid volume fraction (F_ISF_) and MREPT-derived HFC. Furthermore, we observed limited and region-specific independent associations between selected ISF-related indices and HFC, with the most robust finding being the association of D_ISF_ in the left thalamus. In the volume-fraction model, F_PAR_ exhibited the most consistent independent association with HFC, while associations with F_ISF_-related metrics were not robust after correction. These findings suggest localized associations between MREPT-derived HFC and IVIM-derived ISF-related metrics, rather than a broad HFC–ISF coupling. Combining MREPT-derived HFC and IVIM-derived ISF-related metrics may provide a non-invasive imaging framework to investigate ionic and fluid-related microenvironmental changes in cognitive impairment; however, replication in larger independent cohorts is required.

## Data Availability

The original contributions presented in the study are included in the article/Supplementary material, further inquiries can be directed to the corresponding authors.

## Footnotes

1
http://www.fil.ion.ucl.ac.uk/spm/software/spm12

2
http://www.neuro.uni-jena.de/cat/

3
http://fmri.wfubmc.edu/software/PickAtlas

4
http://marsbar.sourceforge.net
