# Supplementary material for: Evaluation of the association between MREPT-derived conductivity and IVIM-derived ISF-related metrics in the brains of patients with cognitive impairments
Source: Front Aging Neurosci. 2026 Jun 10;18:1828803. doi: 10.3389/fnagi.2026.1828803 (PMC13290988; doi:10.3389/fnagi.2026.1828803)
Supplement: Supplementary file 1 [file Supplementary_file_1.docx]

Evaluation of the Association between MREPT-Derived Conductivity and IVIM-Derived ISF-Related Metrics in the Brains of Patients with Cognitive Impairments

**Supplementary**

**
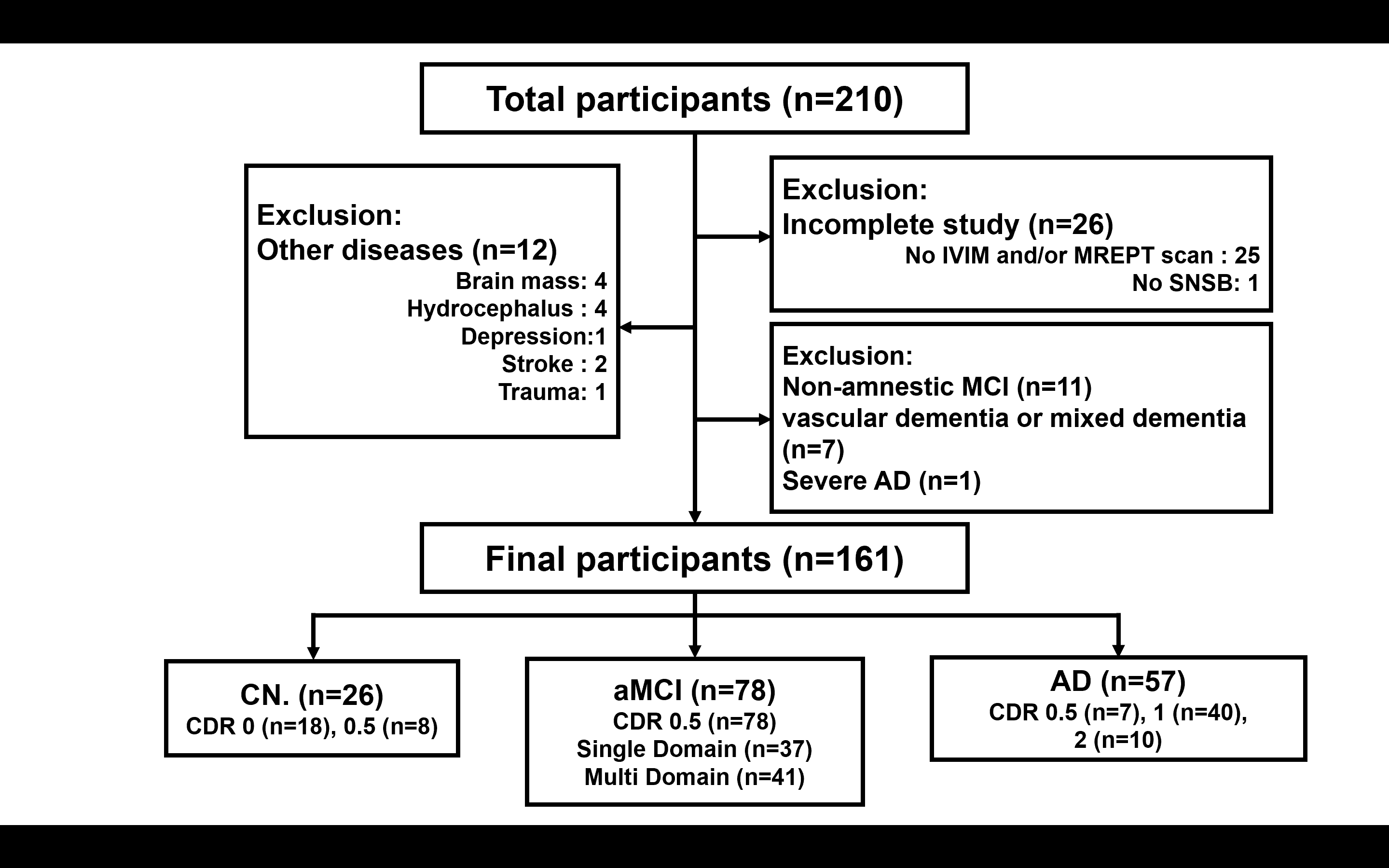
**

**Supplementary Figure S1. Flowchart summarizing the selection process of the study participants.**

*Abbreviation*: intravoxel incoherent motion (IVIM), magnetic resonance electrical property tomography (MREPT), Seoul Neuropsychological Screening Battery (SNSB), cognitively normal (CN), amnestic mild cognitive impairment (aMCI), Alzheimer’s disease (AD), clinical dementia rating (CDR)


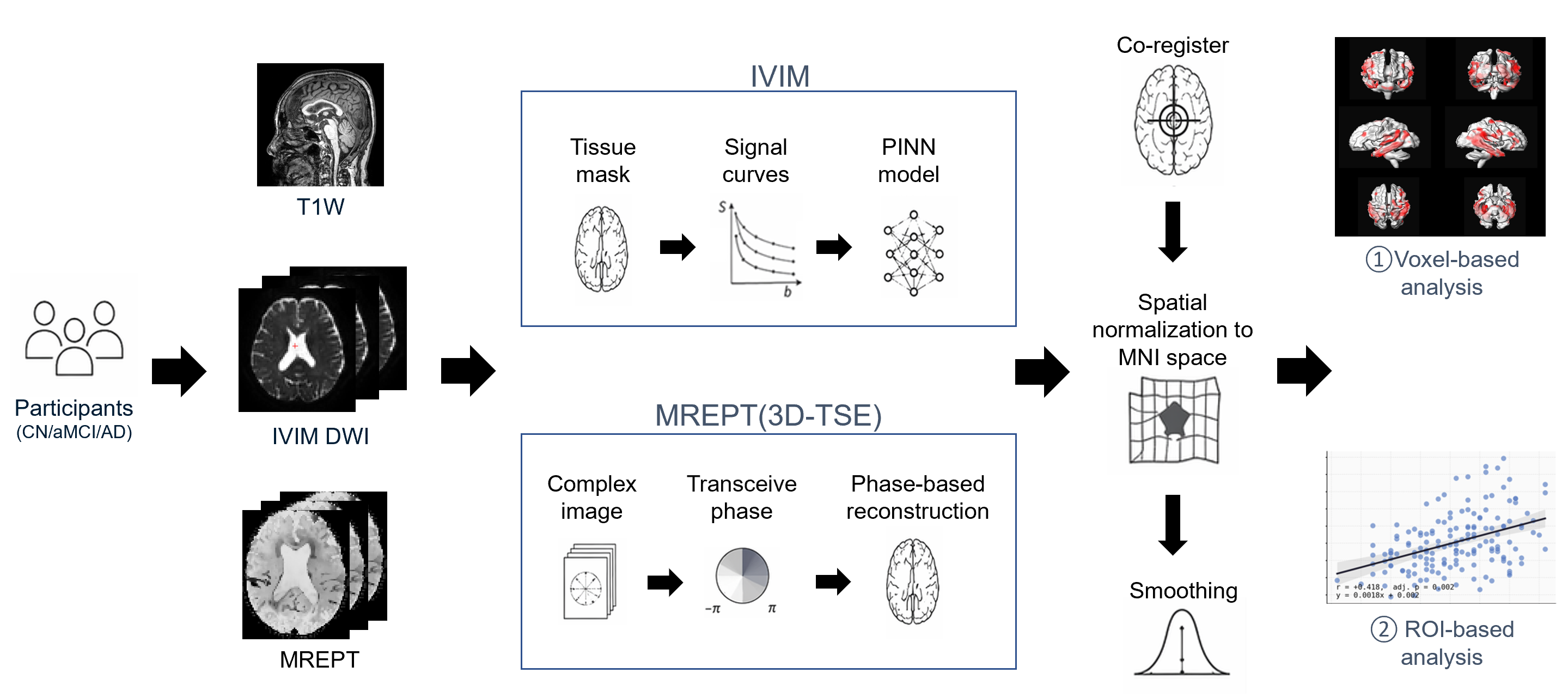


**Supplementary Figure S2. Complete preprocessing and analysis workflow for IVIM-ISF and MREPT-HFC mapping.**

Schematic overview of the study workflow from MRI acquisition to statistical analysis. IVIM diffusion-weighted images were used for tissue-masked PINN-based three-component IVIM fitting to generate D_ISF_, F_ISF_, D_PAR_, D_MV_, F_PAR_, and F_MV_ maps. MREPT data were reconstructed from complex image data using transceive-phase-based conductivity reconstruction to generate HFC maps. All reconstructed maps were co-registered to the 3D T1W image, spatially normalized to the AD-specific template, and Gaussian smoothing was applied for voxel-based analyses. Final analyses included voxel-based and ROI-based statistical analyses.

*Abbreviation: intravoxel incoherent motion (IVIM), interstitial fluid (ISF), magnetic resonance electrical property tomography (MREPT), high-frequency conductivity (HFC), physics-informed neural network (PINN), three-dimensional T1-weighted image (3D T1W), region of interest (ROI).*

**Supplementary Figure S3. Result maps of the voxel-based group comparison among the three participant groups of MRI measures**

**
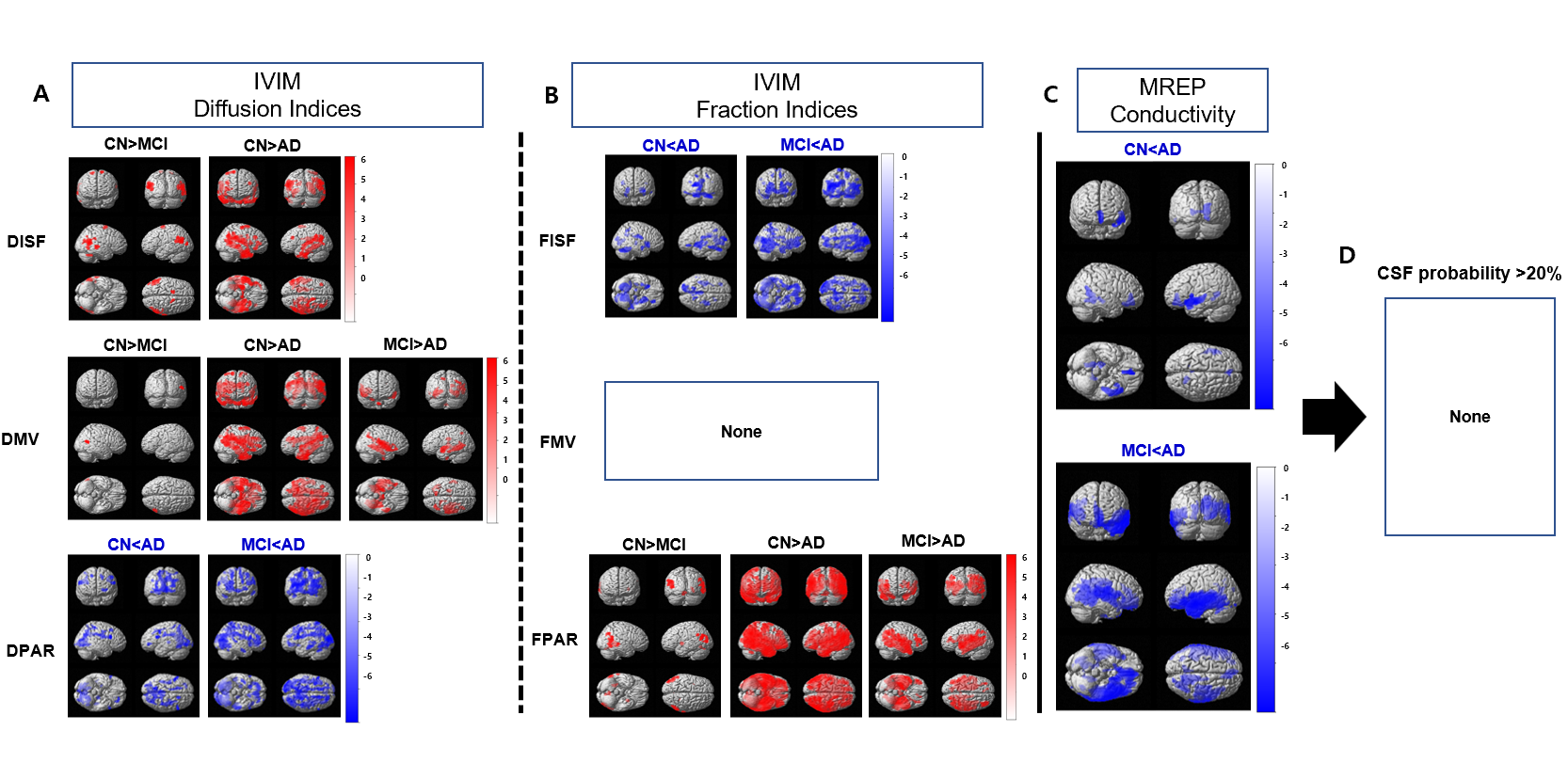
**

***Abbreviation:*** *intravoxel incoherent motion (IVIM), parenchymal diffusion (Dpar), an intermediate diffusion component interpreted as ISF-related diffusion (Disf), a microvascular pseudo-diffusion component (Dmv), parenchymal volume fractions (Fpar), ISF volume fractions (Fisf), microvascular volume fractions (Fmv).*

**(A–C)** Original voxel-based analysis results demonstrating significant group differences in HFC without explicit CSF masking.
**(D)** Results of the supplementary sensitivity analysis after explicitly masking cerebrospinal fluid (CSF) utilizing individual 3D T1-weighted tissue segmentations. Following rigorous CSF masking, the voxel-wise group differences in HFC did not survive multiple comparisons correction, indicating that the bulk conductivity alterations observed in the unmasked analysis are closely coupled with the expansion of extracellular fluid and CSF compartments.

**Supplementary Table S1. The information on the cluster size and location, Talairach coordinates, and the corresponding Z-score for the results of the voxel-based group comparison among the three participant groups of MRI measures**

**1A. D_ISF_**

| **Group analysis** | **Cluster size** | **Cluster location** | **BA** | **Talairach coordinates** | **Z score** | **ROI** |
| --- | --- | --- | --- | --- | --- | --- |
| **CN < aMCI** | None |  |  |  |  |  |
| **CN > aMCI** | 758 | Lt Middle Temporal Gyrus WM |  | -45.81, -62.73, 13.78 | 4.81 |  |
|  |  | Lt Supramarginal Gyrus WM |  | -53.35, -52.73, 27.21 | 4.57 |  |
|  |  | Lt Superior Temporal Gyrus WM |  | -56.87, -47.76, 15.01 | 3.81 |  |
|  | 601 | Rt Middle Temporal Gyrus WM |  | 48.65, -55.41, 12.46 | 4.69 |  |
|  |  | Rt Middle Occipital Gyrus WM |  | 39.31, -70.62, 14.47 | 3.85 |  |
|  | 203 | Rt Middle Temporal Gyrus GM | 21 | 65.54, -28.2, 2.71 | 4.31 |  |
| **CN < AD** | None |  |  |  |  |  |
| **CN > AD** | 4988 | Rt Transverse Temporal Gyrus GM | 41 | 46.91, -21.69, 13.83 | 5.20 |  |
|  |  | Rt Middle Temporal Gyrus WM |  | 46.85, -55.06, 8.86 | 4.42 |  |
|  |  | Rt Middle Temporal Gyrus GM | 21 | 51.15, 5.99, -19.51 | 4.37 |  |
|  | 9408 | Rt Parahippocampal Gyrus WM |  | 22.99, -38.81, -2.61 | 5.02 |  |
|  |  | Lt Transverse Temporal Gyrus WM |  | -47.46, -22.89, 10.31 | 4.76 |  |
|  |  | Rt Parahippocampal Gyrus WM |  | 23.39, -10.28, -25.13 | 4.72 |  |
|  | 581 | Rt Precentral Gyrus GM | 4 | 33.33, -22.6, 62.16 | 4.10 |  |
|  |  | Rt Superior Frontal Gyrus GM | 6 | 12.96, -6.08, 66.98 | 3.84 |  |
|  |  | Rt Precentral Gyrus GM | 6 | 20.36, -18.98, 64.08 | 3.35 |  |
|  | 208 | Rt Middle Frontal Gyrus GM | 9 | 33.89, 23.56, 32.31 | 3.79 |  |
|  | 156 | Lt Middle Frontal Gyrus GM | 9 | -38.3, 23.93, 31.12 | 3.78 |  |
|  | 202 | Rt Middle Temporal Gyrus WM |  | 61.89, -35.11, -3.41 | 3.67 |  |
|  |  | Rt Superior Temporal Gyrus GM |  | 63.69, -26.32, 2.86 | 3.40 |  |
|  | 102 | Rt Middle Temporal Gyrus WM |  | 54.49, -53.35, -8.86 | 3.36 |  |
|  | 215 | Rt Inferior Frontal Gyrus WM |  | 39.91, 25.15, -3.48 | 3.31 |  |
|  |  | Rt Inferior Frontal Gyrus WM |  | 39.81, 31.73, 6.16 | 3.14 |  |
|  | 224 | Rt Precentral Gyrus GM | 6 | 46.67, -7.02, 36.83 | 3.25 |  |
|  |  | Rt Sub-Gyral WM |  | 30.1, -0.82, 31.73 | 3.21 |  |
|  | 314 | Lt Thalamus GM |  | -8.51, -18.85, 5.95 | 3.21 |  |
|  |  | Lt Caudate GM |  | -17.99, -24.09, 21.51 | 3.16 |  |
|  | 170 | Lt Sub-Gyral WM |  | -14.39, -46.82, 23.02 | 3.13 |  |
|  | 107 | Rt Sub-Gyral WM |  | 37.77, 32.21, 20.58 | 3.13 |  |
|  |  |  |  |  |  |  |

**1B. D_MV_**

| **Group analysis** | **Cluster size** | **Cluster location** | **BA** | **Talairach coordinates** | **Z score** | **ROI** |
| --- | --- | --- | --- | --- | --- | --- |
| **CN < aMCI** | None |  |  |  |  |  |
| **CN > aMCI** | None |  |  |  |  |  |
| **CN < AD** | None |  |  |  |  |  |
| **CN > AD** | 36536 | Rt Parahippocampal Gyrus WM |  | 17.32, -46.93, 3.73 | 5.21 |  |
|  |  | Rt Transverse Temporal Gyrus GM | 41 | 46.91, -21.69, 13.83 | 4.99 |  |
|  |  | Rt Parahippocampal Gyrus GM |  | 26.97, -13.04, -16.32 | 4.98 |  |
|  | 604 | Rt Superior Frontal Gyrus WM |  | 12.99, -4.04, 65.37 | 4.22 |  |
|  |  | Rt Precentral Gyrus WM |  | 33.32, -24.47, 61.98 | 3.56 |  |
|  |  | Rt Precentral Gyrus WM |  | 24.06, -19, 64.14 | 3.27 |  |
|  | 345 | Lt Middle Frontal Gyrus WM |  | -14.76, -11.17, 62.43 | 3.39 |  |
|  | 234 | Rt Medial Frontal Gyrus WM |  | 21.26, 44.35, 12.44 | 2.98 |  |
|  |  | Rt Sub-Gyral WM |  | 30.48, 40.4, 14.03 | 2.89 |  |
| **aMCI < AD** | None |  |  |  |  |  |
| **aMCI > AD** | 5175 | Rt Parahippocampal Gyrus GM | 27 | 21.18, -31.17, -3.72 | 5.57 |  |
|  |  | Rt Parahippocampal Gyrus WM |  | 13.62, -46.91, 3.67 | 4.74 |  |
|  |  | Rt Transverse Temporal Gyrus GM | 41 | 45.06, -21.68, 13.8 | 4.72 |  |
|  | 417 | Lt Superior Temporal Gyrus WM |  | -47.1, 1.73, -10.77 | 4.19 |  |
|  | 214 | Rt Precuneus WM |  | 29.79, -65.21, 36.44 | 3.79 |  |
|  |  | Rt Angular Gyrus WM |  | 42.83, -59.17, 31.83 | 3.46 |  |
|  |  |  |  |  |  |  |

**1C. D_PAR_**

| **Group analysis** | **Cluster size** | **Cluster location** | **BA** | **Talairach coordinates** | **Z score** | **ROI** |
| --- | --- | --- | --- | --- | --- | --- |
| **CN < aMCI** | None |  |  |  |  |  |
| **CN > aMCI** | None |  |  |  |  |  |
| **CN < AD** | 4767 | Lt Posterior Cingulate WM |  | -5.04, -60.73, 11.05 | 5.41 |  |
|  |  | Rt Precuneus GM | 7 | 7.64, -61.02, 32.86 | 5.30 |  |
|  |  | Lt Cingulate Gyrus WM |  | -5.24, -40.28, 32.80 | 4.91 |  |
|  | 798 | Rt Sub-Gyral WM |  | 33.75, -21.33, 29.85 | 5.08 |  |
|  |  | Rt Extra-Nuclear WM |  | 20.92, -14.80, 21.25 | 3.58 |  |
|  | 410 | Lt Sub-Gyral WM |  | -34.75, -17.42, 30.87 | 4.44 |  |
|  |  | Lt Inferior Parietal Lobule WM |  | -38.50, -37.90, 28.86 | 4.33 |  |
|  | 216 | Rt Inferior Frontal Gyrus WM |  | 46.88, 10.97, 25.93 | 4.07 |  |
|  | 158 | Lt Cingulate Gyrus GM | 24 | -18.19, 7.36, 46.11 | 3.96 |  |
|  |  | Lt Medial Frontal Gyrus WM |  | -14.56, -4.19, 48.68 | 3.43 |  |
|  | 119 | Lt Inferior Frontal Gyrus WM |  | -54.92, 11.50, 24.26 | 3.75 |  |
|  | 136 | Rt Cuneus GM | 18 | 4.00, -89.77, 19.26 | 3.45 |  |
|  |  | Lt Cuneus GM | 18 | -7.08, -89.54, 17.30 | 3.22 |  |
|  | 118 | Lt Anterior Cingulate WM |  | -21.24, 43.23, 6.21 | 3.40 |  |
|  |  | Lt Sub-Gyral WM |  | -30.51, 37.69, 5.53 | 3.22 |  |
| **CN > AD** | None |  |  |  |  |  |
| **aMCI < AD** | 19608 | Rt Precuneus WM |  | 14.90, -45.68, 48.85 | 5.71 |  |
|  |  | Rt Medial Frontal Gyrus WM |  | 15.51, 39.26, 26.28 | 5.41 |  |
|  |  | Lt Inferior Parietal Lobule WM |  | -38.49, -34.17, 29.22 | 5.23 |  |
|  | 463 | Lt Middle Temporal Gyrus WM |  | -54.62, -26.53, -9.97 | 3.69 |  |
|  |  | Lt Sub-Gyral WM |  | -43.72, -35.43, 3.78 | 3.52 |  |
|  | 185 | Lt Middle Frontal Gyrus WM |  | -36.73, 1.69, 47.06 | 3.56 |  |
|  | 214 | Rt Inferior Frontal Gyrus WM |  | 58.01, 12.95, 24.50 | 3.55 |  |
|  | 191 | Rt Sub-Gyral WM |  | 41.78, -9.39, -15.72 | 3.36 |  |
|  | 106 | Lt Culmen GM |  | -35.96, -49.28, -28.03 | 3.32 |  |
|  | 215 | Lt Superior Frontal Gyrus GM | 11 | -13.51, 63.91, -13.32 | 3.31 |  |
|  |  |  |  |  |  |  |
| **aMCI > AD** | None |  |  |  |  |  |

**1D. F_ISF_**

| **Group analysis** | **Cluster size** | **Cluster location** | **BA** | **Talairach coordinates** | **Z score** | **ROI** |
| --- | --- | --- | --- | --- | --- | --- |
| **CN < aMCI** | None |  |  |  |  |  |
| **CN > aMCI** | None |  |  |  |  |  |
| **CN < AD** | 529 | Lt Cingulate Gyrus GM | 31 | -3.34, -32.67, 31.75 | 4.98 |  |
|  |  | Lt Posterior Cingulate GM | 23 | -3.29, -46.88, 23.20 | 3.74 |  |
|  |  | Rt Cingulate Gyrus GM | 31 | 4.02, -56.75, 27.80 | 3.57 |  |
|  | 132 | Rt Extra-Nuclear WM |  | 28.78, -2.39, -9.88 | 4.79 |  |
|  | 492 | Rt Extra-Nuclear WM |  | 35.69, -11.50, 25.41 | 4.61 |  |
|  |  | Rt Sub-Gyral WM |  | 28.24, -22.82, 26.02 | 4.52 |  |
|  | 1781 | Lt Lentiform Nucleus GM |  | -26.80, 2.97, -4.90 | 4.60 |  |
|  |  | Lt Extra-Nuclear WM |  | -30.50, -4.29, -7.46 | 4.54 |  |
|  |  | Lt Sub-Gyral WM |  | -38.43, -37.38, 23.51 | 4.03 |  |
|  | 321 | Rt Lentiform Nucleus GM |  | 21.30, 15.41, 0.69 | 4.43 |  |
|  | 1552 | Lt Cuneus WM |  | -21.76, -86.72, 8.30 | 4.34 |  |
|  |  | Lt Posterior Cingulate WM |  | -5.03, -58.86, 11.23 | 4.30 |  |
|  |  | Lt Lingual Gyrus GM |  | -8.63, -70.84, -0.78 | 3.97 |  |
|  | 924 | Rt Culmen GM |  | 26.93, -53.50, -25.56 | 4.01 |  |
|  | 102 | Lt Precuneus WM |  | -18.38, -58.03, 41.71 | 3.98 |  |
|  | 992 | Lt Declive GM |  | -19.44, -64.97, -22.03 | 3.96 |  |
|  |  | Lt Sub-Gyral WM |  | -50.94, -34.00, -10.62 | 3.75 |  |
|  |  | Lt Culmen GM |  | -8.38, -56.23, -15.61 | 3.71 |  |
| **CN > AD** | None |  |  |  |  |  |
| **aMCI < AD** | 9711 | Lt Middle Occipital Gyrus GM | 19 | -40.23, -80.86, 6.75 | 5.15 |  |
|  |  | Lt Extra-Nuclear WM |  | -38.39, -22.47, 24.92 | 4.66 |  |
|  |  | Lt Sub-Gyral WM |  | -38.46, -39.41, 25.12 | 4.57 |  |
|  | 1336 | Rt Precuneus GM | 7 | 13.04, -49.40, 48.46 | 4.80 |  |
|  |  | Rt Precuneus WM |  | 9.29, -68.01, 46.64 | 3.04 |  |
|  | 5990 | Rt Middle Occipital Gyrus WM |  | 30.13, -70.05, 8.96 | 4.77 |  |
|  |  | Rt Culmen GM |  | 30.61, -53.70, -23.71 | 4.12 |  |
|  |  | Rt Postcentral Gyrus WM |  | 37.51, -15.41, 26.87 | 4.09 |  |
|  | 1105 | Lt Posterior Cingulate GM | 23 | -3.29, -46.88, 23.20 | 4.71 |  |
|  | 5713 | Rt Parahippocampal Gyrus GM |  | 26.95, -2.21, -11.69 | 4.58 |  |
|  |  | Rt Medial Frontal Gyrus GM | 10 | 19.40, 40.63, 12.06 | 4.57 |  |
|  |  | Rt Extra-Nuclear WM |  | 21.19, 16.40, 9.79 | 4.28 |  |
|  | 807 | Lt Uvula GM |  | -13.85, -68.37, -25.86 | 3.41 |  |
|  |  | Lt Declive GM |  | -2.90, -67.79, -13.00 | 3.29 |  |
|  | 181 | Lt Thalamus GM |  | -12.22, -27.97, 3.22 | 3.22 |  |
|  | 111 | Lt Sub-Gyral WM |  | -16.49, -26.71, 48.31 | 3.21 |  |
|  | 112 | Rt Inferior Frontal Gyrus WM |  | 59.86, 12.94, 24.53 | 3.14 |  |
|  |  |  |  |  |  |  |
| **aMCI > AD** | None |  |  |  |  |  |

**1E. F_MV_ None**

**1F. F_PAR_**

| **Group analysis** | **Cluster size** | **Cluster location** | **BA** | **Talairach coordinates** | **Z score** | **ROI** |
| --- | --- | --- | --- | --- | --- | --- |
| **CN < aMCI** | None |  |  |  |  |  |
| **CN > aMCI** | 206 | Rt Parahippocampal Gyrus WM |  | 23.03, -42.19, -6.54 | 4.61 |  |
|  | 324 | Lt Middle Temporal Gyrus WM |  | -51.42, -66.77, 16.90 | 4.44 |  |
|  | 283 | Rt Superior Temporal Gyrus WM |  | 56.01, -53.94, 16.33 | 4.19 |  |
|  |  | Rt Middle Temporal Gyrus WM |  | 56.13, -54.93, 7.23 | 4.16 |  |
|  |  | Rt Middle Temporal Gyrus GM | 37 | 56.33, -55.23, -9.01 | 3.75 |  |
|  | 199 | Lt Middle Occipital Gyrus WM |  | -42.03, -78.64, 3.32 | 4.13 |  |
| **CN < AD** | None |  |  |  |  |  |
| **CN > AD** | 96850 | Rt Parahippocampal Gyrus WM |  | 23.03, -42.19, -6.54 | 7.40 |  |
|  |  | Lt Parahippocampal Gyrus WM |  | -25.10, -34.66, -4.84 | 6.19 |  |
|  |  | Rt Extra-Nuclear WM |  | 28.77, -4.25, -10.05 | 5.88 |  |
|  | 385 | Lt Declive GM |  | -2.88, -58.47, -12.12 | 3.33 |  |
|  | 121 | Rt Tuber GM |  | 52.90, -56.84, -30.84 | 2.93 |  |
|  | 121 | Lt Declive of Vermis GM |  | -2.79, -70.64, -22.28 | 2.66 |  |
| **CN > AD** | None |  |  |  |  |  |
| **aMCI < AD** | None |  |  |  |  |  |
| **aMCI > AD** | 28396 | Rt Extra-Nuclear WM |  | 30.25, -30.75, 10.88 | 5.67 |  |
|  |  | Rt Parahippocampal Gyrus WM |  | 19.30, -35.06, -2.32 | 5.53 |  |
|  |  | Lt Parahippocampal Gyrus WM |  | -23.25, -34.67, -4.81 | 5.50 |  |
|  | 1073 | Rt Middle Frontal Gyrus WM |  | 34.10, 36.13, 19.09 | 4.18 |  |
|  |  | Rt Middle Frontal Gyrus GM | 9 | 33.89, 23.56, 32.31 | 3.60 |  |
|  |  | Rt Inferior Frontal Gyrus WM |  | 39.85, 32.08, 2.59 | 3.17 |  |
|  | 169 | Rt Precentral Gyrus WM |  | 31.50, -22.42, 60.34 | 3.38 |  |
|  |  | Rt Middle Frontal Gyrus WM |  | 33.59, -9.68, 45.37 | 2.75 |  |
|  | 116 | Lt Postcentral Gyrus GM | 2 | -49.76, -24.33, 44.37 | 3.22 |  |
|  |  | Lt Postcentral Gyrus WM |  | -45.96, -25.52, 37.12 | 3.03 |  |

**1G. HFC**

| **Group analysis** | **Cluster size** | **Cluster location** | **BA** | **Talairach coordinates** | **Z score** | **ROI** |
| --- | --- | --- | --- | --- | --- | --- |
| **CN < aMCI** | None |  |  |  |  |  |
| **CN > aMCI** | None |  |  |  |  |  |
| **CN < AD** | 1394 | Lt Superior Temporal Gyrus GM | 22 | -45.36, -8.29, -4.48 | 4.44 |  |
|  |  | Lt Superior Temporal Gyrus GM | 22 | -47.16, 4.93, -5.06 | 4.26 |  |
|  |  | Lt Insula GM | 13 | -39.96, -22.23, 3.30 | 3.74 |  |
|  | 1357 | Rt Posterior Cingulate GM | 30 | 17.23, -54.90, 8.38 | 4.42 |  |
|  |  |  |  | 4.36, -43.31, 5.65 | 4.08 |  |
|  |  | Rt Culmen GM |  | 17.52, -41.81, -10.20 | 3.99 |  |
|  | 516 | Lt Anterior Cingulate GM | 24 | -2.70, 37.89, 2.42 | 4.05 |  |
|  |  | Lt |  | -0.75, 24.02, -9.67 | 3.39 |  |
| **CN > AD** | None |  |  |  |  |  |
| **aMCI < AD** | 21967 | Lt Middle Temporal Gyrus WM |  | -54.00, 0.00, -14.00 | 6.07 |  |
|  |  | Lt Sub-Gyral WM |  | -40.00, -10.00, -16.00 | 5.05 |  |
|  |  | Lt Middle Temporal Gyrus GM | 21 | -64.00, -16.00, -14.00 | 4.87 |  |
|  | 12111 | Rt Posterior Cingulate WM |  | 16.00, -56.00, 16.00 | 4.97 |  |
|  |  | Rt Precentral Gyrus WM |  | 46.00, 12.00, 8.00 | 4.07 |  |
|  |  | Rt Precentral Gyrus GM | 43 | 50.00, -8.00, 12.00 | 4.05 |  |
|  | 196 | Lt Sub-Gyral WM |  | -34.00, -68.00, 0.00 | 3.36 |  |
|  | 777 | Lt Posterior Cingulate WM |  | -10.00, -58.00, 8.00 | 3.35 |  |
|  |  | Lt |  | -16.00, -44.00, -4.00 | 3.07 |  |
|  |  | Lt Precuneus WM |  | -8.00, -60.00, 18.00 | 3.06 |  |
| **aMCI > AD** | None |  |  |  |  |  |

Three-group ANCOVA was performed for voxel-based analyses to compare the CN, aMCI, and AD groups with age and sex as covariates. Post hoc pairwise contrasts were tested using a voxel-wise FDR-corrected threshold of p = 0.05 and clusters with at least 100 contiguous voxels.

**Supplementary Figure S4. Result maps of the voxel-based multiple regression with the participant’s age**

**
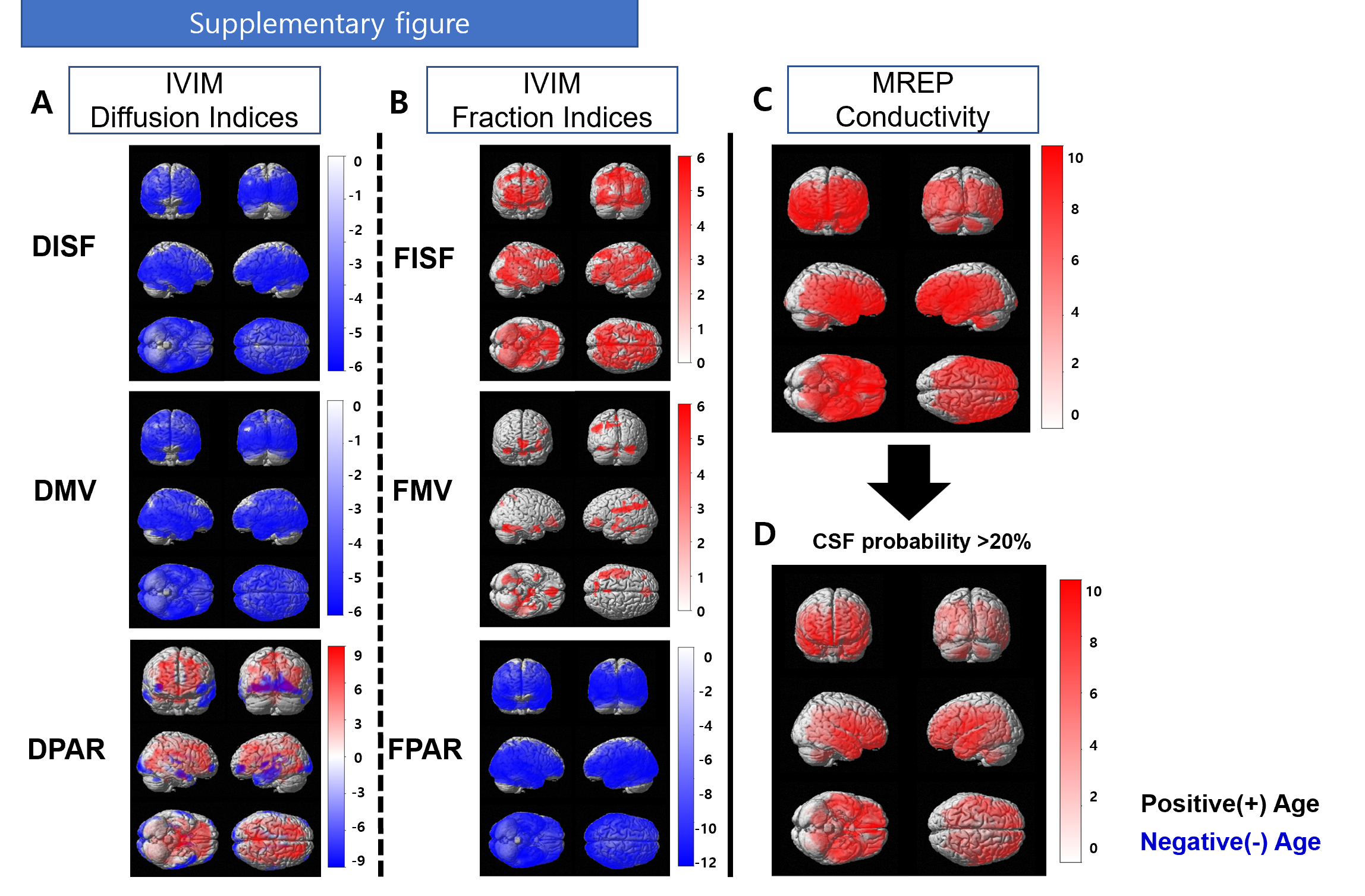
**

***Abbreviation:*** *intravoxel incoherent motion (IVIM), parenchymal diffusion (Dpar), an intermediate diffusion component interpreted as ISF-related diffusion (Disf), a microvascular pseudo-diffusion component (Dmv), parenchymal volume fractions (Fpar), ISF volume fractions (Fisf), microvascular volume fractions (Fmv).*

(A–C) Original voxel-based analysis results without CSF masking, showing significant associations. (D) The result of the supplementary sensitivity analysis for the association between HFC and age after explicitly masking cerebrospinal fluid (CSF) using individual 3D T1-weighted images. While the voxel-wise group differences did not survive multiple comparisons after CSF masking, the positive correlation between HFC and age remained significant in specific regions, as shown in (D).

**Supplementary Table S2. The information on the cluster size and location, Talairach coordinates, and the corresponding Z-score for the results of the voxel-based multiple regression with the participant’s age**

| **Index** | **Group analysis** | **Cluster size** | **Cluster location** | **BA** | **Talairach coordinates** | **Z score** | **ROI** |
| --- | --- | --- | --- | --- | --- | --- | --- |
| $\mathbf{D}_{\mathbf{ISF}}$ | **+Age** | None |  |  |  |  |  |
|  | **-Age** | 147867 | Lt Middle Temporal Gyrus GM | 21 | -60.14, -13.46, -8.83 | 7.19 |  |
|  |  |  | Lt Lingual Gyrus GM | 18 | -3.14, -78.67, 2.18 | 6.99 |  |
|  |  |  | Lt Extra-Nuclear WM |  | -12.49, -35.47, 22.33 | 6.86 |  |
|  |  | 23 | Rt Superior Parietal Lobule WM |  | 22.12, -52.53, 60.93 | 2.13 |  |
|  |  | 6 | Lt Pyramis GM |  | -43.38, -73.11, -34.01 | 2.00 |  |
|  |  | 3 | Lt Precentral Gyrus WM |  | -14.86, -21.01, 66.9 | 1.95 |  |
| $\mathbf{D}_{\mathbf{MV}}$ | **+Age** | None |  |  |  |  |  |
|  | **-Age** | 172507 | Rt Thalamus GM |  | 4.47, -9.6, 7.05 | 65535 |  |
|  |  |  | Lt Middle Temporal Gyrus WM |  | -60.13, -11.59, -8.65 | 7.74 |  |
|  |  |  | Rt Extra-Nuclear WM |  | 19.26, -33.55, 1.42 | 7.70 |  |
|  |  | 46 | Lt |  | -8.09, -22.99, -28.67 | 2.14 |  |
| $\mathbf{D}_{\mathbf{PAR}}$ | **+Age** | None |  |  |  |  |  |
|  | **-Age** | 59337 | Lt Posterior Cingulate GM | 30 | -3.23, -50.08, 17.50 | 65535 |  |
|  |  |  | Lt Thalamus GM |  | -14.08, -24.41, 5.33 | 7.83 |  |
|  |  |  | Rt Sub-Gyral WM |  | 30.05, -19.45, 29.97 | 7.62 |  |
|  |  | 105 |  |  | 2.20, 45.85, 55.50 | 2.64 |  |
| $\mathbf{F}_{\mathbf{ISF}}$ | **+Age** | 59824 | Lt |  | 0.32, -38.45, 33.07 | 6.66 |  |
|  |  |  | Rt |  | 4.08, -52.68, 24.58 | 6.21 |  |
|  |  |  | Lt Thalamus GM |  | -14.06, -26.10, 3.37 | 6.13 |  |
|  | **-Age** | None |  |  |  |  |  |
| $\mathbf{F}_{\mathbf{MV}}$ | **+Age** | 1960 | Lt Inferior Parietal Lobule WM |  | -45.81, -28.02, 24.27 | 4.88 |  |
|  |  |  | Lt Middle Temporal Gyrus WM |  | -40.47, -64.32, 29.93 | 4.14 |  |
|  |  |  | Lt Inferior Parietal Lobule WM |  | -51.46, -37.83, 28.65 | 3.91 |  |
|  |  | 1021 | Lt Anterior Cingulate GM |  | -6.41, 36.05, 2.18 | 4.72 |  |
|  |  |  | Rt Anterior Cingulate GM | 24 | 4.68, 37.68, 4.32 | 4.49 |  |
|  |  |  | Rt Anterior Cingulate GM |  | 1.06, 43.81, -0.56 | 3.98 |  |
|  |  | 335 | Rt Sub-Gyral WM |  | 30.71, 3.71, -14.67 | 4.46 |  |
|  |  | 1261 | Rt Declive GM |  | 32.3, -64.07, -13.86 | 4.37 |  |
|  |  |  | Rt Declive GM |  | 24.99, -63.33, -21.12 | 4.28 |  |
|  |  |  | Rt Parahippocampal Gyrus WM |  | 25.02, -33.87, -14.72 | 4.20 |  |
|  |  | 1548 |  |  | 1.12, -6.96, -19.79 | 4.14 |  |
|  |  |  | Lt |  | -28.63, -33.42, -17.39 | 3.97 |  |
|  |  |  | Lt Declive GM |  | -38.01, -61.67, -16.62 | 3.83 |  |
|  |  | 408 | Lt Sub-Gyral WM |  | -37.89, -5.94, -9.54 | 4.11 |  |
|  |  |  | Lt Extra-Nuclear WM |  | -36.21, -18.18, 0.14 | 3.86 |  |
|  |  |  | Lt Insula WM |  | -36.26, -11.25, 6.2 | 3.51 |  |
|  |  | 146 | Lt Sub-Gyral WM |  | -32.28, 32.81, -2.17 | 3.71 |  |
|  |  |  | Lt Inferior Frontal Gyrus WM |  | -36.09, 33.82, 6.87 | 3.49 |  |
|  |  | 298 | Lt Precuneus WM |  | -20.13, -73.74, 29.38 | 3.59 |  |
|  |  |  | Lt |  | -12.78, -75.99, 32.9 | 3.40 |  |
|  |  |  | Rt Cuneus GM | 19 | 3.84, -79.98, 34.6 | 3.19 |  |
|  |  | 104 | Lt Precuneus GM | 7 | -3.64, -49.49, 49.97 | 3.49 |  |
|  |  |  | Lt Precuneus GM | 7 | -3.76, -57.64, 56.41 | 3.16 |  |
|  | **-Age** | None |  |  |  |  |  |
| $\mathbf{F}_{\mathbf{PAR}}$ | **+Age** | None |  |  |  |  |  |
|  | **-Age** | 191151 | Rt Extra-Nuclear WM |  | 34.22, -15.98, -5.67 | 65535 |  |
|  |  |  | Lt Extra-Nuclear WM |  | -32.63, -31.94, 6.10 | 65535 |  |
|  |  |  | Lt Sub-Gyral WM |  | -34.22, -9.86, -8.05 | 65535 |  |
| $\mathbf{HFC}$ | **+Age** | 131265 | Rt Extra-Nuclear WM |  | 30.41, 3.31, 8.71 | 65535 |  |
|  |  |  | Rt Anterior Cingulate GM | 32 | 8.30, 40.69, 11.88 | 65535 |  |
|  |  |  | Rt Insula WM |  | 34.12, 14.30, 11.61 | 65535 |  |
|  |  | 114 |  |  | -1.26, -102.21, -7.23 | 2.56 |  |
|  |  |  |  |  | -2.94, -100.98, -19.75 | 2.29 |  |
|  | **-Age** | None |  |  |  |  |  |

Multiple regression was performed with a voxel-wise FDR-corrected significance threshold of p = 0.05 and clusters with at least 100 contiguous voxels with age and sex as covariates.

+Age and -Age indicate the positive (+) or negative (-) association with the participant's age, respectively.

There was no association between any MRI measures and MMSE scores.

**Results of ROI-based Analyses**

**Supplementary Table S3. Result of group comparison among three groups of MRI measures obtained from specific brain areas**

| **ROIs** | **Side** | **CN (0)** | **aMCI (1)** | **AD (2)** | ***Statistics** | **Robust P** | **Adj.P** |
| --- | --- | --- | --- | --- | --- | --- | --- |
| **Diffusion Interstitial Fluid (D_ISF_) *1000** | | | | | | | |
| corpus callosum | Lt | 2.81±0.45 | 2.61±0.53 | 2.39±0.56 | F=1.666, P = 0.192 | 0.107 | 0.214 |
|  | Rt | 2.61±0.49 | 2.36±0.54 | 2.14±0.57 | F=2.800, P = 0.064 | ***0.031*** | ***0.070*** |
| Cuneus | Lt | 3.56±0.32 | 3.52±0.37 | 3.44±0.33 | F=0.068, P = 0.934 | 0.946 | 0.986 |
|  | Rt | 3.44±0.38 | 3.44±0.37 | 3.34±0.37 | F=0.196, P = 0.823 | 0.832 | 0.936 |
| Hippocampus | Lt | 3.38±0.51 | 3.01±0.78 | 2.58±0.82 | ***F=5.279, P = 0.006***  ***(0,1), (0,2)*** | ***0.001*** | ***0.004*** |
|  | Rt | 3.00±0.50 | 2.76±0.69 | 2.18±0.89 | ***F=7.353, P < 0.001***  ***(0, 2), (1, 2)*** | ***0.001*** | ***0.004*** |
| Insula | Lt | 3.12±0.34 | 3.07±0.38 | 3.01±0.34 | F=0.227, P = 0.798 | 0.749 | 0.899 |
|  | Rt | 2.79±0.33 | 2.79±0.33 | 2.59±0.37 | ***F=5.366, P = 0.006***  ***(1, 2)*** | ***0.010*** | ***0.026*** |
| Middle Temporal Gyrus | Lt | 3.48±0.16 | 3.35±0.35 | 3.22±0.27 | F=2.140, P = 0.121 | ***0.004*** | ***0.012*** |
|  | Rt | 3.14±0.17 | 2.93±0.37 | 2.85±0.34 | ***F=3.525, P = 0.032***  ***(0,1), (0,2)*** | ***<0.001*** | ***0.004*** |
| Parahippocampal Gyrus | Lt | 3.26±0.20 | 3.24±0.34 | 2.98±0.35 | ***F=8.367, P < 0.001***  ***(0, 2), (1, 2)*** | ***0.003*** | ***0.010*** |
|  | Rt | 3.35±0.17 | 3.31±0.31 | 3.06±0.30 | ***F=8.396, P < 0.001***  ***(0, 2), (1, 2)*** | ***0.001*** | ***0.004*** |
| Precuneus | Lt | 3.21±0.21 | 3.15±0.20 | 3.12±0.27 | F=1.678, P = 0.190 | 0.244 | 0.415 |
|  | Rt | 3.17±0.20 | 3.16±0.22 | 3.09±0.28 | F=1.105, P = 0.334 | 0.427 | 0.641 |
| Putamen | Lt | 4.02±0.18 | 4.01±0.33 | 4.00±0.27 | F=0.022, P = 0.978 | 0.986 | 0.986 |
|  | Rt | 3.68±0.22 | 3.70±0.25 | 3.68±0.24 | F=0.285, P = 0.752 | 0.711 | 0.899 |
| Thalamus | Lt | 3.38±0.31 | 3.29±0.37 | 3.14±0.44 | F=1.243, P = 0.291 | 0.253 | 0.415 |
|  | Rt | 3.49±0.26 | 3.45±0.39 | 3.30±0.42 | F=0.395, P = 0.675 | 0.716 | 0.899 |
| **Diffusion Microvascularity (D_MV_) *1000** | | | | | | | |
| corpus callosum | Lt | 96.40±18.48 | 90.11±21.09 | 76.96±19.18 | F=2.945, P = 0.056 | ***0.040*** | ***0.071*** |
|  | Rt | 88.70±21.14 | 80.38±21.76 | 67.00±19.51 | ***F=4.601, P = 0.011***  ***(0, 2)*** | ***0.007*** | ***0.021*** |
| Cuneus | Lt | 136.46±21.27 | 137.02±21.58 | 126.18±18.17 | F=0.703, P = 0.497 | 0.553 | 0.654 |
|  | Rt | 132.21±24.45 | 133.96±21.06 | 124.30±19.66 | F=0.624, P = 0.537 | 0.581 | 0.654 |
| Hippocampus | Lt | 123.29±30.94 | 110.25±32.38 | 88.18±31.22 | ***F=5.807, P = 0.004***  ***(0, 2)*** | ***0.005*** | ***0.021*** |
|  | Rt | 97.83±23.04 | 91.93±26.13 | 66.97±28.05 | ***F=8.042, P < 0.001***  ***(0, 2), (1, 2)*** | ***0.001*** | ***0.006*** |
| Insula | Lt | 109.80±17.05 | 108.44±20.44 | 103.25±16.06 | F=0.351, P = 0.705 | 0.663 | 0.663 |
|  | Rt | 89.01±13.46 | 89.78±14.21 | 80.49±15.29 | ***F=7.731, P < 0.001***  ***(1, 2)*** | ***0.001*** | ***0.006*** |
| Middle Temporal Gyrus | Lt | 118.74±12.22 | 117.44±15.36 | 109.82±13.35 | F=1.182, P = 0.310 | 0.432 | 0.555 |
|  | Rt | 107.17±10.21 | 99.49±15.41 | 93.89±11.89 | ***F=3.693, P = 0.027***  ***(0, 2)*** | ***0.009*** | ***0.024*** |
| Parahippocampal Gyrus | Lt | 120.82±13.01 | 120.78±17.15 | 107.76±16.62 | ***F=7.501, P < 0.001***  ***(1, 2)*** | ***0.006*** | ***0.021*** |
|  | Rt | 122.12±12.31 | 121.28±15.70 | 106.83±13.81 | ***F=9.354, P < 0.001***  ***(0, 2), (1, 2)*** | ***0.001*** | ***0.006*** |
| Precuneus | Lt | 105.68±10.08 | 101.91±9.75 | 97.13±11.60 | ***F=4.121, P = 0.018*** | ***0.037*** | ***0.071*** |
|  | Rt | 101.00±8.90 | 100.39±12.29 | 95.12±10.72 | F=2.166, P = 0.118 | 0.180 | 0.269 |
| Putamen | Lt | 163.14±16.82 | 163.11±23.80 | 156.59±19.00 | F=0.559, P = 0.573 | 0.620 | 0.657 |
|  | Rt | 124.23±16.14 | 127.71±17.00 | 119.16±18.01 | F=3.008, P = 0.052 | 0.070 | 0.115 |
| Thalamus | Lt | 139.67±19.35 | 134.58±21.43 | 119.84±19.62 | ***F=4.603, P = 0.011*** | ***0.017*** | ***0.038*** |
|  | Rt | 130.73±20.97 | 129.81±24.20 | 116.58±21.69 | F=1.453, P = 0.237 | 0.281 | 0.389 |
| **Diffusion Parenchyma (D_PAR_) *1000)** | | | | | | | |
| corpus callosum | Lt | 0.44±0.05 | 0.42±0.06 | 0.41±0.08 | F=0.374, P = 0.688 | 0.574 | 0.646 |
|  | Rt | 0.42±0.06 | 0.40±0.07 | 0.39±0.08 | F=1.094, P = 0.337 | 0.230 | 0.377 |
| Cuneus | Lt | 0.62±0.04 | 0.61±0.06 | 0.63±0.05 | ***F=3.848, P = 0.023*** | 0.051 | 0.230 |
|  | Rt | 0.60±0.05 | 0.61±0.05 | 0.62±0.06 | F=2.903, P = 0.058 | 0.091 | 0.301 |
| Hippocampus | Lt | 0.65±0.09 | 0.59±0.13 | 0.55±0.16 | F=2.082, P = 0.128 | ***0.044*** | ***0.230*** |
|  | Rt | 0.63±0.10 | 0.59±0.14 | 0.50±0.19 | ***F=5.282, P = 0.006***  ***(0, 2)*** | ***0.005*** | ***0.063*** |
| Insula | Lt | 0.54±0.05 | 0.53±0.05 | 0.54±0.05 | F=0.251, P = 0.779 | 0.756 | 0.797 |
|  | Rt | 0.52±0.05 | 0.51±0.05 | 0.50±0.06 | F=1.313, P = 0.272 | 0.304 | 0.406 |
| Middle Temporal Gyrus | Lt | 0.62±0.03 | 0.61±0.05 | 0.62±0.05 | F=1.645, P = 0.196 | 0.339 | 0.406 |
|  | Rt | 0.60±0.03 | 0.57±0.06 | 0.57±0.07 | F=2.194, P = 0.115 | ***0.007*** | ***0.063*** |
| Parahippocampal Gyrus | Lt | 0.60±0.03 | 0.60±0.06 | 0.59±0.05 | F=1.566, P = 0.212 | 0.229 | 0.377 |
|  | Rt | 0.61±0.03 | 0.61±0.05 | 0.61±0.05 | F=0.149, P = 0.862 | 0.797 | 0.797 |
| Precuneus | Lt | 0.59±0.04 | 0.60±0.04 | 0.62±0.06 | F=2.041, P = 0.133 | 0.211 | 0.377 |
|  | Rt | 0.59±0.04 | 0.59±0.04 | 0.62±0.06 | F=3.042, P = 0.051 | 0.100 | 0.301 |
| Putamen | Lt | 0.47±0.06 | 0.49±0.07 | 0.53±0.08 | F=2.134, P = 0.122 | 0.176 | 0.377 |
|  | Rt | 0.58±0.05 | 0.58±0.07 | 0.63±0.08 | F=2.566, P = 0.080 | 0.157 | 0.377 |
| Thalamus | Lt | 0.51±0.05 | 0.51±0.05 | 0.54±0.07 | F=1.297, P = 0.276 | 0.298 | 0.406 |
|  | Rt | 0.56±0.04 | 0.56±0.07 | 0.58±0.06 | F=1.177, P = 0.311 | 0.326 | 0.406 |
| **Fractional Interstitial Fluid (F_ISF_)** | | | | | |  |  |
| corpus callosum | Lt | 0.13±0.02 | 0.12±0.02 | 0.12±0.03 | F=1.150, P = 0.319 | 0.189 | 0.425 |
|  | Rt | 0.12±0.02 | 0.11±0.02 | 0.11±0.03 | F=1.000, P = 0.370 | 0.240 | 0.432 |
| Cuneus | Lt | 0.15±0.01 | 0.15±0.01 | 0.15±0.02 | ***F=3.871, P = 0.023*** | ***0.044*** | ***0.158*** |
|  | Rt | 0.14±0.01 | 0.14±0.02 | 0.15±0.02 | F=0.990, P = 0.374 | 0.412 | 0.529 |
| Hippocampus | Lt | 0.15±0.03 | 0.14±0.04 | 0.13±0.04 | F=2.207, P = 0.113 | ***0.033*** | ***0.149*** |
|  | Rt | 0.14±0.03 | 0.13±0.03 | 0.12±0.05 | F=2.939, P = 0.056 | 0.053 | 0.158 |
| Insula | Lt | 0.13±0.02 | 0.13±0.02 | 0.14±0.02 | F=0.324, P = 0.724 | 0.730 | 0.756 |
|  | Rt | 0.13±0.02 | 0.13±0.02 | 0.13±0.02 | F=0.742, P = 0.478 | 0.528 | 0.634 |
| Middle Temporal Gyrus | Lt | 0.14±0.01 | 0.14±0.01 | 0.14±0.01 | F=1.651, P = 0.195 | 0.237 | 0.432 |
|  | Rt | 0.13±0.01 | 0.13±0.02 | 0.13±0.01 | ***F=4.142, P = 0.018***  ***(0,1)*** | ***0.005*** | ***0.047*** |
| Parahippocampal Gyrus | Lt | 0.14±0.01 | 0.15±0.02 | 0.15±0.02 | F=1.596, P = 0.206 | 0.276 | 0.452 |
|  | Rt | 0.15±0.01 | 0.15±0.01 | 0.15±0.02 | F=0.421, P = 0.657 | 0.756 | 0.756 |
| Precuneus | Lt | 0.13±0.01 | 0.13±0.01 | 0.14±0.01 | F=0.535, P = 0.587 | 0.672 | 0.756 |
|  | Rt | 0.13±0.01 | 0.13±0.01 | 0.13±0.01 | F=1.354, P = 0.261 | 0.383 | 0.529 |
| Putamen | Lt | 0.12±0.02 | 0.14±0.03 | 0.16±0.04 | ***F=5.362, P = 0.006***  ***(0, 2)*** | ***0.005*** | ***0.047*** |
|  | Rt | 0.15±0.02 | 0.15±0.03 | 0.17±0.03 | ***F=5.422, P = 0.005***  ***(1, 2)*** | ***0.013*** | ***0.080*** |
| Thalamus | Lt | 0.12±0.02 | 0.13±0.02 | 0.14±0.03 | F=2.329, P = 0.101 | 0.086 | 0.220 |
|  | Rt | 0.14±0.02 | 0.15±0.02 | 0.15±0.03 | F=0.967, P = 0.383 | 0.346 | 0.519 |
| **Fractional Microvascularity (F_MV_)** | | | | | | | |
| corpus callosum | Lt | 0.04±0.01 | 0.04±0.01 | 0.03±0.01 | F=0.247, P = 0.781 | 0.727 | 0.818 |
|  | Rt | 0.03±0.01 | 0.03±0.01 | 0.03±0.01 | F=1.005, P = 0.368 | 0.363 | 0.550 |
| Cuneus | Lt | 0.05±0.01 | 0.05±0.01 | 0.05±0.01 | F=1.017, P = 0.364 | 0.341 | 0.550 |
|  | Rt | 0.05±0.01 | 0.05±0.01 | 0.05±0.01 | F=1.486, P = 0.229 | 0.259 | 0.518 |
| Hippocampus | Lt | 0.05±0.01 | 0.05±0.02 | 0.04±0.02 | F=1.425, P = 0.244 | 0.207 | 0.467 |
|  | Rt | 0.03±0.01 | 0.04±0.01 | 0.03±0.02 | F=1.967, P = 0.143 | 0.148 | 0.467 |
| Insula | Lt | 0.05±0.01 | 0.05±0.01 | 0.05±0.01 | F=0.096, P = 0.909 | 0.908 | 0.908 |
|  | Rt | 0.04±0.01 | 0.04±0.01 | 0.04±0.01 | ***F=3.562, P = 0.031*** | ***0.032*** | ***0.223*** |
| Middle Temporal Gyrus | Lt | 0.04±0.01 | 0.04±0.01 | 0.04±0.01 | F=1.101, P = 0.335 | 0.395 | 0.550 |
|  | Rt | 0.03±0.00 | 0.03±0.01 | 0.03±0.01 | F=0.262, P = 0.770 | 0.787 | 0.834 |
| Parahippocampal Gyrus | Lt | 0.05±0.01 | 0.06±0.01 | 0.05±0.01 | ***F=3.122, P = 0.047*** | 0.084 | 0.379 |
|  | Rt | 0.05±0.01 | 0.05±0.01 | 0.05±0.01 | F=2.233, P = 0.111 | 0.168 | 0.467 |
| Precuneus | Lt | 0.03±0.01 | 0.03±0.01 | 0.03±0.01 | F=1.067, P = 0.347 | 0.397 | 0.550 |
|  | Rt | 0.03±0.01 | 0.03±0.01 | 0.03±0.01 | F=0.780, P = 0.460 | 0.480 | 0.617 |
| Putamen | Lt | 0.06±0.01 | 0.07±0.01 | 0.06±0.01 | F=0.684, P = 0.506 | 0.517 | 0.621 |
|  | Rt | 0.04±0.01 | 0.04±0.01 | 0.04±0.01 | F=1.918, P = 0.150 | 0.194 | 0.467 |
| Thalamus | Lt | 0.06±0.01 | 0.06±0.01 | 0.05±0.01 | ***F=5.039, P = 0.008***  ***(1, 2)*** | ***0.013*** | ***0.223*** |
|  | Rt | 0.05±0.01 | 0.05±0.01 | 0.05±0.01 | ***F=3.528, P = 0.032*** | ***0.037*** | ***0.223*** |
| **Fractional Parenchyma (F_PAR_)** | | | | | | | |
| corpus callosum | Lt | 0.54±0.09 | 0.50±0.11 | 0.44±0.11 | F=2.769, P = 0.066 | ***0.045*** | ***0.073*** |
|  | Rt | 0.50±0.10 | 0.45±0.11 | 0.39±0.11 | ***F=4.004, P = 0.020***  ***(0, 2)*** | ***0.009*** | ***0.025*** |
| Cuneus | Lt | 0.68±0.07 | 0.66±0.07 | 0.63±0.06 | F=0.441, P = 0.644 | 0.659 | 0.698 |
|  | Rt | 0.66±0.07 | 0.66±0.07 | 0.63±0.06 | F=0.038, P = 0.962 | 0.970 | 0.970 |
| Hippocampus | Lt | 0.64±0.11 | 0.57±0.17 | 0.47±0.15 | ***F=5.375, P = 0.006***  ***(0, 2)*** | ***0.001*** | ***0.003*** |
|  | Rt | 0.64±0.12 | 0.57±0.16 | 0.42±0.17 | ***F=10.828, P < 0.001***  ***(0, 2), (1, 2)*** | ***<0.001*** | ***<0.001*** |
| Insula | Lt | 0.61±0.06 | 0.60±0.07 | 0.56±0.07 | F=1.623, P = 0.201 | 0.173 | 0.223 |
|  | Rt | 0.56±0.06 | 0.55±0.07 | 0.50±0.08 | ***F=5.069, P = 0.007***  ***(1, 2)*** | ***0.010*** | ***0.025*** |
| Middle Temporal Gyrus | Lt | 0.70±0.04 | 0.67±0.08 | 0.64±0.06 | F=1.755, P = 0.176 | ***0.020*** | ***0.039*** |
|  | Rt | 0.65±0.05 | 0.61±0.08 | 0.57±0.08 | ***F=3.445, P = 0.034***  ***(0,1), (0, 2)*** | ***0.001*** | ***0.003*** |
| Parahippocampal Gyrus | Lt | 0.63±0.04 | 0.60±0.07 | 0.53±0.06 | ***F=13.819, P < 0.001***  ***(0, 2), (1, 2)*** | ***<0.001*** | ***<0.001*** |
|  | Rt | 0.67±0.04 | 0.65±0.07 | 0.58±0.06 | ***F=12.538, P < 0.001***  ***(0, 2), (1, 2)*** | ***<0.001*** | ***<0.001*** |
| Precuneus | Lt | 0.69±0.05 | 0.67±0.05 | 0.66±0.06 | F=0.679, P = 0.509 | 0.538 | 0.605 |
|  | Rt | 0.69±0.05 | 0.69±0.05 | 0.66±0.06 | F=1.246, P = 0.290 | 0.393 | 0.471 |
| Putamen | Lt | 0.80±0.04 | 0.77±0.06 | 0.76±0.06 | F=2.895, P = 0.058 | ***0.012*** | ***0.026*** |
|  | Rt | 0.81±0.03 | 0.80±0.05 | 0.78±0.04 | F=1.688, P = 0.188 | ***0.044*** | ***0.073*** |
| Thalamus | Lt | 0.66±0.07 | 0.63±0.09 | 0.60±0.08 | F=1.753, P = 0.177 | 0.085 | 0.127 |
|  | Rt | 0.71±0.06 | 0.68±0.08 | 0.65±0.08 | F=1.106, P = 0.334 | 0.128 | 0.177 |
| **High Frequency Conductivity (HFC)** | | | | | | | |
| corpus callosum | Lt | 0.78±0.13 | 0.83±0.17 | 0.92±0.19 | F=2.614, P = 0.076 | 0.063 | 0.162 |
|  | Rt | 0.79±0.12 | 0.83±0.15 | 0.93±0.17 | ***F=3.462, P = 0.034*** | ***0.034*** | ***0.162*** |
| Cuneus | Lt | 1.00±0.16 | 1.01±0.17 | 1.02±0.17 | F=0.590, P = 0.555 | 0.582 | 0.655 |
|  | Rt | 0.96±0.17 | 0.96±0.18 | 0.98±0.19 | F=1.282, P = 0.281 | 0.275 | 0.495 |
| Hippocampus | Lt | 0.78±0.16 | 0.77±0.17 | 0.81±0.18 | F=0.113, P = 0.893 | 0.890 | 0.890 |
|  | Rt | 0.73±0.20 | 0.78±0.18 | 0.76±0.20 | F=0.782, P = 0.459 | 0.443 | 0.586 |
| Insula | Lt | 0.76±0.10 | 0.77±0.12 | 0.86±0.12 | F=2.922, P = 0.057 | 0.059 | 0.162 |
|  | Rt | 0.71±0.11 | 0.73±0.11 | 0.81±0.13 | F=3.058, P = 0.050 | 0.057 | 0.162 |
| Middle Temporal Gyrus | Lt | 0.91±0.10 | 0.87±0.14 | 0.93±0.15 | F=2.570 P = 0.080 | 0.082 | 0.185 |
|  | Rt | 0.81±0.10 | 0.79±0.13 | 0.82±0.16 | F=0.552, P = 0.577 | 0.516 | 0.619 |
| Parahippocampal Gyrus | Lt | 0.74±0.10 | 0.75±0.12 | 0.81±0.13 | F=1.017, P = 0.364 | 0.448 | 0.586 |
|  | Rt | 0.73±0.12 | 0.77±0.010 | 0.79±0.12 | F=0.868, P = 0.422 | 0.456 | 0.586 |
| Precuneus | Lt | 1.07±0.10 | 1.08±0.12 | 1.10±0.15 | F=0.097, P = 0.907 | 0.883 | 0.890 |
|  | Rt | 1.07±0.08 | 1.08±0.12 | 1.13±0.14 | ***F=3.163, P = 0.045*** | ***0.049*** | ***0.162*** |
| Putamen | Lt | 0.77±0.17 | 0.74±0.15 | 0.80±0.17 | F=0.925, P = 0.399 | 0.453 | 0.586 |
|  | Rt | 0.77±0.15 | 0.75±0.16 | 0.84±0.17 | F=1.796, P = 0.169 | 0.158 | 0.315 |
| Thalamus | Lt | 0.71±0.12 | 0.76±0.14 | 0.82±0.13 | F=2.841, P = 0.061 | ***0.034*** | ***0.162*** |
|  | Rt | 0.76±0.15 | 0.82±0.17 | 0.90±0.14 | ***F=3.260, P = 0.041***  ***(0, 2)*** | ***0.031*** | ***0.162*** |

Data are presented as the mean ± standard deviation.

Statistical results show ANCOVA with age and sex as covariates. When the main effect of Group was significant, pairwise comparisons were conducted with Bonferroni correction (two-tailed, α=0.05), showing in parentheses as (0:1), (0:2), and (1:2).

Robust p values were obtained from HC3-based heteroscedasticity-robust ANCOVA.

Adjusted p values were calculated using the Benjamini–Hochberg procedure applied to the robust p values within each MRI metric across bilateral ROI tests.

**Supplementary Table S4. Result of correlation analysis between MRI measures and age or Mini-Mental State Examination (MMSE) scores using all participant data**

| **ROIs** | **side** | **Age*(r, p)** | **MMSE† (r, p)** |
| --- | --- | --- | --- |
| **Diffusion Interstitial Fluid (D_ISF_) *1000** | | | |
| corpus callosum | Lt | **r = -0.450, P < 0.001, Adj P < 0.001** | r = 0.141, P = 0.074, Adj P = 0.190 |
|  | Rt | **r = -0.423, P < 0.001, Adj P < 0.001** | r = 0.134, P = 0.089, Adj P = 0.200 |
| Cuneus | Lt | **r = -0.471, P < 0.001, Adj P < 0.001** | r = 0.049, P = 0.539, Adj P = 0.647 |
|  | Rt | **r = -0.441, P < 0.001, Adj P < 0.001** | r = 0.049, P = 0.533, Adj P = 0.647 |
| Hippocampus | Lt | **r = -0.354, P < 0.001, Adj P < 0.001** | **r = 0.217, P = 0.006, Adj P = 0.027** |
|  | Rt | **r = -0.365, P < 0.001, Adj P < 0.001** | **r = 0.221, P = 0.005, Adj P = 0.027** |
| Insula | Lt | **r = -0.192, P = 0.015, Adj P = 0.017** | r = 0.082, P = 0.304, Adj P = 0.497 |
|  | Rt | **r = -0.327, P < 0.001, Adj P < 0.001** | **r = 0.195, P = 0.013, Adj P = 0.047** |
| Middle Temporal Gyrus | Lt | **r = -0.451, P < 0.001, Adj P < 0.001** | **r = 0.166, P = 0.036, Adj P = 0.108** |
|  | Rt | **r = -0.381, P < 0.001, Adj P < 0.001** | r = 0.050, P = 0.526, Adj P = 0.647 |
| Parahippocampal Gyrus | Lt | **r = -0.265, P = 0.001, Adj P = 0.001** | **r = 0.318, P < 0.001, Adj P = 0.002** |
|  | Rt | **r = -0.305, P < 0.001, Adj P < 0.001** | **r = 0.297, P = 0.001, Adj P = 0.009** |
| Precuneus | Lt | r = -0.126, P = 0.112, Adj P = 0.119 | r = 0.092, P = 0.244, Adj P = 0.488 |
|  | Rt | **r = -0.220, P = 0.005, Adj P = 0.006** | r = 0.038, P = 0.633, Adj P = 0.712 |
| Putamen | Lt | r = -0.084, P = 0.292, Adj P = 0.292 | r = 0.025, P = 0.757, Adj P = 0.802 |
|  | Rt | **r = -0.198, P = 0.012, Adj P = 0.014** | r = 0.007, P = 0.926, Adj P = 0.926 |
| Thalamus | Lt | **r = -0.315, P < 0.001, Adj P < 0.001** | r = 0.077, P = 0.331, Adj P = 0.497 |
|  | Rt | **r = -0.373, P < 0.001, Adj P < 0.001** | r = 0.082, P = 0.300, Adj P = 0.497 |
| **Diffusion Microvascularity (D_MV_) *1000** | | | |
| corpus callosum | Lt | **r = -0.538, P < 0.001, Adj P < 0.001** | **r = 0.176, P = 0.025, Adj P = 0.063** |
|  | Rt | **r = -0.522, P < 0.001, Adj P < 0.001** | r = 0.153, P = 0.053, Adj P = 0.088 |
| Cuneus | Lt | **r = -0.430, P < 0.001, Adj P < 0.001** | **r = 0.159, P = 0.043, Adj P = 0.086** |
|  | Rt | **r = -0.396, P < 0.001, Adj P < 0.001** | r = 0.126, P = 0.111, Adj P = 0.134 |
| Hippocampus | Lt | **r = -0.382, P < 0.001, Adj P < 0.001** | **r = 0.239, P = 0.002, Adj P = 0.012** |
|  | Rt | **r = -0.473, P < 0.001, Adj P < 0.001** | **r = 0.233, P = 0.003, Adj P = 0.013** |
| Insula | Lt | r = -0.215, P = 0.006, Adj P = 0.006 | r = 0.116, P = 0.142, Adj P = 0.160 |
|  | Rt | **r = -0.394, P < 0.001, Adj P < 0.001** | **r = 0.212, P = 0.007, Adj P = 0.024** |
| Middle Temporal Gyrus | Lt | **r = -0.425, P < 0.001, Adj P < 0.001** | r = 0.152, P = 0.054, Adj P = 0.088 |
|  | Rt | **r = -0.432, P < 0.001, Adj P < 0.001** | r = 0.142, P = 0.073, Adj P = 0.101 |
| Parahippocampal Gyrus | Lt | **r = -0.314, P < 0.001, Adj P < 0.001** | **r = 0.308, P < 0.001, Adj P < 0.001** |
|  | Rt | **r = -0.397, P < 0.001, Adj P < 0.001** | **r = 0.328, P < 0.001, Adj P < 0.001** |
| Precuneus | Lt | **r = -0.237, P = 0.003, Adj P = 0.003** | **r = 0.209, P = 0.008, Adj P = 0.024** |
|  | Rt | **r = -0.242, P = 0.002, Adj P = 0.002** | r = 0.147, P = 0.063, Adj P = 0.094 |
| Putamen | Lt | r = -0.208, P = 0.008, Adj P = 0.008 | r = 0.101, P = 0.202, Adj P = 0.202 |
|  | Rt | **r = -0.370, P < 0.001, Adj P < 0.001** | r = 0.126, P = 0.112, Adj P = 0.134 |
| Thalamus | Lt | **r = -0.436, P < 0.001, Adj P < 0.001** | **r = 0.173, P = 0.028, Adj P = 0.063** |
|  | Rt | **r = -0.543, P < 0.001, Adj P < 0.001** | r = 0.113, P = 0.154, Adj P = 0.163 |
| **Diffusion Parenchyma (D_PAR_) *1000** | | | |
| corpus callosum | Lt | **r = -0.157, P = 0.047, Adj P = 0.085** | r = 0.058, P = 0.464, Adj P = 0.597 |
|  | Rt | r = -0.127, P = 0.111, Adj P = 0.167 | r = 0.026, P = 0.748, Adj P = 0.748 |
| Cuneus | Lt | r = -0.086, P = 0.278, Adj P = 0.334 | r = -0.114, P = 0.149, Adj P = 0.260 |
|  | Rt | **r = -0.176, P = 0.026, Adj P = 0.052** | r = -0.112, P = 0.159, Adj P = 0.260 |
| Hippocampus | Lt | **r = -0.203, P = 0.010, Adj P = 0.026** | **r = 0.159, P = 0.045, Adj P = 0.162** |
|  | Rt | **r = -0.238, P = 0.002, Adj P = 0.006** | **r = 0.188, P = 0.017, Adj P = 0.140** |
| Insula | Lt | r = 0.077, P = 0.332, Adj P = 0.352 | r = 0.030, P = 0.706, Adj P = 0.748 |
|  | Rt | r = -0.031, P = 0.693, Adj P = 0.693 | r = 0.063, P = 0.430, Adj P = 0.595 |
| Middle Temporal Gyrus | Lt | r = -0.082, P = 0.302, Adj P = 0.340 | r = -0.046, P = 0.562, Adj P = 0.632 |
|  | Rt | r = -0.096, P = 0.229, Adj P = 0.294 | r = -0.047, P = 0.550, Adj P = 0.632 |
| Parahippocampal Gyrus | Lt | r = 0.115, P = 0.149, Adj P = 0.206 | **r = 0.174, P = 0.027, Adj P = 0.140** |
|  | Rt | r = 0.128, P = 0.106, Adj P = 0.167 | r = 0.065, P = 0.415, Adj P = 0.595 |
| Precuneus | Lt | **r = 0.293, P = 0.001, Adj P = 0.004** | r = -0.118, P = 0.136, Adj P = 0.260 |
|  | Rt | **r = 0.176, P = 0.026, Adj P = 0.052** | **r = -0.170, P = 0.031, Adj P = 0.140** |
| Putamen | Lt | **r = 0.382, P < 0.001, Adj P < 0.001** | **r = -0.203, P = 0.010, Adj P = 0.140** |
|  | Rt | **r = 0.445, P < 0.001, Adj P < 0.001** | r = -0.134, P = 0.089, Adj P = 0.247 |
| Thalamus | Lt | **r = 0.290, P = 0.001, Adj P = 0.004** | r = -0.131, P = 0.096, Adj P = 0.247 |
|  | Rt | **r = 0.295, P = 0.001, Adj P = 0.004** | r = -0.113, P = 0.152, Adj P = 0.260 |
| **Fractional Interstitial Fluid (F_ISF_)** | | | |
| corpus callosum | Lt | r = -0.141, P = 0.075, Adj P = 0.131 | r = 0.058, P = 0.466, Adj P = 0.699 |
|  | Rt | r = -0.116, P = 0.143, Adj P = 0.214 | r = 0.032, P = 0.685, Adj P = 0.818 |
| Cuneus | Lt | r = -0.059, P = 0.456, Adj P = 0.483 | r = -0.076, P = 0.340, Adj P = 0.556 |
|  | Rt | r = -0.071, P = 0.374, Adj P = 0.449 | r = -0.030, P = 0.707, Adj P = 0.818 |
| Hippocampus | Lt | r = -0.088, P = 0.268, Adj P = 0.345 | r = 0.123, P = 0.120, Adj P = 0.270 |
|  | Rt | r = -0.109, P = 0.170, Adj P = 0.235 | **r = 0.159, P = 0.043, Adj P = 0.242** |
| Insula | Lt | **r = 0.300, P = 0.001, Adj P = 0.002** | r = -0.022, P = 0.781, Adj P = 0.827 |
|  | Rt | r = 0.139, P = 0.080, Adj P = 0.131 | r = 0.118, P = 0.137, Adj P = 0.274 |
| Middle Temporal Gyrus | Lt | r = 0.065, P = 0.411, Adj P = 0.462 | r = 0.028, P = 0.727, Adj P = 0.818 |
|  | Rt | r = 0.046, P = 0.560, Adj P = 0.560 | r = -0.048, P = 0.546, Adj P = 0.756 |
| Parahippocampal Gyrus | Lt | **r = 0.319, P = 0.001, Adj P = 0.002** | r = 0.144, P = 0.068, Adj P = 0.242 |
|  | Rt | **r = 0.356, P = 0.001, Adj P = 0.002** | r = 0.132, P = 0.094, Adj P = 0.242 |
| Precuneus | Lt | **r = 0.341, P = 0.001, Adj P = 0.002** | r = -0.015, P = 0.855, Adj P = 0.855 |
|  | Rt | **r = 0.245, P = 0.002, Adj P = 0.004** | r = -0.135, P = 0.089, Adj P = 0.242 |
| Putamen | Lt | **r = 0.418, P = 0.001, Adj P = 0.002** | **r = -0.227, P = 0.004, Adj P = 0.072** |
|  | Rt | **r = 0.426, P = 0.001, Adj P = 0.002** | **r = -0.180, P = 0.022, Adj P = 0.198** |
| Thalamus | Lt | **r = 0.349, P = 0.001, Adj P = 0.002** | r = -0.144, P = 0.069, Adj P = 0.242 |
|  | Rt | **r = 0.303, P = 0.001, Adj P = 0.002** | r = -0.097, P = 0.222, Adj P = 0.400 |
| **Fractional Microvascularity (F_MV_)** | | | |
| corpus callosum | Lt | **r = -0.294, P = 0.001, Adj P = 0.004** | r = 0.098, P = 0.217, Adj P = 0.412 |
|  | Rt | **r = -0.276, P = 0.001, Adj P = 0.004** | r = 0.079, P = 0.321, Adj P = 0.444 |
| Cuneus | Lt | **r = -0.180, P = 0.023, Adj P = 0.059** | r = 0.079, P = 0.317, Adj P = 0.444 |
|  | Rt | **r = -0.124, P = 0.117, Adj P = 0.234** | r = 0.045, P = 0.569, Adj P = 0.640 |
| Hippocampus | Lt | **r = -0.209, P = 0.008, Adj P = 0.029** | r = 0.137, P = 0.083, Adj P = 0.249 |
|  | Rt | **r = -0.102, P = 0.200, Adj P = 0.300** | r = 0.095, P = 0.229, Adj P = 0.412 |
| Insula | Lt | r = 0.148, P = 0.062, Adj P = 0.140 | r = 0.081, P = 0.309, Adj P = 0.444 |
|  | Rt | r = 0.036, P = 0.654, Adj P = 0.692 | r = 0.168, P = 0.033, Adj P = 0.149 |
| Middle Temporal Gyrus | Lt | r = -0.031, P = 0.701, Adj P = 0.701 | r = 0.122, P = 0.122, Adj P = 0.274 |
|  | Rt | r = -0.062, P = 0.435, Adj P = 0.522 | r = -0.065, P = 0.409, Adj P = 0.526 |
| Parahippocampal Gyrus | Lt | r = 0.081, P = 0.308, Adj P = 0.396 | **r = 0.210, P = 0.008, Adj P = 0.144** |
|  | Rt | r = 0.037, P = 0.644, Adj P = 0.692 | **r = 0.175, P = 0.026, Adj P = 0.149** |
| Precuneus | Lt | r = 0.120, P = 0.131, Adj P = 0.236 | **r = 0.123, P = 0.119, Adj P = 0.274** |
|  | Rt | r = 0.091, P = 0.250, Adj P = 0.346 | r = 0.023, P = 0.773, Adj P = 0.818 |
| Putamen | Lt | r = -0.105, P = 0.185, Adj P = 0.300 | r = 0.061, P = 0.443, Adj P = 0.532 |
|  | Rt | **r = -0.193, P = 0.015, Adj P = 0.045** | r = 0.016, P = 0.840, Adj P = 0.840 |
| Thalamus | Lt | **r = -0.275, P = 0.001, Adj P = 0.004** | **r = 0.185, P = 0.019, Adj P = 0.149** |
|  | Rt | **r = -0.350, P = 0.001, Adj P = 0.004** | r = 0.139, P = 0.078, Adj P = 0.249 |
| **Fractional Parenchyma (F_PAR_)** | | | |
| corpus callosum | Lt | **r = -0.539, P = 0.001, Adj P = 0.001** | **r = 0.173, P = 0.028, Adj P = 0.101** |
|  | Rt | **r = -0.512, P = 0.001, Adj P = 0.001** | **r = 0.167, P = 0.034, Adj P = 0.102** |
| Cuneus | Lt | **r = -0.505, P = 0.001, Adj P = 0.001** | r = 0.082, P = 0.302, Adj P = 0.362 |
|  | Rt | **r = -0.537, P = 0.001, Adj P = 0.001** | r = 0.046, P = 0.560, Adj P = 0.560 |
| Hippocampus | Lt | **r = -0.423, P = 0.001, Adj P = 0.001** | **r = 0.241, P = 0.002, Adj P = 0.009** |
|  | Rt | **r = -0.455, P = 0.001, Adj P = 0.001** | **r = 0.261, P = 0.001, Adj P = 0.006** |
| Insula | Lt | **r = -0.455, P = 0.001, Adj P = 0.001** | r = 0.133, P = 0.093, Adj P = 0.169 |
|  | Rt | **r = -0.508, P = 0.001, Adj P = 0.001** | r = 0.132, P = 0.094, Adj P = 0.169 |
| Middle Temporal Gyrus | Lt | **r = -0.529, P = 0.001, Adj P = 0.001** | r = 0.140, P = 0.076, Adj P = 0.169 |
|  | Rt | **r = -0.472, P = 0.001, Adj P = 0.001** | **r = 0.093, P = 0.239, Adj P = 0.315** |
| Parahippocampal Gyrus | Lt | **r = -0.553, P = 0.001, Adj P = 0.001** | **r = 0.365, P = 0.001, Adj P = 0.006** |
|  | Rt | **r = -0.540, P = 0.001, Adj P = 0.001** | **r = 0.294, P = 0.001, Adj P = 0.006** |
| Precuneus | Lt | **r = -0.297, P = 0.001, Adj P = 0.001** | r = 0.055, P = 0.492, Adj P = 0.521 |
|  | Rt | **r = -0.383, P = 0.001, Adj P = 0.001** | r = 0.092, P = 0.245, Adj P = 0.315 |
| Putamen | Lt | **r = -0.248, P = 0.002, Adj P = 0.002** | r = 0.148, P = 0.061, Adj P = 0.157 |
|  | Rt | **r = -0.311, P = 0.001, Adj P = 0.001** | r = 0.096, P = 0.227, Adj P = 0.315 |
| Thalamus | Lt | **r = -0.473, P = 0.001, Adj P = 0.001** | r = 0.092, P = 0.244, Adj P = 0.315 |
|  | Rt | **r = -0.490, P = 0.001, Adj P = 0.001** | r = 0.070, P = 0.376, Adj P = 0.423 |
| **High Frequency Conductivity (HFC)** | | | |
| corpus callosum | Lt | **r = 0.447, P = 0.001, Adj P = 0.002** | r = -0.121, P = 0.127, Adj P = 0.416 |
|  | Rt | **r = 0.451, P = 0.001, Adj P = 0.002** | r = -0.109, P = 0.169, Adj P = 0.416 |
| Cuneus | Lt | r = -0.106, P = 0.182, Adj P = 0.218 | r = -0.094, P = 0.241, Adj P = 0.434 |
|  | Rt | r = -0.086, P = 0.282, Adj P = 0.299 | r = -0.062, P = 0.439, Adj P = 0.564 |
| Hippocampus | Lt | **r = 0.223, P = 0.005, Adj P = 0.008** | r = -0.009, P = 0.910, Adj P = 0.913 |
|  | Rt | r = 0.082, P = 0.303, Adj P = 0.303 | r = 0.067, P = 0.403, Adj P = 0.559 |
| Insula | Lt | **r = 0.518, P = 0.001, Adj P = 0.002** | r = -0.116, P = 0.145, Adj P = 0.416 |
|  | Rt | **r = 0.530, P = 0.001, Adj P = 0.002** | r = -0.106, P = 0.185, Adj P = 0.416 |
| Middle Temporal Gyrus | Lt | r = 0.138, P = 0.083, Adj P = 0.107 | r = -0.077, P = 0.334, Adj P = 0.547 |
|  | Rt | r = 0.149, P = 0.061, Adj P = 0.084 | r = 0.009, P = 0.913, Adj P = 0.913 |
| Parahippocampal Gyrus | Lt | **r = 0.367, P = 0.001, Adj P = 0.002** | r = -0.122, P = 0.126, Adj P = 0.416 |
|  | Rt | **r = 0.248, P = 0.002, Adj P = 0.004** | r = -0.047, P = 0.558, Adj P = 0.670 |
| Precuneus | Lt | r = 0.151, P = 0.058, Adj P = 0.084 | r = -0.097, P = 0.225, Adj P = 0.434 |
|  | Rt | r = 0.099, P = 0.215, Adj P = 0.242 | **r = -0.234, P = 0.003, Adj P = 0.054** |
| Putamen | Lt | **r = 0.292, P = 0.001, Adj P = 0.002** | r = -0.015, P = 0.853, Adj P = 0.913 |
|  | Rt | **r = 0.379, P = 0.001, Adj P = 0.002** | r = -0.067, P = 0.404, Adj P = 0.559 |
| Thalamus | Lt | **r = 0.440, P = 0.001, Adj P = 0.002** | **r = -0.196, P = 0.013, Adj P = 0.108** |
|  | Rt | **r = 0.488, P = 0.001, Adj P = 0.002** | **r = -0.188, P = 0.018, Adj P = 0.108** |

Age* (r, p): Partial Pearson’s correlation coefficient (r) and P-value between age and each MRI-derived index, adjusting for sex.
MMSE† (r, p): Partial Pearson’s correlation coefficient (r) and P-value between MMSE and each MRI-derived index, adjusting for both age and sex.
Adjusted P values were calculated using the Benjamini–Hochberg procedure within each predictor–metric family across bilateral ROI tests.

Specifically, multiplicity correction was applied separately for Age–D_ISF_, Age–D_MV_, Age–D_PAR_, Age–F_ISF_, Age–F_MV_, Age–F_PAR_, Age–HFC, MMSE–D_ISF_, MMSE–D_MV_, MMSE–D_PAR_, MMSE–F_ISF_, MMSE–F_MV_, MMSE–F_PAR_, and MMSE–HFC.

P-values between 0.0001 and 0.001 are denoted as P < 0.001.
P-values less than 0.0001 are denoted as P < 0.001*

**Supplementary Table S5. Adjusted associations between high-frequency conductivity (HFC) and diffusion or fractional interstitial fluid measures (D_ISF_, F_ISF_), controlling for diagnostic group, age, and sex.**

| **ROIs** | **side** | **HFC vs D_ISF_ (adjusted r, p)** | **HFC vs F_ISF_ (adjusted r, p)** |
| --- | --- | --- | --- |
| corpus callosum | Lt | **r = -0.722, P < 0.001*, Adj P < 0.001*** | **r = -0.633, P < 0.001*, Adj P < 0.001*** |
|  | Rt | **r = -0.648, P < 0.001*, Adj P < 0.001*** | **r = -0.553, P < 0.001*, Adj P < 0.001*** |
| Cuneus | Lt | r = 0.077, P = 0.342, Adj P = 0.440 | r = 0.095, P = 0.238, Adj P = 0.575 |
|  | Rt | r = -0.026, P = 0.747, Adj P = 0.791 | r = -0.022, P = 0.784, Adj P = 0.941 |
| Hippocampus | Lt | r = 0.088, P = 0.274, Adj P = 0.379 | r = 0.008, P = 0.924, Adj P = 0.973 |
|  | Rt | r = -0.063, P = 0.433, Adj P = 0.520 | r = -0.035, P = 0.662, Adj P = 0.883 |
| Insula | Lt | r = -0.096, P = 0.237, Adj P = 0.366 | r = 0.054, P = 0.501, Adj P = 0.819 |
|  | Rt | r = -0.138, P = 0.087, Adj P = 0.223 | r = -0.062, P = 0.445, Adj P = 0.802 |
| Middle Temporal Gyrus | Lt | r = 0.094, P = 0.244, Adj P = 0.366 | r = -0.011, P = 0.890, Adj P = 0.973 |
|  | Rt | r = 0.110, P = 0.172, Adj P = 0.342 | r = 0.100, P = 0.215, Adj P = 0.575 |
| Parahippocampal Gyrus | Lt | **r = -0.199, P = 0.013, Adj P = 0.046** | r = -0.085, P = 0.294, Adj P = 0.588 |
|  | Rt | **r = -0.184, P = 0.022, Adj P = 0.066** | r = -0.113, P = 0.161, Adj P = 0.575 |
| Precuneus | Lt | r = -0.005, P = 0.950, Adj P = 0.950 | r = -0.003, P = 0.973, Adj P = 0.973 |
|  | Rt | r = -0.115, P = 0.152, Adj P = 0.342 | r = -0.033, P = 0.686, Adj P = 0.883 |
| Putamen | Lt | r = 0.106, P = 0.190, Adj P = 0.342 | r = 0.092, P = 0.256, Adj P = 0.575 |
|  | Rt | r = -0.040, P = 0.622, Adj P = 0.700 | r = -0.046, P = 0.571, Adj P = 0.856 |
| Thalamus | Lt | **r = -0.350, P < 0.001*, Adj P < 0.001*** | r = -0.102, P = 0.207, Adj P = 0.575 |
|  | Rt | **r = -0.405, P < 0.001*, Adj P < 0.001*** | r = -0.151, P = 0.061, Adj P = 0.368 |

HFC vs D_ISF_ and HFC vs F_ISF_: partial correlation coefficients derived from linear models adjusting for diagnostic group, age, and sex.

Adjusted P values were calculated using the Benjamini–Hochberg procedure separately for HFC–D_ISF_ and HFC–F_ISF_ associations across bilateral ROI tests.

P-values between 0.0001 and 0.001 are denoted as P < 0.001.
P-values less than 0.0001 are denoted as P < 0.001*.

**Supplementary Table S6. Group-stratified adjusted associations between high-frequency conductivity (HFC) and diffusion or fractional interstitial fluid measures (D_ISF_, F_ISF_), with tests for interaction by diagnostic group.**

| **ROI** | **side** | **CN** | **MCI** | **AD** | **interaction P** | **Interaction Adj. P** |
| --- | --- | --- | --- | --- | --- | --- |
| **HFC vs D_ISF_** | | | | | | |
| corpus callosum | Lt | **r = 0.543,** **P = 0.006** Adj P = 0.109 | **r = 0.726,** **P < 0.001** **Adj P < 0.001** | **r = 0.757,** **P < 0.001** **Adj P < 0.001** | 0.342 | 0.560 |
| corpus callosum | Rt | **r = -0.446,** **P = 0.029** Adj P = 0.142 | **r = -0.685,** **P < 0.001** **Adj P < 0.001** | **r = -0.674,** **P < 0.001** **Adj P < 0.001** | 0.313 | 0.560 |
| Cuneus | Lt | **r = 0.436,** **P = 0.033** Adj P = 0.142 | r = -0.099, P = 0.399 Adj P = 0.552 | r = 0.225, P = 0.102 Adj P = 0.204 | 0.105 | 0.454 |
| Cuneus | Rt | r = 0.029, P = 0.894 Adj P = 0.955 | r = 0.047, P = 0.691 Adj P = 0.732 | r = -0.096, P = 0.488 Adj P = 0.731 | 0.477 | 0.673 |
| Hippocampus | Lt | r = 0.065, P = 0.763 Adj P = 0.915 | r = 0.120, P = 0.303 Adj P = 0.519 | r = 0.063, P = 0.649 Adj P = 0.777 | 0.923 | 0.923 |
| Hippocampus | Rt | r = 0.203, P = 0.342 Adj P = 0.686 | r = -0.065, P = 0.582 Adj P = 0.655 | r = -0.142, P = 0.304 Adj P = 0.548 | 0.523 | 0.673 |
| Insula | Lt | r = -0.170, P = 0.427 Adj P = 0.745 | r = -0.215, P = 0.064 Adj P = 0.232 | r = 0.070, P = 0.613 Adj P = 0.777 | 0.336 | 0.560 |
| Insula | Rt | **r = -0.406,** **P = 0.049** Adj P = 0.147 | r = -0.134, P = 0.250 Adj P = 0.500 | r = -0.055, P = 0.690 Adj P = 0.777 | 0.513 | 0.673 |
| Middle Temporal Gyrus | Lt | r = -0.119, P = 0.580 Adj P = 0.745 | r = 0.198, P = 0.089 Adj P = 0.266 | r = -0.025, P = 0.856 Adj P = 0.906 | 0.214 | 0.550 |
| Middle Temporal Gyrus | Rt | r = 0.128, P = 0.551 Adj P = 0.745 | r = 0.171, P = 0.143 Adj P = 0.368 | r = 0.073, P = 0.601 Adj P = 0.777 | 0.896 | 0.923 |
| Parahippocampal Gyrus | Lt | r = -0.122, P = 0.569 Adj P = 0.745 | r = -0.078, P = 0.505 Adj P = 0.635 | **r = -0.382,** **P = 0.004** **Adj P = 0.013** | 0.151 | 0.454 |
| Parahippocampal Gyrus | Rt | r = -0.149, P = 0.486 Adj P = 0.745 | r = -0.039, P = 0.742 Adj P = 0.742 | **r = -0.384,** **P = 0.004** **Adj P = 0.013** | 0.066 | 0.397 |
| Precuneus | Lt | r = -0.012, P = 0.955 Adj P = 0.955 | r = 0.152, P = 0.193 Adj P = 0.435 | r = -0.103, P = 0.459 Adj P = 0.731 | 0.311 | 0.560 |
| Precuneus | Rt | r = -0.015, P = 0.945 Adj P = 0.955 | r = 0.074, P = 0.529 Adj P = 0.635 | **r = -0.297,** **P = 0.029** Adj P = 0.065 | 0.142 | 0.454 |
| Putamen | Lt | r = 0.371, P = 0.074 Adj P = 0.191 | r = 0.116, P = 0.322 Adj P = 0.519 | r = 0.011, P = 0.937 Adj P = 0.937 | 0.055 | 0.397 |
| Putamen | Rt | r = 0.202, P = 0.343 Adj P = 0.686 | r = 0.110, P = 0.346 Adj P = 0.519 | **r = -0.313,** **P = 0.021** Adj P = 0.055 | **0.024** | 0.397 |
| Thalamus | Lt | **r = -0.423,** **P = 0.039** Adj P = 0.142 | **r = -0.296,** **P = 0.010** **Adj P = 0.045** | **r = -0.415,** **P = 0.002** **Adj P = 0.008** | 0.908 | 0.923 |
| Thalamus | Rt | **r = -0.478,** **P = 0.018** Adj P = 0.142 | **r = -0.387,** **P < 0.001** **Adj P = 0.004** | **r = -0.421,** **P = 0.002** **Adj P = 0.008** | 0.607 | 0.729 |
| **HFC vs F_ISF_** | | | | | | |
| corpus callosum | Lt | **r = -0.445,** **P = 0.029** Adj P = 0.447 | **r = -0.637,** **P < 0.001** **Adj P < 0.001** | **r = -0.699,** **P < 0.001** **Adj P < 0.001** | 0.406 | 0.861 |
| corpus callosum | Rt | r = -0.258, P = 0.223 Adj P = 0.669 | **r = -0.616,** **P < 0.001** **Adj P < 0.001** | **r = -0.604,** **P < 0.001** **Adj P < 0.001** | 0.184 | 0.861 |
| Cuneus | Lt | r = 0.368, P = 0.077 Adj P = 0.447 | r = -0.039, P = 0.743 Adj P = 0.846 | r = 0.202, P = 0.143 Adj P = 0.304 | 0.159 | 0.861 |
| Cuneus | Rt | r = 0.017, P = 0.938 Adj P = 0.962 | r = -0.039, P = 0.741 Adj P = 0.846 | r = -0.012, P = 0.931 Adj P = 0.931 | 0.984 | 0.984 |
| Hippocampus | Lt | r = 0.219, P = 0.304 Adj P = 0.685 | r = 0.080, P = 0.493 Adj P = 0.846 | r = -0.130, P = 0.350 Adj P = 0.588 | 0.320 | 0.861 |
| Hippocampus | Rt | r = 0.232, P = 0.276 Adj P = 0.685 | r = -0.032, P = 0.785 Adj P = 0.846 | r = -0.103, P = 0.459 Adj P = 0.601 | 0.457 | 0.861 |
| Insula | Lt | r = 0.121, P = 0.572 Adj P = 0.936 | r = -0.042, P = 0.723 Adj P = 0.846 | r = 0.127, P = 0.359 Adj P = 0.588 | 0.661 | 0.930 |
| Insula | Rt | r = -0.054, P = 0.803 Adj P = 0.962 | r = -0.051, P = 0.663 Adj P = 0.846 | r = -0.064, P = 0.648 Adj P = 0.729 | 0.926 | 0.981 |
| Middle Temporal Gyrus | Lt | r = -0.033, P = 0.877 Adj P = 0.962 | r = 0.052, P = 0.661 Adj P = 0.846 | r = -0.068, P = 0.626 Adj P = 0.729 | 0.751 | 0.966 |
| Middle Temporal Gyrus | Rt | r = -0.053, P = 0.804 Adj P = 0.962 | r = 0.130, P = 0.267 Adj P = 0.846 | r = 0.101, P = 0.467 Adj P = 0.601 | 0.833 | 0.981 |
| Parahippocampal Gyrus | Lt | r = 0.085, P = 0.695 Adj P = 0.962 | r = 0.009, P = 0.938 Adj P = 0.938 | r = -0.230, P = 0.094 Adj P = 0.304 | 0.478 | 0.861 |
| Parahippocampal Gyrus | Rt | r = 0.010, P = 0.962 Adj P = 0.962 | r = -0.037, P = 0.755 Adj P = 0.846 | r = -0.199, P = 0.149 Adj P = 0.304 | 0.447 | 0.861 |
| Precuneus | Lt | r = 0.140, P = 0.514 Adj P = 0.926 | r = 0.125, P = 0.287 Adj P = 0.846 | r = -0.114, P = 0.413 Adj P = 0.601 | 0.637 | 0.930 |
| Precuneus | Rt | r = 0.175, P = 0.413 Adj P = 0.825 | r = 0.168, P = 0.150 Adj P = 0.846 | r = -0.227, P = 0.099 Adj P = 0.304 | 0.077 | 0.861 |
| Putamen | Lt | r = 0.374, P = 0.072 Adj P = 0.447 | r = 0.062, P = 0.599 Adj P = 0.846 | r = 0.051, P = 0.712 Adj P = 0.754 | 0.671 | 0.930 |
| Putamen | Rt | r = -0.024, P = 0.910 Adj P = 0.962 | r = 0.119, P = 0.309 Adj P = 0.846 | r = -0.240, P = 0.081 Adj P = 0.304 | 0.271 | 0.861 |
| Thalamus | Lt | r = -0.344, P = 0.099 Adj P = 0.447 | r = 0.030, P = 0.799 Adj P = 0.846 | r = -0.213, P = 0.123 Adj P = 0.304 | 0.464 | 0.861 |
| Thalamus | Rt | r = -0.260, P = 0.220 Adj P = 0.669 | r = -0.105, P = 0.372 Adj P = 0.846 | r = -0.198, P = 0.152 Adj P = 0.304 | 0.911 | 0.981 |

CN, MCI, and AD columns show age- and sex-adjusted associations within each diagnostic group.
Interaction P values were obtained from pooled models including predictor × diagnostic group interaction terms, adjusted for age and sex.

Adjusted P values were calculated using the Benjamini–Hochberg procedure separately for within-group associations and interaction tests across bilateral ROI tests.
P-values between 0.0001 and 0.001 are denoted as P < 0.001. P-values less than 0.0001 are denoted as P < 0.001.*

**Supplementary Table S7. Results of a receiver operating characteristic (ROC) curve analysis of MRI measures**

| **ROI** | **side** | **CN vs MCI** | | | | **CN vs AD** | | | | **MCI vs AD** | | | |
| --- | --- | --- | --- | --- | --- | --- | --- | --- | --- | --- | --- | --- | --- |
|  |  | **SE__** | **SP** | **AUC** | **p** | **SE** | **SP** | **AUC** | **p** | **SE** | **SP** | **AUC** | **p** |
| **Diffusion Interstitial Fluid (D_ISF_) *1000** | | | | | | | | | | | | | |
| corpus callosum | Lt | 54 | 77 | 0.615 | p=0.067 | **63** | **77** | **0.735** | **p<0.001*** | 80 | 41 | 0.609 | p=0.025 |
|  | Rt | 53 | 81 | 0.631 | p=0.033 | **65** | **81** | **0.739** | **p<0.001*** | 54 | 64 | 0.607 | p=0.030 |
| Cuneus | Lt | 41 | 74 | 0.531 | p=0.638 | 56 | 73 | 0.615 | p=0.089 | 67 | 53 | 0.592 | p=0.066 |
|  | Rt | 59 | 58 | 0.521 | p=0.745 | 74 | 58 | 0.621 | p=0.083 | 84 | 32 | 0.584 | p=0.088 |
| Hippocampus | Lt | 47 | 77 | 0.635 | p=0.026 | **58** | **88** | **0.785** | **p<0.001*** | 74 | 53 | 0.660 | p<0.001 |
|  | Rt | 46 | 73 | 0.573 | p=0.233 | **54** | **92** | **0.765** | **p<0.001*** | 46 | 86 | 0.694 | p<0.001* |
| Insula | Lt | 31 | 81 | 0.530 | p=0.644 | 91 | 27 | 0.575 | p=0.276 | 91 | 22 | 0.542 | p=0.402 |
|  | Rt | 60 | 58 | 0.539 | p=0.553 | 51 | 81 | 0.670 | p=0.007 | 51 | 77 | 0.642 | p=0.004 |
| Middle Temporal Gyrus | Lt | **37** | **96** | **0.621** | **p=0.035** | **56** | **96** | **0.804** | **p<0.001*** | 93 | 33 | 0.670 | p<0.001 |
|  | Rt | **49** | **92** | **0.698** | **p<0.001*** | **63** | **92** | **0.782** | **p<0.001*** | 68 | 49 | 0.583 | p=0.096 |
| Parahippocampal Gyrus | Lt | 26 | 88 | 0.520 | p=0.743 | **61** | **92** | **0.750** | **p<0.001*** | **70** | **73** | **0.721** | **p<0.001*** |
|  | Rt | 24 | 96 | 0.504 | p=0.944 | **63** | **96** | **0.790** | **p<0.001*** | **79** | **65** | **0.753** | **p<0.001*** |
| Precuneus | Lt | 74 | 50 | 0.584 | p=0.222 | 67 | 58 | 0.617 | p=0.068 | 51 | 68 | 0.548 | p=0.359 |
|  | Rt | 46 | 73 | 0.533 | p=0.616 | 58 | 69 | 0.599 | p=0.120 | 30 | 91 | 0.561 | p=0.237 |
| Putamen | Lt | 44 | 77 | 0.556 | p=0.364 | 19 | 96 | 0.524 | p=0.716 | 70 | 44 | 0.534 | p=0.496 |
|  | Rt | 81 | 38 | 0.551 | p=0.445 | 77 | 38 | 0.515 | p=0.834 | 67 | 46 | 0.525 | p=0.631 |
| Thalamus | Lt | 24 | 92 | 0.568 | p=0.284 | 77 | 54 | 0.672 | p=0.007 | 77 | 45 | 0.601 | p=0.040 |
|  | Rt | 35 | 88 | 0.520 | p=0.746 | 70 | 62 | 0.647 | p=0.021 | 75 | 54 | 0.627 | p=0.010 |
| **Diffusion Microvascularity (D_MV_) *1000** | | | | | | | | | | | | | |
| corpus callosum | Lt | 63 | 58 | 0.582 | p=0.203 | **86** | **62** | **0.771** | **p<0.001*** | 72 | 68 | 0.699 | p<0.001* |
|  | Rt | 41 | 85 | 0.611 | p=0.071 | **81** | **69** | **0.788** | **p<0.001*** | 86 | 46 | 0.672 | p<0.001 |
| Cuneus | Lt | 59 | 54 | 0.507 | p=0.916 | 81 | 54 | 0.651 | p=0.032 | 77 | 51 | 0.658 | p<0.001 |
|  | Rt | 88 | 27 | 0.514 | p=0.845 | 79 | 46 | 0.611 | p=0.130 | 63 | 64 | 0.646 | p=0.003 |
| Hippocampus | Lt | 74 | 46 | 0.601 | p=0.128 | **72** | **73** | **0.778** | **p<0.001*** | **72** | **62** | 0.693 | **p<0.001*** |
|  | Rt | 18 | 96 | 0.554 | p=0.401 | **54** | **96** | **0.789** | **p<0.001*** | **47** | **91** | **0.734** | **p<0.001*** |
| Insula | Lt | 47 | 65 | 0.524 | p=0.702 | 60 | 65 | 0.598 | p=0.147 | 100 | 19 | 0.572 | p=0.148 |
|  | Rt | 47 | 69 | 0.526 | p=0.674 | 47 | 92 | 0.682 | p=0.003 | 46 | 86 | 0.670 | p<0.001 |
| Middle Temporal Gyrus | Lt | 36 | 85 | 0.539 | p=0.530 | 61 | 85 | 0.715 | p<0.001 | 65 | 64 | 0.669 | p<0.001 |
|  | Rt | 64 | 77 | 0.688 | p<0.001 | 61 | 88 | 0.794 | p<0.001* | 58 | 71 | 0.625 | p=0.011 |
| Parahippocampal Gyrus | Lt | 47 | 65 | 0.509 | p=0.887 | **72** | **73** | **0.738** | **p<0.001*** | **75** | **64** | **0.722** | **p<0.001*** |
|  | Rt | 45 | 65 | 0.521 | p=0.752 | **65** | **81** | **0.796** | **p<0.001*** | **72** | **72** | **0.770** | **p<0.001*** |
| Precuneus | Lt | 76 | 46 | 0.603 | p=0.116 | 61 | 81 | 0.719 | p<0.001 | 44 | 85 | 0.639 | p=0.006 |
|  | Rt | 28 | 88 | 0.548 | p=0.451 | 47 | 88 | 0.683 | p=0.003 | 60 | 69 | 0.628 | p=0.009 |
| Putamen | Lt | 26 | 96 | 0.516 | p=0.796 | 61 | 65 | 0.617 | p=0.075 | 70 | 53 | 0.610 | p=0.026 |
|  | Rt | 72 | 50 | 0.571 | p=0.288 | 23 | 96 | 0.570 | p=0.300 | 56 | 71 | 0.634 | p=0.006 |
| Thalamus | Lt | 50 | 69 | 0.563 | p=0.326 | **81** | **69** | **0.766** | **p<0.001*** | 72 | 63 | 0.693 | p<0.001* |
|  | Rt | 76 | 38 | 0.518 | p=0.784 | 82 | 50 | 0.663 | p=0.013 | 81 | 53 | 0.656 | p<0.001 |
| **Diffusion Parenchyma (D_PAR_) *1000** | | | | | | | | | | | | | |
| corpus callosum | Lt | 83 | 31 | 0.555 | p=0.413 | 33 | 85 | 0.561 | p=0.366 | 30 | 86 | 0.512 | p=0.822 |
|  | Rt | 36 | 88 | 0.607 | p=0.092 | 46 | 88 | 0.621 | p=0.058 | 44 | 67 | 0.520 | p=0.699 |
| Cuneus | Lt | 31 | 81 | 0.515 | p=0.815 | 28 | 100 | 0.596 | p=0.123 | 46 | 78 | 0.599 | p=0.051 |
|  | Rt | 17 | 96 | 0.521 | p=0.739 | 51 | 77 | 0.592 | p=0.137 | 53 | 68 | 0.561 | p=0.240 |
| Hippocampus | Lt | 42 | 77 | 0.601 | p=0.103 | 53 | 85 | 0.692 | p<0.001 | 53 | 71 | 0.596 | p=0.061 |
|  | Rt | 41 | 77 | 0.571 | p=0.248 | **70** | **77** | **0.726** | **p<0.001*** | 72 | 58 | 0.656 | p=0.002 |
| Insula | Lt | 88 | 27 | 0.532 | p=0.651 | 30 | 81 | 0.511 | p=0.866 | 42 | 73 | 0.516 | p=0.761 |
|  | Rt | 96 | 15 | 0.527 | p=0.690 | 42 | 81 | 0.574 | p=0.261 | 46 | 72 | 0.549 | p=0.350 |
| Middle Temporal Gyrus | Lt | 44 | 77 | 0.563 | p=0.309 | 42 | 77 | 0.507 | p=0.908 | 42 | 72 | 0.537 | p=0.476 |
|  | Rt | 40 | 96 | 0.645 | p=0.009 | 35 | 96 | 0.619 | p=0.048 | 18 | 95 | 0.514 | p=0.781 |
| Parahippocampal Gyrus | Lt | 21 | 96 | 0.520 | p=0.739 | 40 | 88 | 0.578 | p=0.210 | 42 | 79 | 0.580 | p=0.119 |
|  | Rt | 64 | 50 | 0.524 | p=0.717 | 23 | 96 | 0.520 | p=0.751 | 47 | 69 | 0.532 | p=0.546 |
| Precuneus | Lt | 73 | 42 | 0.540 | p=0.547 | 65 | 73 | 0.662 | p=0.008 | 60 | 73 | 0.639 | p=0.007 |
|  | Rt | 88 | 23 | 0.537 | p=0.569 | 39 | 96 | 0.669 | p=0.004 | 40 | 88 | 0.637 | p=0.007 |
| Putamen | Lt | 40 | 81 | 0.566 | p=0.287 | **63** | **81** | **0.710** | **p<0.001** | 42 | 83 | 0.647 | p=0.002 |
|  | Rt | 40 | 81 | 0.516 | p=0.786 | 54 | 81 | 0.673 | p=0.003 | 49 | 79 | 0.655 | p=0.001 |
| Thalamus | Lt | 28 | 92 | 0.522 | p=0.734 | 40 | 92 | 0.639 | p=0.023 | 56 | 65 | 0.604 | p=0.038 |
|  | Rt | 23 | 96 | 0.519 | p=0.750 | 33 | 96 | 0.648 | p=0.014 | 70 | 51 | 0.605 | p=0.033 |
| **Fractional Interstitial Fluid (F_ISF_)** | | | | | | | | | | | | | |
| corpus callosum | Lt | 64 | 65 | 0.616 | p=0.079 | 54 | 65 | 0.559 | p=0.371 | 21 | 94 | 0.547 | p=0.372 |
|  | Rt | 73 | 54 | 0.619 | p=0.072 | 33 | 88 | 0.568 | p=0.294 | 39 | 78 | 0.528 | p=0.593 |
| Cuneus | Lt | 29 | 92 | 0.511 | p=0.856 | 30 | 100 | 0.611 | p=0.072 | 56 | 67 | 0.592 | p=0.072 |
|  | Rt | 44 | 69 | 0.530 | p=0.641 | 65 | 62 | 0.590 | p=0.171 | 68 | 50 | 0.546 | p=0.369 |
| Hippocampus | Lt | 81 | 46 | 0.644 | p=0.018 | 39 | 96 | 0.658 | p=0.008 | 37 | 86 | 0.542 | p=0.424 |
|  | Rt | 37 | 77 | 0.557 | p=0.370 | 58 | 77 | 0.652 | p=0.010 | 47 | 78 | 0.601 | p=0.055 |
| Insula | Lt | 90 | 27 | 0.507 | p=0.918 | 98 | 27 | 0.620 | p=0.087 | 60 | 63 | 0.619 | p=0.015 |
|  | Rt | 59 | 54 | 0.520 | p=0.774 | 53 | 62 | 0.520 | p=0.779 | 30 | 81 | 0.504 | p=0.931 |
| Middle Temporal Gyrus | Lt | 94 | 23 | 0.568 | p=0.318 | 35 | 73 | 0.506 | p=0.931 | 35 | 79 | 0.565 | p=0.202 |
|  | Rt | 58 | 77 | 0.682 | p=0.001 | 51 | 77 | 0.577 | p=0.228 | 39 | 81 | 0.585 | p=0.093 |
| Parahippocampal Gyrus | Lt | 38 | 81 | 0.595 | p=0.135 | 39 | 81 | 0.559 | p=0.359 | 39 | 74 | 0.527 | p=0.610 |
|  | Rt | 74 | 42 | 0.551 | p=0.461 | 18 | 100 | 0.565 | p=0.314 | 21 | 95 | 0.522 | p=0.679 |
| Precuneus | Lt | 19 | 96 | 0.527 | p=0.683 | 49 | 85 | 0.665 | p=0.007 | 53 | 72 | 0.630 | p=0.009 |
|  | Rt | 86 | 27 | 0.513 | p=0.848 | 61 | 69 | 0.640 | p=0.030 | 54 | 76 | 0.637 | p=0.006 |
| Putamen | Lt | 77 | 54 | 0.656 | p=0.010 | **88** | **58** | **0.776** | **p<0.001*** | 39 | 87 | 0.643 | p=0.004 |
|  | Rt | 21 | 96 | 0.517 | p=0.783 | 44 | 96 | 0.694 | p<0.001 | 40 | 87 | 0.670 | p<0.001 |
| Thalamus | Lt | 67 | 54 | 0.577 | p=0.242 | 61 | 69 | 0.676 | p=0.004 | 26 | 94 | 0.611 | p=0.027 |
|  | Rt | 67 | 54 | 0.560 | p=0.339 | 53 | 73 | 0.626 | p=0.045 | 54 | 64 | 0.569 | p=0.177 |
| **Fractional Microvascularity (F_MV_)** | | | | | | | | | | | | | |
| corpus callosum | Lt | 72 | 54 | 0.582 | p=0.216 | 63 | 65 | 0.632 | p=0.050 | 35 | 77 | 0.549 | p=0.328 |
|  | Rt | 29 | 88 | 0.551 | p=0.415 | 65 | 62 | 0.631 | p=0.042 | 61 | 54 | 0.572 | p=0.149 |
| Cuneus | Lt | 71 | 54 | 0.598 | p=0.146 | 74 | 46 | 0.538 | p=0.612 | 77 | 44 | 0.586 | p=0.080 |
|  | Rt | 87 | 42 | 0.589 | p=0.206 | 84 | 42 | 0.555 | p=0.473 | 56 | 64 | 0.570 | p=0.167 |
| Hippocampus | Lt | 29 | 88 | 0.584 | p=0.189 | 40 | 88 | 0.663 | p=0.009 | 35 | 85 | 0.590 | p=0.075 |
|  | Rt | 64 | 58 | 0.580 | p=0.219 | 26 | 100 | 0.573 | p=0.251 | 40 | 85 | 0.631 | p=0.010 |
| Insula | Lt | 17 | 96 | 0.518 | p=0.777 | 44 | 73 | 0.567 | p=0.322 | 68 | 45 | 0.537 | p=0.461 |
|  | Rt | 87 | 35 | 0.560 | p=0.394 | 12 | 100 | 0.520 | p=0.766 | 23 | 94 | 0.534 | p=0.514 |
| Middle Temporal Gyrus | Lt | 67 | 50 | 0.547 | p=0.474 | 77 | 35 | 0.524 | p=0.734 | 79 | 35 | 0.565 | p=0.192 |
|  | Rt | 26 | 92 | 0.512 | p=0.833 | 30 | 88 | 0.508 | p=0.900 | 44 | 65 | 0.511 | p=0.833 |
| Parahippocampal Gyrus | Lt | 59 | 69 | 0.620 | p=0.051 | 26 | 92 | 0.515 | p=0.816 | 51 | 73 | 0.601 | p=0.049 |
|  | Rt | 92 | 31 | 0.606 | p=0.129 | 23 | 96 | 0.505 | p=0.935 | 33 | 88 | 0.587 | p=0.096 |
| Precuneus | Lt | 41 | 77 | 0.515 | p=0.811 | 51 | 69 | 0.519 | p=0.780 | 96 | 12 | 0.504 | p=0.929 |
|  | Rt | 40 | 88 | 0.551 | p=0.383 | 54 | 77 | 0.579 | p=0.224 | 82 | 31 | 0.508 | p=0.872 |
| Putamen | Lt | 40 | 85 | 0.583 | p=0.188 | 72 | 38 | 0.520 | p=0.783 | 72 | 45 | 0.567 | p=0.176 |
|  | Rt | 64 | 62 | 0.594 | p=0.176 | 77 | 42 | 0.553 | p=0.463 | 33 | 79 | 0.550 | p=0.317 |
| Thalamus | Lt | 63 | 54 | 0.526 | p=0.695 | 82 | 54 | 0.714 | p<0.001 | 70 | 63 | 0.689 | p<0.001* |
|  | Rt | 87 | 31 | 0.557 | p=0.395 | 60 | 65 | 0.605 | p=0.133 | 60 | 76 | 0.674 | p<0.001 |
| **Fractional Parenchyma (F_PAR_)** | | | | | | | | | | | | | |
| corpus callosum | Lt | 32 | 85 | 0.576 | p=0.243 | **60** | **85** | **0.759** | **p<0.001*** | 93 | 40 | 0.690 | p<0.001* |
|  | Rt | 41 | 88 | 0.630 | p=0.037 | **68** | **88** | **0.793** | **p<0.001*** | 79 | 51 | 0.670 | p<0.001 |
| Cuneus | Lt | 53 | 65 | 0.569 | p=0.318 | 74 | 62 | 0.676 | p=0.007 | 51 | 76 | 0.627 | p=0.009 |
|  | Rt | 33 | 88 | 0.548 | p=0.450 | 68 | 62 | 0.672 | p=0.009 | 74 | 51 | 0.614 | p=0.018 |
| Hippocampus | Lt | 55 | 65 | 0.597 | p=0.100 | **74** | **85** | **0.818** | **p<0.001*** | 74 | 65 | 0.689 | p<0.001* |
|  | Rt | 64 | 65 | 0.629 | p=0.035 | **86** | **73** | **0.845** | **p<0.001*** | **77** | **68** | **0.736** | **p<0.001*** |
| Insula | Lt | 56 | 62 | 0.564 | p=0.319 | 79 | 62 | 0.709 | p<0.001 | 67 | 64 | 0.649 | p=0.002 |
|  | Rt | 31 | 88 | 0.557 | p=0.356 | 58 | 92 | 0.765 | p<0.001* | 63 | 69 | 0.694 | p<0.001* |
| Middle Temporal Gyrus | Lt | 41 | 88 | 0.606 | p=0.071 | **61** | **96** | **0.810** | **p<0.001*** | 68 | 67 | 0.684 | p<0.001* |
|  | Rt | 64 | 73 | 0.657 | p=0.006 | **79** | **77** | **0.797** | **p<0.001*** | 81 | 46 | 0.637 | p=0.004 |
| Parahippocampal Gyrus | Lt | 53 | 73 | 0.602 | p=0.083 | **79** | **88** | **0.893** | **p<0.001*** | **82** | **64** | **0.789** | **p<0.001*** |
|  | Rt | 64 | 62 | 0.610 | p=0.066 | **84** | **81** | **0.910** | **p<0.001*** | **95** | **54** | **0.804** | **p<0.001*** |
| Precuneus | Lt | 74 | 46 | 0.580 | p=0.228 | 51 | 81 | 0.648 | p=0.019 | 53 | 71 | 0.583 | p=0.109 |
|  | Rt | 59 | 62 | 0.544 | p=0.507 | 75 | 62 | 0.662 | p=0.011 | 47 | 76 | 0.620 | p=0.016 |
| Putamen | Lt | **56** | **85** | **0.714** | **p<0.001** | 63 | 85 | 0.773 | p<0.001* | 33 | 85 | 0.584 | p=0.093 |
|  | Rt | 74 | 46 | 0.608 | p=0.082 | 42 | 96 | 0.723 | p<0.001* | 39 | 90 | 0.632 | p=0.008 |
| Thalamus | Lt | 56 | 69 | 0.590 | p=0.137 | 72 | 69 | 0.740 | p<0.001* | 86 | 38 | 0.624 | p=0.009 |
|  | Rt | 33 | 88 | 0.602 | p=0.097 | 70 | 81 | 0.765 | p<0.001* | 72 | 60 | 0.636 | p=0.005 |
| **High Frequency Conductivity (HFC)** | | | | | | | | | | | | | |
| corpus callosum | Lt | 53 | 65 | 0.588 | p=0.179 | **75** | **69** | **0.746** | **p<0.001*** | 73 | 58 | 0.668 | p<0.001 |
|  | Rt | 75 | 42 | 0.595 | p=0.155 | **57** | **88** | **0.763** | **p<0.001*** | **63** | **78** | **0.701** | **p<0.001*** |
| Cuneus | Lt | 64 | 46 | 0.506 | p=0.923 | 59 | 54 | 0.529 | p=0.678 | 50 | 62 | 0.527 | p=0.596 |
|  | Rt | 66 | 54 | 0.513 | p=0.843 | 71 | 54 | 0.545 | p=0.528 | 70 | 43 | 0.528 | p=0.590 |
| Hippocampus | Lt | 18 | 96 | 0.543 | p=0.499 | 79 | 35 | 0.541 | p=0.564 | 86 | 32 | 0.584 | p=0.091 |
|  | Rt | 87 | 35 | 0.562 | p=0.384 | 98 | 15 | 0.545 | p=0.515 | 25 | 90 | 0.514 | p=0.788 |
| Insula | Lt | 40 | 81 | 0.549 | p=0.433 | 61 | 81 | **0.747** | p<0.001* | 52 | 78 | 0.690 | p<0.001* |
|  | Rt | 35 | 85 | 0.566 | p=0.285 | 59 | 88 | **0.771** | p<0.001* | **59** | **79** | **0.704** | **p<0.001*** |
| Middle Temporal Gyrus | Lt | 29 | 92 | 0.591 | p=0.140 | 38 | 77 | 0.545 | p=0.500 | 54 | 68 | 0.617 | p=0.018 |
|  | Rt | 29 | 88 | 0.544 | p=0.477 | 50 | 73 | 0.549 | p=0.454 | 57 | 65 | 0.586 | p=0.095 |
| Parahippocampal Gyrus | Lt | 30 | 81 | 0.505 | p=0.932 | 61 | 81 | 0.684 | p=0.004 | 70 | 65 | 0.670 | p<0.001 |
|  | Rt | 61 | 62 | 0.603 | p=0.140 | 70 | 62 | 0.637 | p=0.038 | 23 | 92 | 0.560 | p=0.253 |
| Precuneus | Lt | 81 | 35 | 0.529 | p=0.665 | 73 | 46 | 0.583 | p=0.214 | 48 | 71 | 0.569 | p=0.181 |
|  | Rt | 35 | 81 | 0.534 | p=0.587 | 43 | 92 | 0.658 | p=0.009 | 71 | 53 | 0.621 | p=0.016 |
| Putamen | Lt | 36 | 81 | 0.550 | p=0.445 | 25 | 88 | 0.531 | p=0.654 | 75 | 43 | 0.584 | p=0.095 |
|  | Rt | 55 | 58 | 0.522 | p=0.733 | 63 | 65 | 0.628 | p=0.058 | 80 | 45 | 0.645 | p=0.002 |
| Thalamus | Lt | 74 | 58 | 0.636 | p=0.045 | 88 | 58 | 0.751 | p<0.001* | 86 | 42 | 0.655 | p=0.001 |
|  | Rt | 57 | 73 | 0.654 | p=0.012 | 80 | 73 | **0.799** | p<0.001* | 79 | 52 | 0.655 | p=0.001 |

ROC analysis was performed for pairwise group classification (AD vs CN, AD vs MCI, and MCI vs CN). For each comparison, subjects from the two groups were selected and each MRI-derived index was evaluated separately by calculating the AUC. The optimal cutoff was determined using the Youden index, and sensitivity and specificity were reported at that cutoff. If the raw AUC was < 0.5, the score direction was flipped so that the reported AUC was ≥ 0.5. The p-value was obtained using the DeLong test for the null hypothesis AUC = 0.5 (two-sided) and reported as follows: 0.0001 < p < 0.001 as “p < 0.001”, p ≤ 0.0001 as “p < 0.001*”, otherwise as “p = 0.xxx”.

**
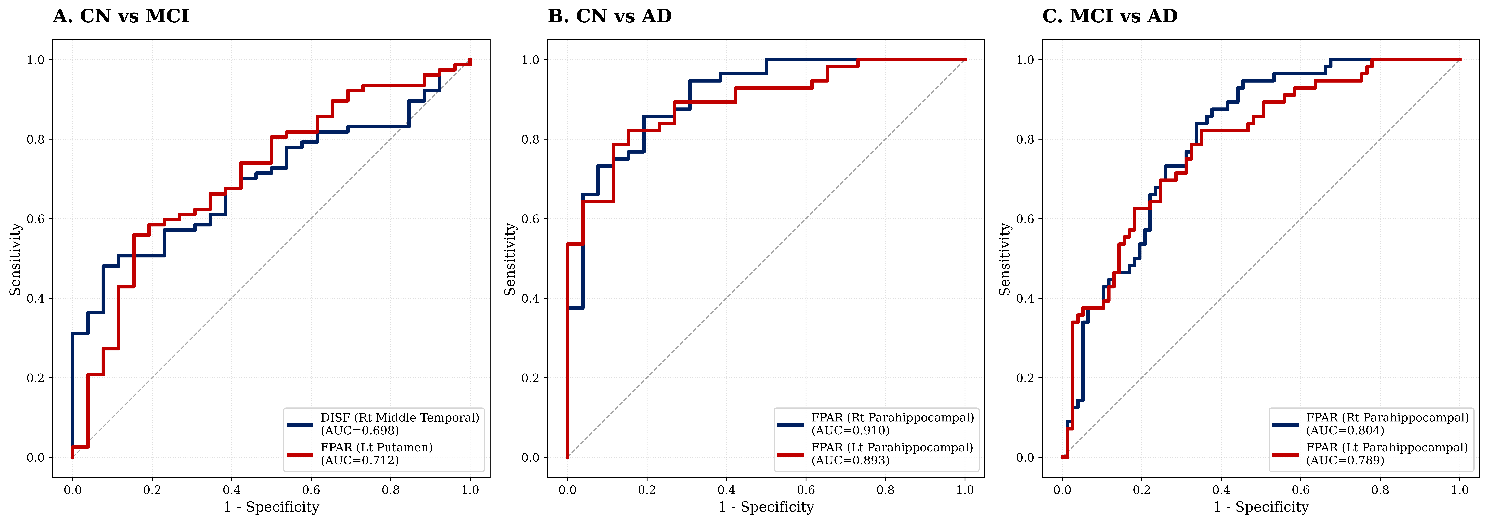
**

**Supplementary Figure S5. Receiver operating characteristic (ROC) curves of MRI measurements for group differentiation.**

The diagnostic performances of the specific conductivity and IVIM-related indices were evaluated for three comparative pairs: (A) CN vs MCI, (B) CN vs AD, and (C) MCI vs AD. The gray dashed line indicates the reference line (AUC = 0.50).

*Abbreviation: cognitively normal (CN), mild cognitive impairment (MCI), Alzheimer’s disease (AD), area under the curve (AUC), an intermediate diffusion component interpreted as ISF-related diffusion (D_ISF_), parenchymal volume fractions (F_PAR_).*
